# Supplementary material for: Efficacy, safety and cost-effectiveness of methotrexate, adalimumab or their combination in non-infectious non-anterior uveitis: a protocol for a multicentre, randomised, parallel three arms, active-controlled, phase III open label with blinded outcome assessment study
Source: BMJ Open. 2022 Mar 22;12(3):e051378. doi: 10.1136/bmjopen-2021-051378 (PMC8943738; doi:10.1136/bmjopen-2021-051378)
Supplement: Supplementary data [file bmjopen-2021-051378supp001.pdf]

Código Protocolo: Co-THEIA

Versión: 3
Fecha: 19 de octubre de 2021

HOJA DE INFORMACIÓN AL PACIENTE

Solicitamos su participación en un proyecto de investigación promovido por investigadores de su centro y dirigido por el doctor Luis Rodriguez del Hospital Clínico San Carlos (Madrid). El estudio se titula **"Eficacia, seguridad y coste-efectividad del metotrexato, adalimumab, o su combinación en uveítis no anterior no infecciosa: un estudio multicéntrico, aleatorizado, paralelo de 3 brazos, con control activo, de fase 3, abierto, con evaluador cegado: Co-THEIA (CombinationTHerapy with mEthotrexate and adalImumAb for uveitis)"**

Siguiendo los estándares internacionalmente reconocidos y la normativa legal vigente en España sobre investigación, este ensayo clínico cumple todas las exigencias legales y ha sido aprobado por el Comité Ético de Investigación con medicamentos del Hospital Clínico San Carlos así como por la Agencia Española de Medicamentos y Productos Sanitarios.

Sin embargo, antes de aceptar participar o no, es importante que lea detenidamente la siguiente información y que realice todas las preguntas y aclare todas las cuestiones que crea conveniente con un familiar/amigo o con su médico.

¿Tengo que participar?

La participación en el estudio es completamente voluntaria. Rechazarla no acarreará ningún deterioro en la calidad de la asistencia ni en su atención médica. Además, podrá retirarse en cualquier momento del estudio sin tener que dar explicaciones y sin que ello repercuta en los

Rivas AB, *et al. BMJ Open* 2022; 12:e051378. doi: 10.1136/bmjopen-2021-051378

cuidados médicos que reciba. Su médico también podrá decidir, por alguna consideración médica o por cualquier otra razón, retirarle del estudio si así lo cree conveniente.

Así mismo, el hecho de que usted participe en este estudio no altera de ninguna manera ni sus tratamientos habituales ni el seguimiento de las enfermedades que tenga (en caso de que las tuviera).

¿En qué consiste el estudio y cómo se va a llevar a cabo?

Su médico le ha diagnosticado una uveítis no infecciosa, una inflamación de una parte del ojo que no está causada por un microorganismo (bacteria, hongo o virus).

Para tratar esta inflamación es posible que su oftalmólogo y/o reumatólogo/inmunólogo/especialista en Medicina Interna ya le haya pautado distintos tratamientos, principalmente corticoides. Sin embargo, estos fármacos a veces no son suficientes para controlar enfermedad. Por otro lado, es posible que haya sido diagnosticado/a de algún tipo de uveitis que se asocie a una inflamación más persistente. En ambos casos, en la actualidad disponemos de distintos medicamentos para el tratamiento pero solo tiene indicación aprobada el adalimumab. En este estudio vamos a comparar este medicamento y el metotrexato, solos o combinados.

El metotrexato es un fármaco que inhibe el sistema inmunitario y esto permite reducir la inflación del ojo. Se lleva utilizando desde hace más de 50 años en el tratamiento de otras enfermedades inflamatorias crónicas y más reccientemente (más de 30 años) en el tratamiento de las uveitis. Se puede tomar en pastillas o inyectado en la piel (vía subcutánea), su médico según su caso le indicara como y cuando tomarlo.

El adalimumab es otro medicamente que también inhibe el sistema inmunitario y reduce la inflamación, pero utiliza otro mecanismo de acción. Se lleva utilizando en el tratamiento de las uveitis desde hace más de 10 años, administrándose por vía subcutánea.

Si usted decide participar en el estudio podrá ser asignado a uno de los tres grupos en estudio: metotrexato, adalimumab y ambos (metotrexato+adalimumab). La asignación será al azar, es decir ni usted ni su médico decidirá que tratamiento recibe. Indistintamente el grupo al que sea asignado, las visitas y las pruebas que se le realicen serán las que habitualmente se realizan a pacientes como usted. Además, en el caso de que haya sido diagnosticado de algunos subtipos de uveítis (como es la uveítis intermedia), se le realizará una resonancia magnética cerebral con gadolinio antes de iniciar el estudio. Esto es debido a que estos subtipos de uveitis podrían estar asociados a la presencia de enfermedades desmielinizantes del sistema nervioso central, en las que el uso de adalimumab se encuentra contraindicado.

Usted tendrá acudir cada 4 semanas al centro. En estas visitas su médico se encargará revisar la situación del ojo (examen del ojo, agudeza visual…), los eventos adversos y la adherencia al

tratamiento. Además, deberá completar algunos cuestionarios para evaluar su calidad de vida, los costes y los fármacos que toma (aproximadamente 15 minutos). Para completar estos cuestionarios dispondrá de ayuda del personal del centro.

Adicionalmente le solicitaremos permiso para la extracción de unas muestras de sangre al inicio, tras 16 semanas, cuando finalice y en el caso de que tuviera algún brote de la uveítis durante el estudio. Estas muestras serán utilizadas para hacer análisis genéticos y de proteómica para identificar marcadores que permitan conocer más su enfermedad y /o que puedan estar asociados a una mejor respuesta al tratamiento. También, en el caso de que fuera a recibir adalimumab, para medir los niveles en sangre de ese fármaco. Por último, con el objetivo de llevar a cabo futuros estudios que nos ayuden a conocer mejor la enfermedad que usted padece, parte de estas muestras serán depositadas y almacenadas en el Biobanco vinculado a su centro en régimen de Biobanco (por ello, en el caso de acceder a que podamos extraer estas muestras, durante el proceso asistencial, se le facilitará para su revisión y firma una copia del consentimiento informado del Biobanco vinculado a su centro). Un Biobanco es un establecimiento de almacenamiento de muestras de origen humano bajo criterios de calidad, orden y destino para su utilización en investigaciones nacionales o internacionales dentro del campo de la biomedicina. Su funcionamiento se centra en gestionar, bajo criterios de seguridad, calidad y eficiencia; la recepción, procesamiento, almacenamiento y posterior cesión de muestras a los investigadores solicitantes, para que utilicen las mismas en sus proyectos de investigación; siempre y cuando, éstos cumplan todos los requisitos éticos y legales vigentes, tal y como establece el Real Decreto 1716/2011 de 18 de noviembre, la Ley de Investigación Biomédica 14/2007, (LIBM) y la normativa que la complementa. Una vez depositadas las muestras en el Biobanco, estas pueden ser cedidas a los investigadores que hayan pasado la

aprobación de un Comité de Ética de Investigación Clínica y del Comité de Ética y del Comité Científico del Biobanco, tal y como se establece en la normativa aplicable (Real Decreto 1716/2011 de 18 de noviembre y la LIBM.

La donación de estas muestras no impedirá que usted o su familia puedan usarlas cuando sea necesario por motivos de salud, siempre que estén disponibles.

¿Puedo restringir el uso que se le de las muestras que me sean extraídas?

Usted puede indicarnos si quiere establecer algún tipo de restricción sobre sus muestras y datos, en relación con su posible uso en determinados proyectos de investigación o en cuanto a determinadas cesiones. Para ello dispone de un apartado específico en la hoja de firma del consentimiento informado.

¿Cómo sabré en que se usan mis muestras?

El Biobanco y/o la persona responsable de la investigación tendrán a disposición de los participantes la información sobre los proyectos de investigación en los que se utilicen las muestras y datos.

En determinadas circunstancias el Comité de Ética competente podrá decidir si es necesario contactar con el participante para facilitarle información de manera individualizada.

De producirse un eventual cierre del Biobanco o revocación de la autorización para su constitución y funcionamiento, la información sobre el destino de las muestras estará a su disposición en el Registro Nacional de Biobancos para Investigación Biomédica del Instituto de

Salud Carlos III (ISC III), con página web www.isciii.es, con el fin de que pueda manifestar su conformidad o disconformidad con el destino previsto para las muestras.

¿Y si obtienen alguna información clínicamente relevante e inesperada como resultado del análisis de mis muestras?

Existe la posibilidad de que las muestras que han sido cedidas al Biobanco sean utilizadas en **estudios de biología celular, molecular y/o genéticos**. En ocasiones, en este tipo de estudios se puede descubrir información no buscada que puede ser relevante para su salud o la de su familia. Si ese fuera el caso, los resultados obtenidos serán validados y analizados por profesionales y por un Comité de Ética para determinar si son fiables en un porcentaje óptimo que aconseje su comunicación a las personas afectadas.

Usted debe saber que tiene derecho a conocer, o no, la información obtenida con el análisis de sus muestras. En el caso de que usted decida no ser informado, la ley establece que cuando la información obtenida sea necesaria para evitar un grave perjuicio para la salud de sus familiares, un Comité de expertos estudiará el caso y deberá decidir entre la conveniencia o no de informar a los afectados o a sus representantes legales.

¿Qué beneficios puedo esperar por el hecho de participar en el estudio?

Puede ocurrir que usted no obtenga ningún beneficio personal de la participación en este proyecto de investigación. En cualquier caso, su participación en este estudio contribuirá al avance del conocimiento sobre el tratamiento de la uveítis no infecciosa.

¿Qué incomodidades o riesgos puede suponer mi participación en el estudio?

Entre los efectos secundarios más habituales del metotrexato están el dolor de cabeza, los moratones y sangrados, el aumento de las infecciones, las náuseas y vómitos, la diarrea, los dolores musculares, el cansancio, la pérdida de cabello y la afectación hepática.

Por su parte, el adalimumab presenta los siguientes efectos secundarios: molestias o dolor en el punto de inyección, erupción cutánea, dolor de cabeza e infecciones (siendo las más habituales las de las vías respiratorias altas (catarros entre otros) y la sinusitis).

En el caso de administrar de manera conjunta ambos fármacos los efectos adversos podrían ser más intensos y aparecer algunos desconocidos. En este momento, la administración de estos dos medicamentos de manera conjunta no está autorizada.

En el caso de que se le tuviese que realizar una resonancia nuclear magnética con gadolinio, el uso de este contraste puede verse asociado a eventos adverso agudos en 1 de cada 1000 individuos, siendo los más frecuentes de la aparición de rash, urticaria y nauseas. Además, ha sido descrita la posibilidad de depósito de esta substancia a nivel cerebral y en otros tejidos, tras administraciones repetidas.

¿Cuáles son mis derechos y cómo van a ser tratados mis datos?

Toda la información que se registrará de usted será estrictamente confidencial. De acuerdo con el Reglamento General de Protección de Datos (RGPD) (Reglamento (EU) 2016/679), además de los derechos de acceso, rectificación, oposición y cancelación de datos (Ley Orgánica 3/2018, de

5 de diciembre, de Protección de Datos Personales y garantía de los derechos digitales), también tiene derecho a limitar el tratamiento de datos y solicitar una copia o que se trasladen a un tercero (portabilidad) los datos que usted ha facilitado para el estudio. Para ejercitar sus derechos, diríjase al investigador principal del estudio. No obstante, le recordamos que los datos no se pueden eliminar, aunque deje de participar en el ensayo para garantizar la validez de la investigación y cumplir con los deberes legales y los requisitos de autorización de medicamentos. Así mismo tiene derecho a dirigirse a la Agencia de Protección de Datos si no quedara satisfecho.

En todo momento se mantendrá el anonimato de los pacientes y, para ello, en ninguno de los documentos del estudio aparecerá su nombre, sino que le será asignado un número que será el que se utilice en todos ellos. En todos los informes escritos y publicaciones, sólo aparecerá su número de referencia. Sólo el médico responsable del estudio guardará, en condiciones de seguridad, la lista que relaciona los nombres de los pacientes con los números de referencia asignados a cada uno.

Sólo tendrán acceso a los datos del estudio el equipo investigador y el monitor del estudio, miembros del comité, autoridades sanitarias competentes y/o de la agencia reguladora (AEMPS) para asegurar que el estudio se está llevando a cabo con las leyes vigentes y la reglamentación sanitaria. Firmando este documento, usted está autorizando este acceso. Además, los resultados del estudio siempre serán presentados de manera global y nunca de forma individualizada.

El Investigador y el Promotor están obligados a conservar los datos recogidos para el estudio al menos hasta 25 años tras su finalización. Posteriormente, su información personal solo se conservará por el centro para el cuidado de su salud y por el promotor para otros fines de investigación científica si usted hubiera otorgado su consentimiento para ello, y si así lo permite la ley y requisitos éticos aplicables.

Si realizáramos transferencia de sus datos codificados fuera de la UE a las entidades de nuestro grupo, a prestadores de servicios o a investigadores científicos que colaboren con nosotros, los datos del participante quedarán protegidos con salvaguardas tales como contratos u otros mecanismos por las autoridades de protección de datos. Si el participante quiere saber más al respecto, puede contactar al Delegado de Protección de Datos del promotor o Investigador Principal.

¿Mi participación supondrá algún coste o compensación económica?

Su participación en este estudio no supondrá para usted ningún coste económico, así como tampoco será recompensado económicamente por ello. El estudio dispone de una póliza de seguros que se ajusta a la legislación vigente (Real decreto 1090/2015) y que le proporcionará la compensación e indemnización en caso de menoscabo de su salud o de lesiones que pudieran producirse en relación con su participación en el estudio, siempre que no sean consecuencia de la propia enfermedad que se estudia o de la evolución propia de su enfermedad como consecuencia de la ineficacia del tratamiento. Este estudio se considera de Bajo Nivel de

Intervención (estudios de bajo riesgo, muy parecidos a la práctica clínica habitual), por lo que la póliza con la que está usted cubierto es la póliza de Sistema de Salud.

Si usted tiene alguna duda o quiere más información, no dude en consultar con el médico responsable que le está solicitando este consentimiento.

¿Cómo obtener información adicional?

Si desea información adicional sobre el estudio puede contactar con:

____

GRACIAS POR LEER ESTA INFORMACIÓN

CONSENTIMIENTO INFORMADO PARA LOS PACIENTES

Título del estudio: **"Eficacia, seguridad y coste-efectividad del metotrexato, adalimumab, o su combinación en uveítis no anterior no infecciosa: un estudio multicéntrico, aleatorizado, paralelo de 3 brazos, con control activo, de fase 3, abierto, con evaluador cegado: Co-THEIA (CombinationTHerapy with mEthotrexate and adalImumAb for uveitis)"**

Yo…………………………………………………………………………………………..

con DNI/NIF: ……………………………

(Nombre, apellidos y número de identificación del participante, puño y letra del paciente)

DECLARO:

- Que he hablado con ………………………………………………..

  (Nombre del médico, puño y letra del paciente)

- Que he leído la hoja de información que se me ha entregado sobre el estudio.
- He comprendido la información recibida y he podido formular todas las preguntas que he creído oportunas.
- Comprendo que mi participación es voluntaria y que en cualquier momento puedo revocar mi consentimiento sin tener que dar explicaciones y sin que afecte en ningún aspecto a mi relación con el personal médico ni a la atención recibida por su parte.
- He recibido una copia firmada y fechada de este Consentimiento Informado.

Presto libremente mi conformidad a participar en este estudio y doy mi consentimiento para:

La obtención de las muestras indicadas en la Hoja de Información que se me han facilitado y su cesión al Biobanco vinculado a su centro, en las condiciones indicadas, para su utilización en cualquier investigación biomédica	Si	No
Ser informado de los resultados de las investigaciones que sean de interés para mi salud<br>Teléfono de Contacto ……………………………………	Si	No
Deseo indicar restricciones al uso de los datos o de las muestras que me sean extraídas …………………………………………………………………………<br>……………………………………………………………………………<br>…………………………………………………………………………		

EN CONSECUENCIA, DOY MI CONSENTIMIENTO PARA FORMAR PARTE DE ESTE PROYECTO DE INVESTIGACION.

Firma del paciente:

Nombre y apellidos:

………………………………………….

Fecha: ……………………………

Firma del médico responsable:

Nombre y apellidos:

………………………………………….

Fecha: ……………………………
