# Supplementary material for: Efficacy, safety and cost-effectiveness of methotrexate, adalimumab or their combination in non-infectious non-anterior uveitis: a protocol for a multicentre, randomised, parallel three arms, active-controlled, phase III open label with blinded outcome assessment study
Source: BMJ Open. 2022 Mar 22;12(3):e051378. doi: 10.1136/bmjopen-2021-051378 (PMC8943738; doi:10.1136/bmjopen-2021-051378)
Supplement: Author's manuscript [file bmjopen-2021-051378.draft_revisions.pdf]

BMJ Open is committed to open peer review. As part of this commitment we make the peer review history of every article we publish publicly available.

When an article is published we post the peer reviewers' comments and the authors' responses online. We also post the versions of the paper that were used during peer review. These are the versions that the peer review comments apply to.

The versions of the paper that follow are the versions that were submitted during the peer review process. They are not the versions of record or the final published versions. They should not be cited or distributed as the published version of this manuscript.

BMJ Open is an open access journal and the full, final, typeset and author-corrected version of record of the manuscript is available on our site with no access controls, subscription charges or pay-per-view fees (http://bmjopen.bmj.com).

If you have any questions on BMJ Open's open peer review process please email info.bmjopen@bmj.com

BMJ Open

Efficacy, safety and cost-effectiveness of methotrexate, adalimumab, or their combination in non-infectious non-anterior uveitis: a protocol for a multicenter, randomized, parallel 3 arms, active-controlled, phase 3 open label with blinded outcome assessment study

Journal:	*BMJ Open*
Manuscript ID	bmjopen-2021-051378
Article Type:	Protocol
Date Submitted by the Author:	18-Mar-2021

Complete List of Authors:	Lopez-Picado, Amanda; La Fundacion para la Investigacion Biomedica del Hospital Clinico San Carlos<br>Calamia, Valentina; Instituto de Investigación Biomédica de A Coruña<br>Carreño, Ester; Hospital Universitario Fundacion Jimenez Diaz<br>Cocho, Lidia; Institute of Applied Ophtalmobiology<br>Cordero-Coma, Miguel; University Hospital Centre León<br>Fonollosa, Alex; Cruces University Hospital<br>Francisco Hernandez, Felix; Hospital Universitario de Gran Canaria Dr Negrin<br>Garcia-Aparicio, Angel; Hospital Universitario de Toledo<br>Garcia-Gonzalez, Javier; Hospital Universitario 12 de Octubre<br>Mondejar, Jose Juan; Alicante General University Hospital<br>Lojo-Oliveira, Leticia; Hospital Universitario Infanta Leonor<br>Martínez-Costa, Llucí; Hospital Universitario Doctor Peset, Ophthalmology<br>Munoz, Santiago; Hospital Universitario Infanta Sofia<br>Peiteado, Diana; Hospital Universitario La Paz<br>Pinto, Jose Antonio; Complexo Hospitalario Universitario A Coruña Servizo de Pediatría<br>Rodriguez-Lozano, Beatriz; Hospital Universitario de Canarias<br>Pato, Esperanza; Hospital Clínico San Carlos<br>Diaz-Valle, David; Hospital Clínico San Carlos<br>Molina, Elena; La Fundacion para la Investigacion Biomedica del Hospital Clinico San Carlos<br>Rodriguez-Rodriguez, Luis; La Fundacion para la Investigacion Biomedica del Hospital Clinico San Carlos,

Keywords:	Clinical trials < THERAPEUTICS, OPHTHALMOLOGY, RHEUMATOLOGY

SCHOLARONE™
Manuscripts

Efficacy, safety and cost-effectiveness of methotrexate, adalimumab, or their combination in non-infectious non-anterior uveitis: a protocol for a multicenter, randomized, parallel 3 arms, active-controlled, phase 3 open label with blinded outcome assessmentstudy

Amanda López Picado[1], Valentina Calamia[2], Ester Carreño[3], Lidia Cocho[4], Miguel Cordero-Coma[5], Alex Fonollosa[6], Felix M Francisco Hernandez[7], Angel Garcia Aparicio[8], Javier Garcia Gonzalez[9], Jose Juan Mondejar[10], Leticia Lojo Oliveira[11], Llucí Martinez-Costa[12], Santiago Muñoz[13], Diana Peiteado[14], Jose Antonio Pinto[15], Beatriz Rodriguez Lozano[16], Esperanza Pato[17], David Diaz-Valle[18], Elena Molina[19], Luis Rodriguez-Rodriguez[20], CoTHEIA Study Group.

[1] Clinical trial unit, IdISSC, Madrid (Spain), [2] Unidad de Proteómica. Grupo de Investigación de Reumatología (GIR). Instituto de Investigación Biomédica de A Coruña (INIBIC), Complexo Hospitalario Universitario de A Coruña (CHUAC), and Universidade da Coruña (UDC), A Coruña (Spain), [3]University Hospital Fundacion Jimenez Diaz, and University Hospital Rey Juan Carlos, Madrid (Spain), [4] IOBA (Institute of Applied OphthalmoBiology), University of Valladolid, and Hospital Clínico Universitario de Valladolid, Valladolid (Spain), [5] Uveitis Unit. University Hospital of León, IBIOMED, and University of León, Leon (Spain), [6] Department of Ophthalmology, BioCruces Bizkaia Health Research Institute, Cruces University Hospital, University of the Basque Country, Barakaldo, Spain, [7] Rheumatology Department, Hospital Universitario de Gran Canaria Doctor Negrín, Las Palmas de Gran Canaria (Spain), [8] Department of Rheumatology, Hospital Universitario de Toledo, Toledo (Spain), [9] Department of Rheumatology, Hospital Universitario 12 de

For peer review only - http://bmjopen.bmj.com/site/about/guidelines.xhtml

Octubre, Madrid (Spain), [10] Ophthalmology Department, Hospital General Universitario de Alicante, Alicante (Spain), [11] Sección de Reumatología, Hospital Universitario Infanta Leonor, Madrid (Spain), [12] Ophthalmology Department, Hospital Universitario Doctor Peset, Valencia (Spain), [13] Rheumatology Department, Hospital Universitario Infanta Sofia, San Sebastián de los Reyes (Spain), [14] Rheumatology Unit. Hospital Universitario La Paz, Madrid (Spain), [15] Rheumatology Department, Complexo Hospitalario Universitario de A Coruña (CHUAC), A Coruña (Spain), [16] Rheumatology Department, Hospital Universitario de Canarias, Santa Cruz de Tenerife (Spain), [17] Rheumatology Department, Hospital Clínico San Carlos, IdISSC, Madrid (Spain), [18] Servicio de Oftalmología, Hospital Clínico San Carlos. Departamento de Inmunología, Oftalmología y ORL, Facultad de Medicina, Universidad Complutense de Madrid, IdISSC, Madrid (Spain), [19] Department of Pathology, Hospital Clínico San Carlos, IdISSC, Madrid (Spain), [20] Musculoskeletal Pathology Group, Rheumatology Department, Hospital Clínico San Carlos, IdISSC, Madrid (Spain)

Correspondence

Dr Luis Rodriguez-Rodriguez, Hospital Clínico San Carlos, c\ Prof. Martín Lagos s/n, 28040, Madrid (Spain); Phone: +34913303615; email:lrrodriguez@salud.madrid.org

Word Count: 4,413

For peer review only - http://bmjopen.bmj.com/site/about/guidelines.xhtml

For peer review only

ABSTRACT

Introduction: Non-infectious uveitis (NIUs) include a heterogeneous group of sight-threatening conditions. NIUs can be incapacitating and associated with a profound impact in the quality-of-life (QoL) and wellbeing. Their correct management sometimes requires the use of immunosuppressive drugs (ISDs), which can be prescribed in monotherapy or in combination. Several observational studies have provided evidence that the use of ISDs in combination could be more effective than and as safe as their use in monotherapy. However, a direct comparison between these two treatment strategies has not been carried out yet.

Methods and analysis: The Combination THerapy with mEthotrexate and adalImumAb for uveitis (CoTHEIA) study is a phase III, multicenter, prospective, randomized, single-blinded with masked outcome assessment, parallel three arms with 1:1:1 allocation, active-controlled, superiority study design, comparing the efficacy, safety and cost-effectiveness of methotrexate (MTX), adalimumab (ADA), or their combination in non-infectious non-anterior uveitis. We aim to recruit 192 subjects. The duration of the treatment and follow-up will last up to 52 weeks. The complete and maintained resolution of the ocular inflammation will be assessed by masked evaluators (primary outcome). In addition to other secondary measurements of efficacy (QoL, visual acuity, costs) and safety, we will identify subjects' subgroups with different treatment responses by developing prediction models based on machine learning techniques using genetic and proteomic biomarkers.

Ethics and dissemination: The protocol, annexes and informed consent forms have been approved by the Reference Clinical Research Ethic Committee at the Hospital Clínico San Carlos (Madrid, Spain) and the Spanish Agency for Medicines and Health Products (AEMPS). We will elaborate a Dissemination plan including production of

For peer review only - http://bmjopen.bmj.com/site/about/guidelines.xhtml

evidence-based materials adapted to several formats to communicate the clinical trial progress and findings to a broad group of stakeholders.

Registration details: The trial is registered at clinicaltrialsregister.eu (2020-000130-18) and clinicaltrials.gov (NCT04798755).

Keywords: Uveitis, adalimumab, methotrexate, combination therapy, randomised clinical trial

For peer review only

For peer review only - http://bmjopen.bmj.com/site/about/guidelines.xhtml

ARTICLE SUMMARY

Strengths and Limitations of this Study

1. This is the first randomized controlled study designed to compare the efficacy of combination therapy versus monotherapy for the treatment of non-infectious uveitis in subjects with no previous immunosuppressive treatment.

2. We have chosen quite a strict outcome (the requirement of a maintained controlled inflammation), more likely related to long-term outcomes (such as structural damage), and to patient reported outcomes measures.

3. Despite the previous point, our primary efficacy outcome is still a surrogate marker: the achievement of this outcome does not have necessarily to translate in an improvement of outcomes more important for the patient, such as quality of life or disability.

4. The requirement to control the inflammatory process early (by week 16) may cause an underestimation of drug efficacy, as it could take more time to control the inflammation but be associated with a similar long-term prognosis.

5.The lack of masking could introduce bias, although treatment characteristics and proven effectivity in non-infectious uveitis, the duration of the trial (up to 52 weeks), and the need for bi-weekly subcutaneous injections of one of the drugs (ADA), we consider unpractical for the subject the use of placebo in the present trial.

For peer review only - http://bmjopen.bmj.com/site/about/guidelines.xhtml

INTRODUCTION

Uveitides are potentially sight-threatening diseases[1]:worldwide, they represented up to 10% of causes of blindness (almost 4 million people)[2]. Furthermore, in the European Union and the United States, after diabetic retinopathy, uveitides represent the second major treatable cause of blindness in those 20-65 years of age[3](up to 10% of cases of blind registrations[3–6]). Additionally, a high percentage of patients suffer from uveitis-related complications, visual impairment[4,7,8], and a negative impact in quality-of-life (QoL)[9,10]. Considering their higher prevalence in young to middle-aged adults[11,12] uveitides cause an important economic, social, and personal burden[5,13–16].

The correct management of NIUs is essential for preserving visual function and avoiding ocular and extra-ocular morbidity[17]. Although glucocorticoids (GCs) are the mainstay of treatment[18], under certain circumstances, adding immunosuppressive drugs (ISDs) is needed to achieve a sustained control of the inflammatory process[19]. Several ISDs are used in the standard of care, such as methotrexate (MTX), and biological agents[19–21].

Regarding MTX, its effectiveness in NIU has been assessed in two randomized clinical trials, compared with mycophenolate mofetil (MMF): Rathinam et al[22] observed that 69% and 47% of patients treated with MTX and MMF, respectively, achieved complete control of inflammation and daily oral GCs dosage ≤10mg at 5 and 6 months ($p=0.09$). In a second trial[23], randomizing 200 patients, the percentage of subjects achieving a similar outcome was 67% and 57% for MTX and MMF, respectively ($p>0.05$), with similar tolerability. Regarding safety, adverse events (AEs) are generally mild and

For peer review only - http://bmjopen.bmj.com/site/about/guidelines.xhtml

discontinuations due to serious adverse events (SAEs) are less common than for most ISDs[24][25], translating it in higher retention rates[26,27].

Regarding ADA, its effectiveness in NIU has been shown in two randomized controlled trials[28,29]. The VISUAL I study[28] compared ADA with placebo in 217 NIU patients with active uveitis despite ≥10 mg/day of systemic GCs. Based on the cumulative number of subjects with treatment failure in each visit, by week 25 (about 6 months), 31 of 107 (29%) subjects in the placebo group and 62 of 110 (56%) subjects in the ADA group had not suffered a treatment failure. By 50 weeks, the numbers were reduced to 24 (22%) and 51 (46%), respectively. Regarding safety, many of the AEs are sufficiently mild to not require discontinuation, such as injection site pain, and anti-drug antibodies formation[19,30,31], reflecting in a high retention rate[32]. Regarding SAEs, there was no association with higher risk in a recent meta-analysis[33], compared with placebo or synthetic ISDs. However, ADA was associated to a higher risk of treatment discontinuation due to SAEs. One of the most important ADA's AEs are infections. However, a previous meta-analysis reported that the absolute risk was low (0.036% with TNF-alpha inhibitors vs. 0.017% with placebo)[34], which probably does not represent a clinically important constraint on the use of these agents. Finally, ADA has not showed an association with a higher risk of malignancy[33].

Although ISDs are usually used in monotherapy in uveitis, several observational studies have shown that in 21 to 52% percentage of NIU patients, the use of ISDs in monotherapy was unable to achieve a sustained control of the inflammatory process[27,35–37].Furthermore, other studies have provided evidence that the combination of 2 or more ISDs could offer advantages in terms of effectiveness and tolerability[38–41], in conditions such as Birdshot retinochoroidopathy[38], serpiginous

For peer review only - http://bmjopen.bmj.com/site/about/guidelines.xhtml

choroiditis[39,40], Vogt-Koyanagi-Harada syndrome (VHK)[42,43], ocular Behçet's disease[44,45], JIA associated uveitis[46], sympathetic ophthalmia[47] and intermediate uveitis[48]. Regarding the beneficial of combining both MTX and ADA, several RCTs have provided evidence in other immune mediated inflammatory diseases (IMIDs)[49]. In NIU, this combinations was tested in children with Juvenile Idiopathic Arthritis-associated Uveitis and a previous failure to MTX monotherapy [50]. The combination of MTX and ADA was more effective compared to MTX and placebo, although it was associated with a higher proportion of AEs (88% vs. 83% of patients) and SAEs (22% vs. 7%).

Despite all the evidence, a direct comparison between combination and monotherapy has not been tested yet, although some groups have adopted the use of combination therapy as the initial ISD treatment for particular conditions[51].

There are currently no tools able to predict the response or non-response to ISDs in those NIUs needing immunosuppression, as there is marked interpersonal variation in their efficacy and toxicity. Response to the first-line ISD treatment could be an important predictor of long-term outcomes, as the continuous or repeated eye inflammation could increase the risk of structural permanent damage, leading to blindness, disability, and deterioration in the QoL. Therefore, starting on the right ISD is likely a key factor in achieving a better and more cost-effective therapy, and improving the optimal allocation of health care resources. To achieve these aims, objectively measured and evaluated characteristics (biomarkers[52]) are required, in addition to the identification of clinical features associated with the outcomes of interest.

For peer review only - http://bmjopen.bmj.com/site/about/guidelines.xhtml

METHODS AND ANALYSIS

Study overview

The Combination THerapy with mEthotrexate and adalImumAb for uveitis (CoTHEIA) study is a phase III, multicenter, prospective, randomized, single-blinded with masked outcome assessment, parallel 3 arms with 1:1:1 allocation, active-controlled, superiority study design, comparing the efficacy, safety and cost-effectiveness of MTX, ADA, or their combination in non-infectious non-anterior uveitis (NINAU). The duration of the treatment and follow-up will last up to 52 weeks. A 70-day follow-up clinic visit or phone call will take place to assess safety after the last study drug dose. **Figure 1** provides an overview of the study timeline and interventions.

Objectives and Outcome Variables

The trial main hypothesis is that the use of combination therapy with MTX and ADA will be more effective in inducing and maintaining ocular inflammatory inactivity than either drug given in monotherapy.

The *primary efficacy objective* is to establish which treatment strategy results in a higher proportion of subjects achieving a complete and maintained resolution of the ocular inflammation, on an intent to treat basis. The *primary efficacy outcome* will be the proportion of patients achieving a **Good Clinical Response** between the combination therapy arm and the single immunosuppressive drugs arms. This outcome is defined as a complete resolution of the ocular inflammatory signs (including active chorioretinal lesions, active retinal vascular inflammation, uveitic macular oedema, presence of anterior chamber cells, and presence of vitreous haze), achieved within the

For peer review only - http://bmjopen.bmj.com/site/about/guidelines.xhtml

first 16 weeks of the study, and maintained during follow-up until the end of the study (week 52); furthermore, there must not be a treatment failure due to safety or intolerability; the subject must adhere to the initial (up to week 16) oral GCs tapering protocol; all study visits from Baseline to 16 weeks must be completed; and at the final visit (week 52), the subject must be treated with up to 7.5mg/day of oral prednisone (or equivalent) and up to 2 times a day of prednisolone acetate 1% (or equivalent).The *secondary efficacy objectives* and outcomes variables can be found at **Table 1**.

Table 1: Efficacy-related secondary objectives and outcome variables.

Objective	Outcome variable
To compare the fraction of subjects who achieve a complete inflammatory ocular inactivity by week 16	Complete abrogation of the ocular inflammatory signs, which is achieved within the first 16 weeks of the study, no treatment failure due to safety or intolerability; compliance with the initial (up to week 16) oral GCs tapering protocol, and completion of all study visits from Baseline to 16 weeks
To compare several Patient Reported Outcomes Measures (health- and vision-related quality of life, anxiety and depression) between treatment strategies	EuroQuol 5D-5L<br>Visual Functioning Questionnaire-25<br>Hospital Anxiety and Depression Scale
To compare the presence of the clinical components of the Good Clinical Response variable between treatment strategies during follow-up	Active chorioretinal lesions; active retinal vascular inflammation; macular edema; ACC; vitreous haze; and loss of CVA secondary to inflammation at baseline, week 16, week 52, and relapse visit
To compare the time to relapse after week 16 between treatment strategies	The time to inflammatory relapse between groups, defined as the time from visit 16 weeks until end of the study, loss of follow-up or appearance of at least one ocular inflammatory manifestation, in those individuals achieving a Good Clinical Response by visit 16 week
To compare the evolution of visual acuity during follow-up	Best corrected visual acuity (BCVA) during follow-up
To compare the development of anti-adalimumab antibodies during the follow-up, between those subjects treated with monotherapy and combiation therapy	Assessment of anti-ADA antibodies (AAA) at week 15, 27, 51, and relapse.

For peer review only - http://bmjopen.bmj.com/site/about/guidelines.xhtml

To assess the cost-utility and cost-effectiveness from both a Health System and a Societal perspectives of the combination therapy and the ADA monotherapy compared with MTX given alone	Direct and indirect cost, and Incremental Cost Effectiveness Ratios

Security-related objective will establish which treatment strategy has better tolerability. Safety outcomes will be collected in the form of AEs, physical examination, and laboratory tests throughout the treatment period and up to 70 days after the last dose in this study. Gender of the subjects will be taken into account[53].

Pharmacogenetic and Proteomic-related objectives include identifying groups of subjects more likely to respond to the different treatment strategies, using genetic and proteomic biomarkers. For the former, subjects will be genotyped for known and validated genetics single nucleotide polymorphisms (SNPs) associated with a MTX[54] and ADA[55] response in different IMIDs. For the latter, a Discovery (shotgun proteomic analysis), Verification (Targeted proteomics using Multiple reaction monitoring test) and Validation (antibody-based microarrays absolute quantification tests) phases will be carried out, following optimized protocols and procedures[56–61].

Finally, a *Biobank substudy-related objective* will be carried out with the aim of boosting the translational research in the field of NIUs, by creating a collection of blood-derived samples (serum, plasma, total blood RNA, and DNA) from the participant subjects in order to advance in the identification and validation of biomarkers associated with treatment response or deepen in the pathophysiology of these conditions.

Settings and eligibility

For peer review only - http://bmjopen.bmj.com/site/about/guidelines.xhtml

The study population encompasses subjects diagnosed with non-infectious intermediate, posterior, or panuveitis with active disease within 180 days before the start of the study (Baseline visit), and either a documented failure to systemic or local GCs, or a chronic disease requiring GC-sparing ISD treatment. Main inclusion and exclusion criteria can be found at **Table 2**.

Table 2: Study participant inclusion and exclusion criteria

Inclusion criteria	Exclusion criteria
Adult subjects (≥18 years old);	Subjects with ocular histoplasmosis syndrome, or ocular masquerade syndromes;
Diagnosed with non-infectious intermediate, posterior, or panuveitis in at least one eye;	Evidence or history of malignancy;
Who have active disease within the previous 180 days before Baseline, and either	Corneal, lens or vitreous opacities precluding the visualization of the fundus;
A documented failure to systemic or local GCs in the previous 6 months, OR	Uncontrolled intraocular pressure;
A chronic disease necessitating GC-sparing immunosuppressive treatment (such as Behcet's disease with posterior segment involvement, multifocal choroiditis with panuveitis, serpiginous choroidopathy, birdshot retinochoroidopathy, diffuse retinal vasculitis, Vogt-Koyanagi-Harada with bullous serous retinal and/or choroidal detachments, or sympathetic ophthalmia;	Best corrected visual acuity (CVA) <20/400;
Able and willing to self-administer subcutaneous (SC) injections or have a qualified person available to administer SC injections; and	History, symptoms and/or Magnetic Resonance Imaging (MRI) findings suggestive of a demyelinating disease;
A negative PPD test (or equivalent) and a chest x-ray (CXR) at Screening OR if positive PPD test (or equivalent) and/or a CXR consistent with prior tuberculosis (TB) exposure, the subject must initiate, be currently receiving or have documented completion of a course of TB prophylaxis therapy, according to clinical practice.	Previous exposure to TNFi therapies;
	Previous exposure to synthetic ISDs in the previous 6 months before Baseline;
	Prior intolerability, safety issues, or ineffectiveness of MTX and/or ADA;
	Use of GCs implants (Iluvien® within 3 years, Ozurdex® within 6 months before Baseline);
	Use of intraocular or periocular GCs injection within 90 days before Baseline;

For peer review only - http://bmjopen.bmj.com/site/about/guidelines.xhtml

	Ocular surgery within 90 days before Baseline;
	Planned (elective) eye surgery in the following 52 weeks from Baseline;
	Proliferative or severe non-proliferative diabetic retinopathy;
	Neovascular/wet age-related macular degeneration;
	Chronic structural damage considered by the Investigator to interfere with measurement of macular thickness, impede the potential for its normalization, or can cause damage independent of the inflammatory process;
	Systemic inflammatory disease considered by the Investigator as likely to require high GCs dosage or prohibited medications;
	Presence of chronic recurring infections (HBV, syphilis), active TB and/or a history of invasive infection;
	Positive pregnancy test;
	Breast-feeding or considering becoming pregnant during the study.

Subjects will be recruited from 16 Spanish academic hospitals located in different regions of Spain (Galicia, País Vasco, Castilla y León, Comunidad de Madrid, Castilla La Mancha, Comunidad Valenciana, and Comunidad Canaria). In addition, the Instituto de Investigación Biomédica de A Coruña (INIBIC) will carry out the proteomic analysis, and the Instituto de Investigación Sanitaria San Carlos (IdISSC) Musculoskeletal Pathology Group will be responsible for the pharmacogenetic analysis and development of prediction models for drug response.

Recruitment

It will extend over 18 months. The participating study sites attends several tens of new patients every year, with several hundreds or thousands being followed-up, and many of these sites act as reference centres for other secondary and tertiary hospitals. Candidate subjects will be identified from patients lists AND/OR research databases AND among the new patients attended at the sites.

Subject will not receive any financial compensation for participating in the study.

For peer review only - http://bmjopen.bmj.com/site/about/guidelines.xhtml

For peer review only

To ensure subject's retention during follow-up, we will request contact details, carry out reminder phone calls/send emails, review barriers to attend appointments, and educate them in the significance of research follow-up even if they decide to discontinue the study drugs.

Intervention

All subjects entering the study will be centrally randomized at the Baseline visit into one of three study arms, in a 1:1:1 ratio:

Arm 1 will receive at the Baseline visit ADA 80 mg SC loading dose followed a week later by 40 mg every-other-week starting at Week 1. They will also receive MTX oral at the Baseline visit, with initial dose of 15mg/week, increasing up to 25 mg/week;

Arm 2: MTX with the same schedule as in Arm 1;

Arm 3: ADA with the same schedule as in Arm 1.

In addition to the study drugs, topical adjunctive eye medication will be allowed according to standard practice (intraocular pressure-lowering medication, cycloplegic agents, artificial tears, topical non-steroidal anti-inflammatory drugs…). The use of any biologic therapies with a potential therapeutic impact in NIUs, live vaccines, any other ISD besides the study drugs, intraocular GCs implants, or intraocular surgery will be prohibited for the duration of the trial.

To ensure a balance of patients across treatment groups and uveitis anatomic locations, patients will be stratified according to the main site of ocular inflammation (intermediate, OR posterior/panuveitis). Randomization will not be stratified by site due to the small-expected number of subjects per site.

Block randomization will ensure that an equal number of patients are randomized to each study arm.

For peer review only - http://bmjopen.bmj.com/site/about/guidelines.xhtml

The allocation sequence will consist of a computer-generated random number list generated and held in the Clinical Research Unit of Hospital Clínico San Carlos (HCSC), hidden from participating Investigators. The allocation sequence will be computer-generated, and it will be implemented through the electronic case report form (eCRF; REDCap), which will assign the treatment group. Study data will be collected and managed using REDCap electronic data capture tools hosted at The Health Research Institute of the Hospital Clínico San Carlos[62,63] REDCap (Research Electronic Data Capture) is a secure, web-based software platform designed to support data capture for research studies, providing 1) an intuitive interface for validated data capture; 2) audit trails for tracking data manipulation and export procedures; 3) automated export procedures for seamless data downloads to common statistical packages; and 4) procedures for data integration and interoperability with external sources. After obtaining the informed consent for enrolment and confirming that all eligibility requirements have been met, the Unmasked Investigator (see next section) will log into the eCRF and perform the randomization. Unmasked investigators will then give their assigned treatment to the subjects.

Masking

For the duration of the trial, both the study subject and the Unmasked Investigators will be aware of the treatment assigned. Considering the differences between study drugs (MTX, ADA) regarding appearance, route of administration and schedule, in order to make them unaware of the medication prescribed, the use of placebo would be necessary. However, taking into account that both drugs have been proven effective in the treatment of non-infectious uveitis, the duration of the trial (up to 52 weeks), and the need for bi-

For peer review only

For peer review only - http://bmjopen.bmj.com/site/about/guidelines.xhtml

weekly subcutaneous injections of one of the drugs (ADA), we consider unethical for the subject the use of placebo in the present trial.

Besides the Unmasked Investigators, the rest of participating investigators will be considered Masked Investigators (ophthalmologists performing the clinical eye exams, visual acuity examiners, OCT operators, fundus photographers, fundus graders, and administrators of subject's questionnaires) and will not be aware of the treatment assigned to prevent bias in study outcomes.

Several steps will be taken to avoid the Masked Investigators discovering the subject assignment: they will have no part in handling or prescribing medication; subjects will be given dark bags to place and keep their medication in throughout the trial and study visits to minimize the chances of the Masked Investigators seeing the medications. Additionally, subjects will meet with the Unmasked Investigator first, before seeing any Masked Investigators, keeping any study medication in his/her office for the entire patient visit; reviewing the appearance of any AE with the subjects before seeing any Masked Investigators, and reminding the subjects not to discuss their dosing and mediation name with the Masked Investigators.

Study Procedures

Study visits will be the Baseline visit, visits at Week 1, 4 and every 4 weeks thereafter until a) the subject is determined as unable to achieve complete resolution of the ocular inflammatory process by week 16, OR; b) the subject is determined as unable to maintain a complete resolution of the ocular inflammatory process, between weeks 16 and 52 OR; c) the subject completes 52 weeks of this clinical trial, OR; d) the study is

For peer review only - http://bmjopen.bmj.com/site/about/guidelines.xhtml

stopped due to the findings of the Data Security Monitoring Board, OR; e) the subject meets any of the study finalization criteria.

Informed consent has to be acquired before carrying out any study procedure, including screening tests (**supplementary file 1** contains a sample informed consent). Before Baseline (when the subject is randomized), several screening visits can take place up to 14 days before that visit, in order to obtain all the required complementary tests to assess eligibility. The visit window for all scheduled visits is ±3 days through Week 4 and ±7 days for all visits following the Week 4 study visit. **Table 3** provides an overview of the study activities.

After the trial, all patients will return to standard care and will be able to continue with their assigned study medication.

For peer re

Table 3: Study activities.

Activity	Screening	Baseline	w1	w4	w8	w12	w16	w20	w24	w28	w32	w36	w40	w44	w48	FET	Relapse	Unsch.	70-day Follow-up
Informed Consent	X																		
Inclusion/Exclusion Criteria	X	X																	
Medical/Surgical History	X	X																	
History of tobacco and alcohol use	X	X																	
Uveitis History	X	X																	
Vital Signs/ Weight/Height	X	X														X	(X)		
Physical Exam	X	X														X	(X)		
Symptom Directed Physical Exam			X	X	X	X	X	X	X	X	X	X	X	X	X		X		
Chest X-Ray	X															X[g]			
Cerebral MRI[a]	(X)																		
TB Screening	X																		
Serum Pregnancy Test[f]	X	X	X	X	X	X	X	X	X	X	X	X	X	X	X	X			

Hepatitis B/C Screening	X																		
Syphilis Testing (FTA)	X																		
Randomization		X																	
Best Corrected Visual Acuity Testing	(X)[b]	(X)[b]		X	X	X	X	X	X	X	X	X	X	X	X	X	X		
Tonometry	(X)[b]	(X)[b]		X	X	X	X	X	X	X	X	X	X	X	X	X	X		
Slit Lamp Exam	(X)[b]	(X)[b]		X	X	X	X	X	X	X	X	X	X	X	X	X	X		
Dilated Indirect Ophthalmoscopy	(X)[b]	(X)[b]		X	X	X	X	X	X	X	X	X	X	X	X	X	X		
Fundus Photography	(X)[b]	(X)[b]					X									X	X		
Optical Coherence Tomography	(X)[b]	(X)[b]		X	X	X	X	X	X	X	X	X	X	X	X	X	X		
EuroQol-5D	(X)[b]	(X)[b]		X	X	X	X	X	X	X	X	X	X	X	X	X	X		
Visual Functioning Questionnaire-25	(X)[b]	(X)[b]					X									X	X		
Hospital Anxiety and Depression Scale	(X)[b]	(X)[b]					X									X	X		
Hematology/Chemistry	X	(X)[c]	X	X	X	X	X		X		X		X		X	X	(X)		
Monitor Adverse Events		(X)	X	X	X	X	X	X	X	X	X	X	X	X	X	X	X	X	X
Monitor Concomitant Medication	X	X	X	X	X	X	X	X	X	X	X	X	X	X	X	X	X	X	X
Review of Patient Diary			X	X	X	X	X	X	X	X	X	X	X	X	X	X	(X)		
Perform Drug Accountability			X	X	X	X	X	X	X	X	X	X	X	X	X	X	(X)		
Assessment of direct and indirect costs			X	X	X	X	X	X	X	X	X	X	X	X	X	X	(X)		
Dispense Study Drugs		X	X	X	X	X	X	X	X	X	X	X	X	X	X		(X)		
Proteomic blood sample	(X)[d]	(X)[d]					X									X	X		
Pharmacogenetic blood sample	(X)[d]	(X)[d]																	
Adalimumab levels and Anti-Adalimumab Antibodies	(X)[d]	(X)[d]					X[e]			X[e]						X[e]	X[e]		

FET: Final (w52) / Early termination visit. Unsch: Unscheduled visit.

[a]Cerebral MRI will be performed only in those subjects with intermediate uveitis or signs of intermediate uveitis.

[b]Values from the Baseline visit or from a screening visit up to 14 days before the Baseline Visit will be used as the values from the Baseline visit.

[c]Total Blood count, renal and liver function test from up to 4 weeks before the Baseline visit will the required before entering the study.

[d]Blood sample will be drawn after signature of the informed consent, as soon as possible, ideally before start of oral GCs.

[e]Blood samples for the Anti-Adalimumab Antibodies will be drawn the week before visits 16, 28, 52, Early termination and relapse visits, up to 24h before the patient is scheduled to receive the ADA dosage, and always before the medication is taken.

[F] Only in women in childbearing

[g]Chest X ray will be performed at the 52 weeks/Early termination visit in case the patient had a positive TB test at abaseline.

Data Management and Monitoring

An Unmasked Investigator in each centre will review and crosscheck for consistency and completeness all data collected in paper within 48h of the study visit. A copy will be sent to the coordinating centre within 14 days of the subjects visit and entered by a single data entry manager into the eCRF. An external monitoring service will conduct regular checks of the data regarding errors and inconsistencies, supervising data collection, management and quality control, and will submit queries to the site investigators.

Safety

For peer review only - http://bmjopen.bmj.com/site/about/guidelines.xhtml

Non-serious AEs will be defined as an unfavorable and unintended sign (including abnormal laboratory findings), symptom or disease temporally associated with the use of the study medication or procedure, whether or not considered related to the study medication. Both MTX and ADA may be temporarily suspended in case of non-serious AEs, such as laboratory alterations, infections, and intolerance. In addition, MTX dosage may also be reduced in case of laboratory alterations and/or intolerance. Dose reductions and temporary discontinuations will be decided by the Masked Investigator, who must remain unaware of the medication and dosage assigned to the subject, so preconceptions regarding the study drugs do not interfere with their management. The Masked Investigator will issue three recommendations; one for each medication arm the subject may be included. Then, the Unmasked Investigator will implement the recommendation according to the arm the subject is included.

SAEs will be defined as any adverse event that results in death, is life threatening, requires hospitalization, or prolongation of existing hospitalization, results in persistent or significant disability or incapacity, or is a congenital anomaly or birth defect.

All AEs will be recorded in clinical records and a medically qualified investigator will assess the relationship of SAEs to the study medications.

All SAE that occur during the trial must be reported immediately by mail or fax to the Pharmacovigilance Unit within 24 hours of its occurrence. The investigator will complete and sign the SAE notification form to be sent by e-mail.

The Pharmacovigilance Unit will review the form received and, if applicable, ask for additional information to the investigator. The investigator should provide the requested information or any new information regarding the case, especially if the initial assessment in severity or causality has been changed, following the procedure previously described.

For peer review only - http://bmjopen.bmj.com/site/about/guidelines.xhtml

The Pharmacovigilance Unit is responsible for submitting as soon as possible all Suspected Unexpected Serious Adverse Reactions (SUSARs) collected during the study to the Spanish Health Authorities and Ethic Committee, according with the Spanish legislation: no later than 15 calendar days (7 in case of fatal or life-threatening cases) after first knowledge by the sponsor that the case meets the minimum criteria for expedited reporting.

Data and Safety Monitoring Committee

Only after the Data and Safety Monitoring Committee (DSMC) reviews and approves the protocol will patients be enrolled. In addition, the group will meet regularly throughout the study and review information on data quality, enrolment, patient retention, and study outcomes according to DSMC charter. The DSMC will be independent from the Sponsor, Funder and Principal Investigator, and will include experts in the fields of ophthalmology, rheumatology and epidemiology.

Adherence

Adherence will be monitored using a Patient's Diary (where they will register all study medication administered outside of the study visit (i.e., at home), including reasons for missing dosages) and by verifying the returned empty medication (partially/completely empty blisters of study medication, AND/OR cartons and sharps containers for MTX, ADA and oral GCs).

Statistical analysis plan

The Intention to treat (ITT) set will include all subjects who were randomized.

For peer review only - http://bmjopen.bmj.com/site/about/guidelines.xhtml

The safety set will consist of all subjects who received at least one dose of study medications. Per protocol analysis will also carry out. Missing data will be imputed using multiple imputation-chained equations

Sample Size Determination

ADA arm: after one year (50 weeks) of treatment, 20.3% of NIU patients achieved an outcome similar to our *primary efficacy outcome*[64];

MTX arm: after 6 months of treatment, 65% of NIU patients achieve also similar outcome[65]; we estimate that of those patients, 18%[66] and 16%[67] will be unable to maintain our outcome due to AEs and inefficacy, respectively; therefore, at 1 year, 43% will achieve our *primary efficacy outcome*.

MTX+ADA arm: we will assume that combination therapy will increase the percentage of subject achieving our *primary efficacy outcome* by 10% compared with MTX monotherapy arm.

To detect statistically significant differences between groups with a power of 80% and a significance level of 0.05, it will be necessary to recruit 54 patients per study arm (162 in total). Since the follow-up period is 52 weeks, losses of 15% will be assumed, increasing the sample size to 64 patients per study arm (192 patients in total).

Planned Methods of Statistical Analysis for efficacy-related objectives

The primary analysis will be a Mantel–Haenszel test (MHT), stratified by NIU location, comparing the combination therapy arm and the single immunosuppressive drugs arms (both arms combined), and performed in the ITT set. Achievement of the Good Clinical response will be considered the binary response variable: those subjects achieving a Good Clinical vs. those not achieving the outcome (regardless the cause). The exposure

For peer review only - http://bmjopen.bmj.com/site/about/guidelines.xhtml

variables will be the treatment arm: those subjects receiving combination therapy vs. those receiving either monotherapy. Uveitis location will be the strata: intermediate uveitis vs. posterior OR panuveitis. If the results of the primary analysis are significant, then pairwise comparisons using MHT stratified by NIU location will be carried out. P-values of the pairwise comparisons will be adjusted using the Bonferroni method. No interim efficacy-related analysis will be carried out.

Secondary efficacy-related analyses are designed to test the hypothesis that treatment assignment affects a given outcome, after controlling for selected covariates. Details can be found at **Table 4**.

Table 4: Planned Methods of Statistical Analysis for efficacy-related secondary outcomes.

Outcome variable	Statistical Analysis
Complete abrogation of the ocular inflammatory signs, which is achieved within the first 16 weeks of the study, no treatment failure due to safety or intolerability; compliance with the initial (up to week 16) oral GCs tapering protocol, and completion of all study visits from Baseline to 16 weeks	MHT, stratified by NIU location. If p-value < 0.05, pairwise comparisons using MHT stratified by NIU location will be carried out with Bonferroni adjustment of the pairwise p-values
EQ5D	GEE models nested by patient[66,67] and adjusted by study visit (continuous) and treatment arm (discrete) will be carried out, using a Gaussian family and Identity as link function. Different covariable structures will be tested (independent and exchangeable) and compared using the Bayesian Information Criteria. Time x Study arm interactions will assess different effects of time in the evolution of the outcome by arm. p-value <0.05 will be considered as a significant interaction
Visual Functioning Questionnaire-25<br>Hospital Anxiety and Depression Scale	Changes between Baseline and w16, and the FET visit will be compared between treatment groups using ANOVA
Active chorioretinal lesions; active retinal vascular lesions;	MHT, stratified by NIU location. If p-value < 0.05, pairwise comparisons using MHT stratified by NIU location will be

For peer review only - http://bmjopen.bmj.com/site/about/guidelines.xhtml

macular edema; ACC; vitreous haze; and loss of CVA secondary to inflammation at baseline, week 16, week 52, and relapse visit	carried out with Bonferroni adjustment of the pairwise p-values
The time to inflammatory relapse between groups, defined as the time from visit 16 weeks until end of the study, loss of follow-up or appearance of at least one ocular inflammatory manifestation, in those individuals achieving a Good Clinical Response by visit 16 week	Time to relapse between arms will be analyzed using log-rank test at a two-sided significance level of 5%. Dropouts due to reasons other than inability to maintain a Good Clinical Response will be considered as censored observations at the time of dropping out. Only subjects able to achieve a Good Clinical Response by visit week 16 will be analyzed. We will consider both the time until the onset of the first inflammatory manifestation, each inflammatory manifestation, the first inflammatory manifestation that does not resolve in the following 4 weeks, and each inflammatory manifestation that does not resolve in the following 4 weeks.
Best corrected visual acuity during follow-up	GEE models
Assessment of anti-ADA antibodies (AAA) at week 15, 27, 51, and relapse.	MHT, stratified by NIU location. If p-value < 0.05, pairwise comparisons using MHT stratified by NIU location will be carried out with Bonferroni adjustment of the pairwise p-values
Direct and indirect cost, and Incremental Cost Effectiveness Ratios	Cost utility and cost-effectiveness analysis from the perspectives of the National Health System and the Society will be performed. EQ5D scores will derive utility values representing health related quality of life. QALYs will be calculated by the area under the curve assuming a linear evolution of EQ5D values between visits. The average number of QALYs per patient will be calculated for each study arms. Effectiveness will be defined using our primary efficacy outcome. An average cost per patient will be calculated for each study arm, including direct (drug cost, outpatient and inpatient care, and other medical cost), and indirect cost (home care, productivity loss): Incremental cost-effectiveness ratios will be calculated, using the MTX monotherapy arm as comparison

GCs: Glucocorticoids; MTX: Methotrexate; ADA: Adalimumab; QALYs: Quality-adjusted life years; EQ5D: EuroQuol 5D-5L; GEE: Generalized estimating equations; MTH: Mantel-Haetzel; NIU: Non-infectious uveitis

Planned Methods of Statistical Analysis for Safety-related aims

The safety analysis will be performed in the safety set. Treatment-emergent AEs (events with an onset date on or after the first study drug administration until 70 days following

For peer review only - http://bmjopen.bmj.com/site/about/guidelines.xhtml

the last study drug administration) will be summarized by treatment group using descriptive statistics. SAEs with onset after informed consent but before the first study drug administration will be considered as pretreatment SAEs and reported separately. AEs will be tabulated by system organ class and preferred term whereby the most current implemented MedDRA dictionary will be used. In addition, summaries by severity and relationship to study drug will be done. Certain AEs, such as serious or severe, leading to premature withdrawal, will be listed and described in detail. AEs of special interest for treatments will be defined in the Statistical analysis plan (SAP) and analysed separately. In addition to the descriptive statistics provided, Fisher's exact test will be used for comparisons between treatment groups.

Genetic Analysis

SNPs genotypes will be determined by real-time PCR amplification using Taqman proves and following standard procedures. Duplicate genotypes of 10% of the samples, concordance (all $p > 0.05$) with the Hardy–Weinberg equilibrium and with SNP frequencies in the HapMap European collection will be used for quality control. Comparison of the proportion of subjects achieving the primary efficacy outcome between genotypes will be carried out using a χ^2 test or Fisher's exact test, when required. Dominant, recessive, and additive models of effects will be considered for each SNP. P-values will be adjusted using the Bonferroni method. Each study arm will be analysed separately.

Proteomic Analyses

Discovery Phase: Shotgun proteomic analysis[56–58] will be performed on serum samples from a representative group of subjects from each study arm with extreme

For peer review only - http://bmjopen.bmj.com/site/about/guidelines.xhtml

responses (n=20): those achieving the *primary efficacy outcome* and those not being able to achieve a good clinical response by Week 16.

Verification Phase: Targeted proteomics will be used for verification of protein markers with predictive potential in a randomly selected larger samples set (n=80). After multiple reaction monitoring (MRM) tests, relative quantification methods of the proteins will be designed[59].

Validation Phase: Best candidates from the previous phase will be validated using absolute quantification tests (antibody-based microarrays) in the whole set of subjects[60,61].

Patient subgroup identification

Based on Baseline visits patient's characteristics (demographic, disease, and clinical-related variables), those genotypes significantly associated with the primary efficacy outcome, and the previously identified serum proteins in the verification phase, prediction models for MTX, ADA, and Combination therapy response will be developed using a machine learning method (Random Forests[68]). Models' performance will be assessed with the area under the receiver operating characteristic curve, and calibration curves. Models using only clinical data, only biomarkers data, and the combination of both will be developed, to assess the contribution of biomarkers to the models' predictive ability. Due to the modest sample size we plan to recruit, we will not divide our sample in training, validation and test datasets. All subjects will be considered part of the training dataset, and a ten-fold cross-validation will be carried out to internally validate our models.

For peer review only - http://bmjopen.bmj.com/site/about/guidelines.xhtml

ETHICS AND DISSEMINATION

The protocol (version 2, 11/09/2020), annexes and informed consent forms have been approved by the Clinical Research Ethic Committee (CREC) at the Hospital Clínico San Carlos (Madrid, Spain) and the Spanish Agency for Medicines and Health Products (AEMPS). The local approvals corresponding to the participating centers will be obtained and documented before starting the study in that center as per center requirements. The promoter will be the CREC interlocutor corresponding to his/her center in everything related to the present study. It will keep CREC informed of the evolution of the study in the center and of the possible minor incidents and modifications that may occur. Any relevant modification to the protocol must receive express approval from the reference CREC and the AEMPS before its implementation, unless there are risk circumstances for the participating subjects, in which case the precise measures to ensure the integrity of the study subjects will be implemented immediately, pending the corresponding approvals. The trial is registered at clinicaltrialsregister.eu (EudraCT:2020-000130-18) and clinicaltrials.gov (NCT04798755).

No study related activities will be carried out before obtaining a written informed consent from the patient. The investigator will be responsible for a) providing each patient with an information sheet about the trial and the objectives, methods, foreseeable benefits and potential risks of the study, b) discussing the information with the patient, in terms understandable for the subject, and c) explaining to patients that they are totally free to refuse their participation in the study or to abandon it at any time and for any reason. If the subject agrees to participate in the Biobank Substudy, a second independent informed consent will be collected, which will include the possibility of storing the samples not used in the present study in the Collection of Samples for

For peer review only - http://bmjopen.bmj.com/site/about/guidelines.xhtml

Research in Rheumatic Diseases of the Rheumatology Department of the Hospital Clinico San Carlos (and, in a second phase, when the Coordinating Investigator of this study deems it appropriate and always in the event that the samples has not been used up, the remainder will be stored in the Hospital Clinico San Carlos Biobank).

All the data will be treated confidentially at any times: data will be seudonimized, the paper forms will be kept in locked cabinets, the eCRFis located in a secure server, and the person in charge of the analysis will not be able to access identification data of the subjects.

Table 5 summarises the study protocol and trial registration information.

Table 5: Trial registration data and protocol summary

Data category	Information
Primary registry and trial identifying number	EudraCT: 2020-000130-18
Date of registration in primary registry	09/03/2021
Secondary identifying numbers	ClinicalTrials.gov: NCT04798755
Source of monetary or material support	Instituto de Salud Carlos III
Primary sponsor	Fundación para la Investigación Biomédica del Hospital Clínico San Carlos
Contact for queries	Luis Rodriguez-Rodriguez, MD (lrrodriguez@salud.madrid.org)
Public Title	Combination THerapy with mEthotrexate and adalImumAb for uveitis (CoTHEIA)
Scientific title	Efficacy , safety and cost-effectiveness of methotrexate, adalimumab, or their combination in non-infectious non-anterior uveitis: a multicenter, randomized, parallel 3 arms, active-controlled, phase 3 open label with blinded outcome assessment study
Countries of recruitment	Spain
Health condition or problem studied	Non-infectious non anterior uveitis

For peer review only - http://bmjopen.bmj.com/site/about/guidelines.xhtml

Intervention(s)	Intervention 1: Adalimumab 40 mg every-other-week (plus a 80 mg SC loading dose) + Methotrexate oral, up to 25 mg/week, both for a duration of 52 weeks.
	Intervention 2: Methotrexate with the same schedule as in Intervention 1
	Intervention 3: Adalimumab with the same schedule as in Intervention 1
Key inclusion and exclusion criteria	Age ≥18 years old<br>Diagnosed with non-infectious intermediate, posterior, or panuveitis in at least one eye;<br>Active ocular disease<br>Lack of satisfactory response to systemic and/or local glucocorticoid (GC) therapy AND/OR diagnoses of a chronic disease usually necessitating GC-sparing immunosuppressive treatment.
Study type	Phase III, multicenter, prospective, randomized, single-blinded with masked outcome assessment, parallel 3 arms with 1:1:1 allocation, active-controlled, superiority study design
Date of first enrolment	N/A
Target sample size	64 per treatment arm (192 in total)
Recruitment status	Not yet recruiting
Primary outcome	Complete resolution of the ocular inflammatory signs, which is achieved within the first 16 weeks of the study, and maintained during follow-up until the end of the study (week 52);
Key secondary outcomes	Safety, cost-effectiveness

Regardingdissemination, in order to communicate the clinical trial progress and findings to a broad group of stakeholders, we will elaborate a Dissemination plan which will include production of evidence-based materials adapted to scientific meetings, scientific publications, patients, and other stakeholders; online dissemination, web and social media; and press dissemination.A summary of the final version of the study protocol will be made available through the Project's Website. The promoter will be the only with access to the participant-level data, following the regulation on data protection. The promoter can share the data with other researchers upon request and within the legal situation.

For peer review only - http://bmjopen.bmj.com/site/about/guidelines.xhtml

CONTRIBUTORSHIP STATEMENT

LRR, ALP, EPC and DDV conceived of the study.

LRR, ALP, EPC, DDV, ECS, MCC, AFC, AGA, JGG, SMM, initiated the study design and helped with implementation.

LRR is the funding holder.

ALP provided statistical expertise in clinical trial design.

VC provided expertise regarding the proteomic analysis design.

LRR provided expertise regarding the pharmacogenetic analysis.

ALP and LRR will conduct the primary statistical analysis.

ALP, VC, ECS, LCA, MCC, AFC, FMFH, AGA, JGG, JJMG, LLO, LMC, SMM, DPL, JAPT, BRL, EPC, DDV, EMR and LRR contributed to refinement of the study protocol and approved the final manuscript.

COMPETING INTERESTS

None declared

FUNDING

This work was supported by the Instituto de Salud Carlos III, grant number [ICI19/00020].

REFERENCES

1 Caspi RR. A look at autoimmunity and inflammation in the eye. *J Clin Invest*

For peer review only - http://bmjopen.bmj.com/site/about/guidelines.xhtml

2010;**120**:3073–83. doi:10.1172/JCI42440

2 WHO. The World Health Report, Global data on visual impairments. 2010.http://www.who.int/blindness/GLOBALDATAFINALforweb.pdf

3 Suttorp-Schulten MS, Rothova A. The possible impact of uveitis in blindness: a literature survey. *Br J Ophthalmol* 1996;**80**:844–8. doi:10.1136/bjo.80.9.844

4 Rothova A, Suttorp-van Schulten MS, Frits Treffers W, *et al.* Causes and frequency of blindness in patients with intraocular inflammatory disease. *Br J Ophthalmol* 1996;**80**:332–6.http://www.pubmedcentral.nih.gov/articlerender.fcgi?artid=505460&tool=pmcentrez&rendertype=abstract (accessed 25 Jul 2014).

5 de Smet MD, Taylor SRJ, Bodaghi B, *et al.* Understanding uveitis: The impact of research on visual outcomes. *Prog Retin Eye Res* 2011;**30**:452–70. doi:10.1016/j.preteyeres.2011.06.005

6 Williams GJ, Brannan S, Forrester J V, *et al.* The prevalence of sight-threatening uveitis in Scotland. *Br J Ophthalmol* 2007;**91**:33–6. doi:10.1136/bjo.2006.101386

7 Gritz DC, Wong IG. Incidence and prevalence of uveitis in Northern California: The Northern California Epidemiology of Uveitis Study. Ophthalmology. 2004;**111**:491–500. doi:10.1016/j.ophtha.2003.06.014

8 Chang JH-M, Wakefield D. Uveitis: a global perspective. *Ocul Immunol Inflamm* 2002;**10**:263–79. doi:10.1076/ocii.10.4.263.15592

9 Arriola-Villalobos P, Abásolo L, García-Feijoo J, *et al.* Vision-related Quality of Life in Patients with Non-infectious Uveitis: A Cross-sectional Study. *Ocul Immunol Inflamm* 2017;:1–9. doi:10.1080/09273948.2017.1285034

10 Miserocchi E, Modorati G, Mosconi P, *et al.* Quality of life in patients with

For peer review only

For peer review only - http://bmjopen.bmj.com/site/about/guidelines.xhtml

uveitis on chronic systemic immunosuppressive treatment. *Ocul Immunol Inflamm* 2010;**18**:297–304. doi:10.3109/09273941003637510

11 DARRELL RW, WAGENER HP, KURLAND LT. Epidemiology of uveitis. Incidence and prevalence in a small urban community. *Arch Ophthalmol (Chicago, Ill 1960)* 1962;**68**:502–14.http://www.ncbi.nlm.nih.gov/pubmed/13883604

12 Suhler EB, Lloyd MJ, Choi D, *et al.* Incidence and prevalence of uveitis in Veterans Affairs Medical Centers of the Pacific Northwest. *Am J Ophthalmol* 2008;**146**:890-6.e8. doi:10.1016/j.ajo.2008.09.014

13 E. Kirbach, S., A. Hayes, O., A. Cifaldi M. The Economic Burden of Uveitis. *Arthritis Rheum* 2010;**62**:788. doi:10.1002/art.28556

14 Adán-Civera AM, Benítez-Del-Castillo JM, Blanco-Alonso R, *et al.* Burden and direct costs of non infectious uveitis in Spain. *Reumatol Clin* 2015;**12**:196–200. doi:10.1016/j.reuma.2015.08.004

15 Durrani OM, Meads CA, Murray PI. Uveitis: A potentially blinding disease. Ophthalmologica. 2004;**218**:223–36. doi:10.1159/000078612

16 Jalil A, Yin K, Coyle L, *et al.* Vision-related quality of life and employment status in patients with uveitis of working age: a prospective study. *Ocul Immunol Inflamm* 2012;**20**:262–5. doi:10.3109/09273948.2012.684420

17 Dick AD, Rosenbaum JT, Al-Dhibi HA, *et al.* Guidance on Noncorticosteroid Systemic Immunomodulatory Therapy in Noninfectious Uveitis. *Ophthalmology* 2018;**125**:757–73. doi:10.1016/j.ophtha.2017.11.017

18 Smith JR, Rosenbaum JT. Management of uveitis: a rheumatologic perspective. *Arthritis Rheum* 2002;**46**:309–18. doi:10.1002/art.503

19 Jabs D a, Rosenbaum JT, Foster CS, *et al.* Guidelines for the use of

For peer review only - http://bmjopen.bmj.com/site/about/guidelines.xhtml

immunosuppressive drugs in patients with ocular inflammatory disorders: recommendations of an expert panel. *Am J Ophthalmol* 2000;**130**:492–513. doi:10.1016/S0002-9394(00)00659-0

20 Becker MD, Smith JR, Max R, *et al.* Management of sight-threatening uveitis: new therapeutic options. *Drugs* 2005;**65**:497–519. doi:10.2165/00003495-200565040-00005

21 Jabs DA. Immunosuppression for the Uveitides. *Ophthalmology* 2018;**125**:193–202. doi:10.1016/j.ophtha.2017.08.007

22 Rathinam SR, Babu M, Thundikandy R, *et al.* A randomized clinical trial comparing methotrexate and mycophenolate mofetil for noninfectious uveitis. *Ophthalmology* 2014;**121**:1863–70. doi:10.1016/j.ophtha.2014.04.023

23 Rathinam SR, Gonzales JA, Thundikandy R, *et al.* Effect of Corticosteroid-Sparing Treatment With Mycophenolate Mofetil vs Methotrexate on Inflammation in Patients With Uveitis: A Randomized Clinical Trial. *JAMA* 2019;**322**:936–45. doi:10.1001/jama.2019.12618

24 Gangaputra S, Newcomb CW, Liesegang TL, *et al.* Methotrexate for Ocular Inflammatory Diseases. *Ophthalmology* 2009;**116**:2188–99. doi:10.1016/j.ophtha.2009.04.020

25 Salliot C, van der Heijde D. Long-term safety of methotrexate monotherapy in patients with rheumatoid arthritis: a systematic literature research. *Ann Rheum Dis* 2009;**68**:1100–4. doi:10.1136/ard.2008.093690

26 Baker KB, Spurrier NJ, Watkins AS, *et al.* Retention time for corticosteroid-sparing systemic immunosuppressive agents in patients with inflammatory eye disease. *Br J Ophthalmol* 2006;**90**:1481–5. doi:10.1136/bjo.2006.097998

27 Abásolo L, Rosales Z, Díaz-Valle D, *et al.* Immunosuppressive Drug

For peer review only - http://bmjopen.bmj.com/site/about/guidelines.xhtml

Discontinuation in Noninfectious Uveitis From Real-Life Clinical Practice: A Survival Analysis. *Am J Ophthalmol* 2016;**169**:1–8. doi:10.1016/j.ajo.2016.06.007

28 Jaffe GJ, Dick AD, Brézin AP, *et al.* Adalimumab in Patients with Active Noninfectious Uveitis. *N Engl J Med* 2016;**375**:932–43. doi:10.1056/NEJMoa1509852

29 Nguyen QD, Merrill PT, Jaffe GJ, *et al.* Adalimumab for prevention of uveitic flare in patients with inactive non-infectious uveitis controlled by corticosteroids (VISUAL II): a multicentre, double-masked, randomised, placebo-controlled phase 3 trial. *Lancet (London, England)* 2016;**388**:1183–92. doi:10.1016/S0140-6736(16)31339-3

30 Arida A, Fragiadaki K, Giavri E, *et al.* Anti-TNF agents for Behçet's disease: analysis of published data on 369 patients. *Semin Arthritis Rheum* 2011;**41**:61–70. doi:10.1016/j.semarthrit.2010.09.002

31 Leinonen ST, Aalto K, Kotaniemi KM, *et al.* Anti-adalimumab antibodies in juvenile idiopathic arthritis-related uveitis. *Clin Exp Rheumatol*;**35**:1043–6.http://www.ncbi.nlm.nih.gov/pubmed/29148422

32 Llorenç V, Cordero-Coma M, Blanco-Esteban A, *et al.* Drug Retention Rate and Causes of Discontinuation of Adalimumab in Uveitis: Real-World Data from the Biotherapies in Uveitis (BioÚvea) Study Group. *Ophthalmology* 2020;**127**:814–25. doi:10.1016/j.ophtha.2019.11.024

33 Michaud TL, Rho YH, Shamliyan T, *et al.* The comparative safety of tumor necrosis factor inhibitors in rheumatoid arthritis: a meta-analysis update of 44 trials. *Am J Med* 2014;**127**:1208–32. doi:10.1016/j.amjmed.2014.06.012

34 Bongartz T, Sutton AJ, Sweeting MJ, *et al.* Anti-TNF antibody therapy in

For peer review only - http://bmjopen.bmj.com/site/about/guidelines.xhtml

rheumatoid arthritis and the risk of serious infections and malignancies: systematic review and meta-analysis of rare harmful effects in randomized controlled trials. *JAMA* 2006;**295**:2275–85. doi:10.1001/jama.295.19.2275

35 Joshi L, Talat L, Yaganti S, *et al.* Outcomes of changing immunosuppressive therapy after treatment failure in patients with noninfectious uveitis. *Ophthalmology* 2014;**121**:1119–24. doi:10.1016/j.ophtha.2013.11.032

36 Menezo V, Lau C, Comer M, *et al.* Clinical outcome of chronic immunosuppression in patients with non-infectious uveitis. *Clin Experiment Ophthalmol* 2005;**33**:16–21. doi:10.1111/j.1442-9071.2005.00904.x

37 Goldberg NR, Lyu T, Moshier E, *et al.* Success with single-agent immunosuppression for multifocal choroidopathies. *Am J Ophthalmol* 2014;**158**:1310–7. doi:10.1016/j.ajo.2014.08.039

38 Cervantes-Castañeda RA, Gonzalez-Gonzalez LA, Cordero-Coma M, *et al.* Combined therapy of cyclosporine A and mycophenolate mofetil for the treatment of birdshot retinochoroidopathy: a 12-month follow-up. *Br J Ophthalmol* 2013;**97**:637–43. doi:10.1136/bjophthalmol-2012-302123

39 Akpek EK, Baltatzis S, Yang J, *et al.* Long-term immunosuppressive treatment of serpiginous choroiditis. *Ocul Immunol Inflamm* 2001;**9**:153–67.http://www.ncbi.nlm.nih.gov/pubmed/11815884

40 Hooper PL, Kaplan HJ. Triple agent immunosuppression in serpiginous choroiditis. *Ophthalmology* 1991;**98**:944–51; discussion 951-2.http://www.ncbi.nlm.nih.gov/pubmed/1866149

41 Pascalis L, Pia G, Aresu G, *et al.* Combined cyclosporin A-steroid-MTX treatment in endogenous non-infectious uveitis. *J Autoimmun* 1993;**6**:467–80. doi:10.1006/jaut.1993.1039

For peer review only

42 Agarwal M, Ganesh SK, Biswas J. Triple agent immunosuppressive therapy in Vogt-Koyanagi-Harada syndrome. *Ocul Immunol Inflamm* 2006;**14**:333–9. doi:10.1080/09273940600976938

43 Arcinue CA, Radwan A, Lebanan MO, *et al.* Comparison of two different combination immunosuppressive therapies in the treatment of Vogt-Koyonagi-Harada syndrome. *Ocul Immunol Inflamm* 2013;**21**:47–52. doi:10.3109/09273948.2012.728668

44 Davatchi F, Sadeghi Abdollahi B, Shams H, *et al.* Combination of pulse cyclophosphamide and azathioprine in ocular manifestations of Behcet's disease: longitudinal study of up to 10 years. *Int J Rheum Dis* 2014;**17**:444–52. doi:10.1111/1756-185X.12248

45 Katsuyama A, Kusuhara S, Nishisho R, *et al.* Long-term efficacy and safety of infliximab and cyclosporine combination therapy for refractory uveoretinitis in Behçet's disease. *Clin Ophthalmol* 2019;**13**:521–7. doi:10.2147/OPTH.S198648

46 Kotaniemi K. Late onset uveitis in juvenile-type chronic polyarthritis controlled with prednisolone, cyclosporin A and methotrexate. *Clin Exp Rheumatol*;**16**:469–71.http://www.ncbi.nlm.nih.gov/pubmed/9706430

47 Ganesh SK, Narayana KM, Biswas J. Peripapillary choroidal atrophy in sympathetic ophthalmia and management with triple-agent immunosuppression. *Ocul Immunol Inflamm* 2003;**11**:61–5.http://www.ncbi.nlm.nih.gov/pubmed/12854028

48 Cimino L, Finzi G, Mora P, *et al.* Effect of a drug combination treatment on ocular perfusion in recurrent idiopathic intermediate uveitis. *Ocul Immunol Inflamm* 2003;**11**:299–303.http://www.ncbi.nlm.nih.gov/pubmed/14704901

49 Hazlewood GS, Barnabe C, Tomlinson G, *et al.* Methotrexate monotherapy and

For peer review only - http://bmjopen.bmj.com/site/about/guidelines.xhtml

methotrexate combination therapy with traditional and biologic disease modifying antirheumatic drugs for rheumatoid arthritis: abridged Cochrane systematic review and network meta-analysis. *BMJ* 2016;**353**:i1777.http://www.ncbi.nlm.nih.gov/pubmed/27102806

50 Ramanan A V, Dick AD, Jones AP, *et al.* Adalimumab plus Methotrexate for Uveitis in Juvenile Idiopathic Arthritis. *N Engl J Med* 2017;**376**:1637–46. doi:10.1056/NEJMoa1614160

51 Kiss S, Ahmed M, Letko E, *et al.* Long-term follow-up of patients with birdshot retinochoroidopathy treated with corticosteroid-sparing systemic immunomodulatory therapy. *Ophthalmology* 2005;**112**:1066–71. doi:10.1016/j.ophtha.2004.12.036

52 Biomarkers Definitions Working Group. Biomarkers and surrogate endpoints: preferred definitions and conceptual framework. *Clin Pharmacol Ther* 2001;**69**:89–95. doi:10.1067/mcp.2001.113989

53 LaMattina KC, Escott SM, Malalis JF, *et al.* The Influence of Race and Sex on the Side Effect Profile of Methotrexate in the Treatment of Uveitis. *Ocul Immunol Inflamm* 2018;:1–5. doi:10.1080/09273948.2018.1498905

54 López-Rodríguez R, Ferreiro-Iglesias A, Lima A, *et al.* Replication study of polymorphisms associated with response to methotrexate in patients with rheumatoid arthritis. *Sci Rep* 2018;**8**:7342. doi:10.1038/s41598-018-25634-y

55 Bek S, Bojesen AB, Nielsen J V, *et al.* Systematic review and meta-analysis: pharmacogenetics of anti-TNF treatment response in rheumatoid arthritis. *Pharmacogenomics J* 2017;**17**:403–11. doi:10.1038/tpj.2017.26

56 Fernández-Puente P, Mateos J, Fernández-Costa C, *et al.* Identification of a panel of novel serum osteoarthritis biomarkers. *J Proteome Res* 2011;**10**:5095–101.

For peer review only - http://bmjopen.bmj.com/site/about/guidelines.xhtml

doi:10.1021/pr200695p

57 Lourido L, Calamia V, Mateos J, *et al.* Quantitative proteomic profiling of human articular cartilage degradation in osteoarthritis. *J Proteome Res* 2014;**13**:6096–106. doi:10.1021/pr501024p

58 Lourido L, Calamia V, Fernández-Puente P, *et al.* Secretome analysis of human articular chondrocytes unravels catabolic effects of nicotine on the joint. *Proteomics Clin Appl* 2016;**10**:671–80. doi:10.1002/prca.201400186

59 Fernández-Puente P, Calamia V, González-Rodríguez L, *et al.* Multiplexed mass spectrometry monitoring of biomarker candidates for osteoarthritis. *J Proteomics* 2017;**152**:216–25. doi:10.1016/j.jprot.2016.11.012

60 Lourido L, Ayoglu B, Fernández-Tajes J, *et al.* Discovery of circulating proteins associated to knee radiographic osteoarthritis. *Sci Rep* 2017;**7**:137. doi:10.1038/s41598-017-00195-8

61 Drobin K, Nilsson P, Schwenk JM. Highly multiplexed antibody suspension bead arrays for plasma protein profiling. *Methods Mol Biol* 2013;**1023**:137–45. doi:10.1007/978-1-4614-7209-4_8

62 Harris PA, Taylor R, Thielke R, *et al.* Research electronic data capture (REDCap)--a metadata-driven methodology and workflow process for providing translational research informatics support. *J Biomed Inform* 2009;**42**:377–81. doi:10.1016/j.jbi.2008.08.010

63 Harris PA, Taylor R, Minor BL, *et al.* The REDCap consortium: Building an international community of software platform partners. *J Biomed Inform* 2019;**95**:103208. doi:10.1016/j.jbi.2019.103208

64 Jaffe GJ, Dick AD, Brézin AP, *et al.* Adalimumab in Patients with Active Noninfectious Uveitis. *N Engl J Med* 2016;**375**:932–43.

For peer review only - http://bmjopen.bmj.com/site/about/guidelines.xhtml

doi:10.1056/NEJMoa1509852

65 NCT01829295: Methotrexate and Mycophenolate Mofetil for UVEITIS (FAST). https://clinicaltrials.gov/ct2/show/study/NCT01829295?cond=Uveitis&draw=2&rank=35

66 Samson CM, Waheed N, Baltatzis S, *et al.* Methotrexate therapy for chronic noninfectious uveitis: Analysis of a case series of 160 patients. *Ophthalmology* 2001;**108**:1134–9.

67 Galor A, Jabs DA, Leder HA, *et al.* Comparison of antimetabolite drugs as corticosteroid-sparing therapy for noninfectious ocular inflammation. *Ophthalmology* 2008;**115**:1826–32. doi:10.1016/j.ophtha.2008.04.026

68 Madrid-García A, Font-Urgelles J, Vega-Barbas M, *et al.* Outpatient Readmission in Rheumatology: A Machine Learning Predictive Model of Patient's Return to the Clinic. *J Clin Med* 2019;**8**. doi:10.3390/jcm8081156

For peer review only

For peer review only - http://bmjopen.bmj.com/site/about/guidelines.xhtml

FIGURE LEGENDS

Figure 1: Overview of the study timeline and interventions.

ACKNOWLEDGEMENTS

Patient and Public Involvement

No patient involved

Sponsor

Fundación para la Investigacion Biomédica del Hospital Clínico San Carlos. The study sponsor had no role in the study design; collection, management, analysis and interpretation of data; writing of the report and the decision to submit the report for publication.

Committees

Executive Committee: Administrative and executive arm of the clinical trial, providing overall oversight for the study and making decisions on day-to-day operational issues [Study Coordinator (Luis Rodriguez-Rodriguez), a representative from the Spanish Clinical Trial Network (Amanda López Picado), and 5 Site Directors (these seats will be rotatory, with changes every 6 months)]; Data Coordinating and Analysis Committee: Supervising data collection,management and quality control, designing the statistical analysis plan, performing unmasked data analysis, and preparing interim and final reports for the Data Security Monitoring Board and the Executy Committee [Study

For peer review only

Coordinator (Luis Rodriguez-Rodriguez), a representative from the Spanish Clinical Trial Network (Amanda López Picado), and Ester Carreño]; Biobank and Biomarker Identification Committee (Maintaining an up-to-date manual of operations for blood extraction, processing and storage, and monitoring procedures adherence, supervising biological sample collection, sample shipment coordination, coordinating the phamacogenetic and proteomic analysis [Study Coordinator (Luis Rodriguez-Rodriguez), a representative from the Instituto de Salud Carlos III Biobank Platform (Elena Molino), a representative the Instituto de Investigación Biomédica de A Coruña, a representative from, the Data Coordinating and Analysis Committee]; Data Security Monitoring Committee (PierGiogio Neri, Andrew Dick, Loreto Carmona).

Patient consent for publication

Not required.

CoTHEIA Study Group

Mara Albert Fort (Hospital Universitario Doctor Peset), Mayte Ariño Gutierrez (Hospital Clínico San Carlos), Pedro Arriola Villalobos (Hospital Clínico San Carlos), Joseba Artaraz Beobide (Hospital Universitario Cruces), Antonio Atanes Sandoval (Complejo Hospitalario Universitario A Coruña), Jose Manuel Benitez Del Castillo Sanchez (Hospital Clínico San Carlos), Francisco Blázquez (Instituto Universitario de Oftalmología Aplicada, Universidad de Valladolid), Pablo Borges Deniz (Hospital Universitario Fundación Jiménez Díaz), Lara Borrego Sanz (Hospital Clínico San Carlos), Gabriela Bustamante Sanchez-Arnedo (Instituto Universitario de Oftalmología Aplicada, Universidad de Valladolid), Bruno Casco Silva (Complejo Hospitalario Universitario de Toledo), Ricardo Cuiña Sardiña (Hospital Clínico San Carlos),

For peer review only - http://bmjopen.bmj.com/site/about/guidelines.xhtml

Almudena De Pablo Cabrera (Hospital Universitario 12 de Octubre), Maria Del Mar Esteban Ortega (Hospital Universitario Infanta Sofía), Patricia Fernandez Puente (Instituto de Investigación Biomédica de A Coruña), Dalifer Dayanira Freites Nuñez (Hospital Clínico San Carlos), Javier Garcia Bella (Hospital Clínico San Carlos), Sara Garcia Carazo (Hospital Universitario La Paz), Luis Garcia Onrubia (Instituto Universitario de Oftalmología Aplicada, Universidad de Valladolid), Jose Maria Garcia Ruiz De Morales (Complejo Asistencial Universitario de León), Jose Antonio Gegundez Fernandez (Hospital Clínico San Carlos), Vanesa Hernandez Hernandez (Complejo Hospitalario Universitario de Canarias), Inés Hernanz Rodriguez (Hospital Universitario Fundación Jiménez Díaz), Jose Maria Herreras Cantalapiedra (Instituto Universitario de Oftalmología Aplicada, Universidad de Valladolid), Ventura Hidalgo Barrero (Hospital Universitario La Paz), Maria De La Vega Jovani Casano (Hospital General Universitario de Alicante), Maria Isabel Lopez Rodriguez (Complejo Hospitalario Universitario A Coruña), Sara López Sierra (Hospital Universitario Doctor Peset), Virginia Lozano Lopez (Complejo Hospitalario Universitario de Canarias), Alfredo Madrid Garcia (Hospital Clínico San Carlos), Rosalia Mendez Fernandez (Hospital Clínico San Carlos), Pilar Nozal Aranda (Hospital Universitario La Paz), Eugenio Perez Blazquez (Hospital Universitario 12 de Octubre), Chamaida Plasencia Rodriguez (Hospital Universitario La Paz), Sheila Recuero Diaz (Hospital Universitario Fundación Jiménez Díaz), Ignacio Robles Barrena (Hospital Universitario Rey Juan Carlos), Mª Esther Rodriguez Almaraz (Hospital Universitario 12 de Octubre), Fayna Maria Rodriguez Gonzalez (Hospital Universitario de Gran Canaria Doctor Negrín), Guadalupe Rodriguez Martinez (Complejo Hospitalario Universitario A Coruña), Fredeswinda Isabel Romero Bueno (Hospital Universitario Fundación Jiménez Díaz), Ioana Ruiz Arruza (Hospital Universitario Cruces), Beatriz Sanchez Marugan (Hospital

For peer review only

For peer review only - http://bmjopen.bmj.com/site/about/guidelines.xhtml

Universitario Infanta Leonor), Olga Sanchez Pernaute (Hospital Universitario Fundación Jiménez Díaz), Armelle Schlinker Giraud (Hospital Universitario La Paz), Alvaro Seijas Lopez (Complejo Hospitalario Universitario A Coruña), Patricia Simon Alonso (Complejo Hospitalario Universitario A Coruña), Marta Tejera Santana (Hospital Universitario de Gran Canaria Doctor Negrín), Elia Valls Pascual (Hospital Universitario Doctor Peset).

For peer review only

For peer review only - http://bmjopen.bmj.com/site/about/guidelines.xhtml

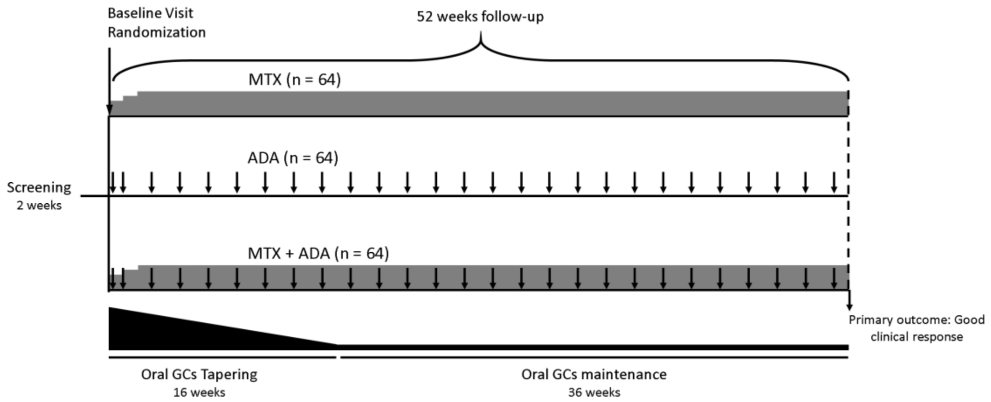

Overview of the study timeline and interventions

HOJA DE INFORMACIÓN AL PACIENTE

Solicitamos su participación en un proyecto de investigación promovido por investigadores de su centro y dirigido por el doctor Luis Rodriguez del Hospital Clínico San Carlos (Madrid). El estudio se titula **"Eficacia, seguridad y coste-efectividad del metotrexato, adalimumab, o su combinación en uveítis no anterior no infecciosa: un estudio multicéntrico, aleatorizado, paralelo de 3 brazos, con control activo, de fase 3, abierto, con evaluador cegado: Co-THEIA (CombinationTHerapy with mEthotrexate and adalImumAb for uveitis)"**

Siguiendo los estándares internacionalmente reconocidos y la normativa legal vigente en España sobre investigación, este ensayo clínico cumple todas las exigencias legales y ha sido aprobado por el Comité Ético de Investigación con medicamentos del Hospital Clínico San Carlos así como por la Agencia Española de Medicamentos y Productos Sanitarios.

Sin embargo, antes de aceptar participar o no, es importante que lea detenidamente la siguiente información y que realice todas las preguntas y aclare todas las cuestiones que crea conveniente con un familiar/amigo o con su médico.

¿Tengo que participar?

La participación en el estudio es completamente voluntaria. Rechazarla no acarreará ningún deterioro en la calidad de la asistencia ni en su atención médica. Además, podrá retirarse en cualquier momento del estudio sin tener que dar explicaciones y sin que ello repercuta en los

For peer review only - http://bmjopen.bmj.com/site/about/guidelines.xhtml

cuidados médicos que reciba. Su médico también podrá decidir, por alguna consideración médica o por cualquier otra razón, retirarle del estudio si así lo cree conveniente.

Así mismo, el hecho de que usted participe en este estudio no altera de ninguna manera ni sus tratamientos habituales ni el seguimiento de las enfermedades que tenga (en caso de que las tuviera).

¿En qué consiste el estudio y cómo se va a llevar a cabo?

Su médico le ha diagnosticado una uveítis no infecciosa, una inflamación de una parte del ojo que no está causada por un microorganismo (bacteria, hongo o virus).

Para tratar esta inflamación es posible que su oftalmólogo y/o reumatólogo/inmunólogo/especialista en Medicina Interna ya le haya pautado distintos tratamientos, principalmente corticoides. Sin embargo, estos fármacos a veces no son suficientes para controlar enfermedad. Por otro lado, es posible que haya sido diagnosticado/a de algún tipo de uveitis que se asocie a una inflamación más persistente. En ambos casos, en la actualidad disponemos de distintos medicamentos para el tratamiento pero solo tiene indicación aprobada el adalimumab. En este estudio vamos a comparar este medicamento y el metotrexato, solos o combinados.

El metotrexato es un fármaco que inhibe el sistema inmunitario y esto permite reducir la inflación del ojo. Se lleva utilizando desde hace más de 50 años en el tratamiento de otras enfermedades inflamatorias crónicas y más reccientemente (más de 30 años) en el tratamiento

For peer review only - http://bmjopen.bmj.com/site/about/guidelines.xhtml

de las uveitis. Se puede tomar en pastillas o inyectado en la piel (vía subcutánea), su médico según su caso le indicara como y cuando tomarlo.

El adalimumab es otro medicamente que también inhibe el sistema inmunitario y reduce la inflamación, pero utiliza otro mecanismo de acción. Se lleva utilizando en el tratamiento de las uveitis desde hace más de 10 años, administrándose por vía subcutánea.

Si usted decide participar en el estudio podrá ser asignado a uno de los tres grupos en estudio: metotrexato, adalimumab y ambos (metotrexato+adalimumab). La asignación será al azar, es decir ni usted ni su médico decidirá que tratamiento recibe. Indistintamente el grupo al que sea asignado, las visitas y las pruebas que se le realicen serán las que habitualmente se realizan a pacientes como usted. Además, en el caso de que haya sido diagnosticado de algunos subtipos de uveítis (como es la uveítis intermedia), se le realizará una resonancia magnética cerebral con gadolinio antes de iniciar el estudio. Esto es debido a que estos subtipos de uveitis podrían estar asociados a la presencia de enfermedades desmielinizantes del sistema nervioso central, en las que el uso de adalimumab se encuentra contraindicado.

For peer review only

Usted tendrá acudir cada 4 semanas al centro. En estas visitas su médico se encargará revisar la situación del ojo (examen del ojo, agudeza visual…), los eventos adversos y la adherencia al tratamiento. Además, deberá completar algunos cuestionarios para evaluar su calidad de vida, los costes y los fármacos que toma (aproximadamente 15 minutos). Para completar estos cuestionarios dispondrá de ayuda del personal del centro.

For peer review only - http://bmjopen.bmj.com/site/about/guidelines.xhtml

Adicionalmente le solicitaremos permiso para la extracción de unas muestras de sangre al inicio, tras 16 semanas, cuando finalice y en el caso de que tuviera algún brote de la uveítis durante el estudio. Estas muestras serán utilizadas para hacer análisis genéticos y de proteómica para identificar marcadores que permitan conocer más su enfermedad y /o que puedan estar asociados a una mejor respuesta al tratamiento. También, en el caso de que fuera a recibir adalimumab, para medir los niveles en sangre de ese fármaco. Por último, parte de estas muestras serán conservadas en la Colección de Muestras para la investigación en enfermedades reumatológicas del Servicio de Reumatología del Hospital Clínico San Carlos, con el objetivo de llevar a cabo futuros estudios que nos ayuden a conocer mejor la enfermedad que usted padece. Estas muestras solo serán conservadas y utilizadas en estudios futuros en caso de que usted de consentimiento informado expreso.

¿Qué beneficios puedo esperar por el hecho de participar en el estudio?

Puede ocurrir que usted no obtenga ningún beneficio personal de la participación en este proyecto de investigación. En cualquier caso, su participación en este estudio contribuirá al avance del conocimiento sobre el tratamiento de la uveítis no infecciosa.

¿Qué incomodidades o riesgos puede suponer mi participación en el estudio?

Entre los efectos secundarios más habituales del metotrexato están el dolor de cabeza, los moratones y sangrados, el aumento de las infecciones, las náuseas y vómitos, la diarrea, los dolores musculares, el cansancio, la pérdida de cabello y la afectación hepática.

For peer review only - http://bmjopen.bmj.com/site/about/guidelines.xhtml

Por su parte, el adalimumab presenta los siguientes efectos secundarios: molestias o dolor en el punto de inyección, erupción cutánea, dolor de cabeza e infecciones (siendo las más habituales las de las vías respiratorias altas (catarros entre otros) y la sinusitis).

En el caso de administrar de manera conjunta ambos fármacos los efectos adversos podrían ser más intensos y aparecer algunos desconocidos. En este momento, la administración de estos dos medicamentos de manera conjunta no esta autorizada.

En el caso de que se le tuviese que realizar una resonancia nuclear magnética con gadolinio, el uso de este contraste puede verse asociado a eventos adverso agudos en 1 de cada 1000 individuos, siendo los más frecuentes de la aparición de rash, urticaria y nauseas. Además, ha sido descrita la posibilidad de depósito de esta substancia a nivel cerebral y en otros tegidos, tras administraciones repetidas.

¿Cuáles son mis derechos y cómo van a ser tratados mis datos?

Toda la información que se registrará de usted será estrictamente confidencial. De acuerdo con el Reglamento General de Protección de Datos (RGPD) (Reglamento (EU) 2016/679), además de los derechos de acceso, rectificación, oposición y cancelación de datos (Ley Orgánica 3/2018, de 5 de diciembre, de Protección de Datos Personales y garantía de los derechos digitales), también tiene derecho a limitar el tratamiento de datos y solicitar una copia o que se trasladen a un tercero (portabilidad) los datos que usted ha facilitado para el estudio. Para ejercitar sus derechos, diríjase al investigador principal del estudio. No obstante, le recordamos que los datos no se pueden eliminar, aunque deje de participar en el ensayo para garantizar la validez de la investigación y cumplir con los deberes legales y los requisitos de autorización de medicamentos. Así mismo tiene derecho a dirigirse a la Agencia de Protección de Datos si no quedara satisfecho.

For peer review only - http://bmjopen.bmj.com/site/about/guidelines.xhtml

For peer review only

En todo momento se mantendrá el anonimato de los pacientes y, para ello, en ninguno de los documentos del estudio aparecerá su nombre, sino que le será asignado un número que será el que se utilice en todos ellos. En todos los informes escritos y publicaciones, sólo aparecerá su número de referencia. Sólo el médico responsable del estudio guardará, en condiciones de seguridad, la lista que relaciona los nombres de los pacientes con los números de referencia asignados a cada uno.

Sólo tendrán acceso a los datos del estudio el equipo investigador y el monitor del estudio, miembros del comité, autoridades sanitarias competentes y/o de la agencia reguladora (AEMPS) para asegurar que el estudio se está llevando a cabo con las leyes vigentes y la reglamentación sanitaria. Firmando este documento, usted está autorizando este acceso. Además, los resultados del estudio siempre serán presentados de manera global y nunca de forma individualizada.

El Investigador y el Promotor están obligados a conservar los datos recogidos para el estudio al menos hasta 25 años tras su finalización. Posteriormente, su información personal solo se conservará por el centro para el cuidado de su salud y por el promotor para otros fines de investigación científica si usted hubiera otorgado su consentimiento para ello, y si así lo permite la ley y requisitos éticos aplicables.

Si realizáramos transferencia de sus datos codificados fuera de la UE a las entidades de nuestro grupo, a prestadores de servicios o a investigadores científicos que colaboren con nosotros, los

For peer review only - http://bmjopen.bmj.com/site/about/guidelines.xhtml

datos del participante quedarán protegidos con salvaguardas tales como contratos u otros mecanismos por las autoridades de protección de datos. Si el participante quiere saber más al respecto, puede contactar al Delegado de Protección de Datos del promotor o Investigador Principal.

¿Mi participación supondrá algún coste o compensación económica?

Su participación en este estudio no supondrá para usted ningún coste económico, así como tampoco será recompensado económicamente por ello.

estudio dispone de una póliza de seguros que se ajusta a la legislación vigente (Real decreto 1090/2015) y que le proporcionará la compensación e indemnización en caso de menoscabo de su salud o de lesiones que pudieran producirse en relación con su participación en el estudio, siempre que no sean consecuencia de la propia enfermedad que se estudia o de la evolución propia de su enfermedad como consecuencia de la ineficacia del tratamiento. Este estudio se considera de Bajo Nivel de Intervención (estudios de bajo riesgo, muy parecidos a la práctica clinica habitual), por lo que la póliza con la que esta usted cubierto es la póliza de Sistema de Salud.

Si usted tiene alguna duda o quiere más información, no dude en consultar con el médico responsable que le está solicitando este consentimiento.

For peer review only - http://bmjopen.bmj.com/site/about/guidelines.xhtml

¿Cómo obtener información adicional?

Si desea información adicional sobre el estudio puede contactar con:

____

GRACIAS POR LEER ESTA INFORMACIÓN

For peer review only

For peer review only - http://bmjopen.bmj.com/site/about/guidelines.xhtml

CONSENTIMIENTO INFORMADO PARA LOS PACIENTES

Título del estudio: **"Eficacia, seguridad y coste-efectividad del metotrexato, adalimumab, o su combinación en uveítis no anterior no infecciosa: un estudio multicéntrico, aleatorizado, paralelo de 3 brazos, con control activo, de fase 3, abierto, con evaluador cegado: Co-THEIA (CombinationTHerapy with mEthotrexate and adalImumAb for uveitis)"**

Yo…………………………………………………………………………..

(Nombre y apellidos del participante, puño y letra del paciente)

DECLARO:

- Que he hablado con ……………………………………………….

(Nombre del médico, puño y letra del paciente)

- Que he leído la hoja de información que se me ha entregado sobre el estudio.
- He comprendido la información recibida y he podido formular todas las preguntas que he creído oportunas.
- Comprendo que mi participación es voluntaria y que en cualquier momento puedo revocar mi consentimiento sin tener que dar explicaciones y sin que afecte en ningún aspecto a mi relación con el personal médico ni a la atención recibida por su parte.
- He recibido una copia firmada y fechada de este Consentimiento Informado.

☐ **Autorizo la conservación de muestras biológicas obtenidas y su uso para estudios futuros**

EN CONSECUENCIA, DOY MI CONSENTIMIENTO PARA FORMAR PARTE DE ESTE PROYECTO DE INVESTIGACION.

Firma del paciente Firma del médico responsable

…………………………………… ………………………………….

Nombre y apellidos Nombre y apellidos

Fecha………………………….. Fecha…………………………..

For peer review only - http://bmjopen.bmj.com/site/about/guidelines.xhtml

Reporting checklist for protocol of a clinical trial.

Based on the SPIRIT guidelines.

Instructions to authors

Complete this checklist by entering the page numbers from your manuscript where readers will find each of the items listed below.

Your article may not currently address all the items on the checklist. Please modify your text to include the missing information. If you are certain that an item does not apply, please write "n/a" and provide a short explanation.

Upload your completed checklist as an extra file when you submit to a journal.

In your methods section, say that you used the SPIRITreporting guidelines, and cite them as:

Chan A-W, Tetzlaff JM, Gøtzsche PC, Altman DG, Mann H, Berlin J, Dickersin K, Hróbjartsson A, Schulz KF, Parulekar WR, Krleža-Jerić K, Laupacis A, Moher D. SPIRIT 2013 Explanation and Elaboration: Guidance for protocols of clinical trials. BMJ. 2013;346:e7586

		Reporting Item	Page Number
**Administrative information**			
Title	#1	Descriptive title identifying the study design, population, interventions, and, if applicable, trial acronym	1
Trial registration	#2a	Trial identifier and registry name. If not yet registered, name of intended registry	21
Trial registration: data set	#2b	All items from the World Health Organization Trial Registration Data Set	22
Protocol version	#3	Date and version identifier	21
Funding	#4	Sources and types of financial, material, and other support	32
Roles and responsibilities: contributorship	#5a	Names, affiliations, and roles of protocol contributors	32

For peer review only

Roles and responsibilities: sponsor contact information	#5b	Name and contact information for the trial sponsor	33
Roles and responsibilities: sponsor and funder	#5c	Role of study sponsor and funders, if any, in study design; collection, management, analysis, and interpretation of data; writing of the report; and the decision to submit the report for publication, including whether they will have ultimate authority over any of these activities	33
Roles and responsibilities: committees	#5d	Composition, roles, and responsibilities of the coordinating centre, steering committee, endpoint adjudication committee, data management team, and other individuals or groups overseeing the trial, if applicable (see Item 21a for data monitoring committee)	33
**Introduction**			
Background and rationale	#6a	Description of research question and justification for undertaking the trial, including summary of relevant studies (published and unpublished) examining benefits and harms for each intervention	7
Background and rationale: choice of comparators	#6b	Explanation for choice of comparators	6
Objectives	#7	Specific objectives or hypotheses	9
Trial design	#8	Description of trial design including type of trial (eg, parallel group, crossover, factorial, single group), allocation ratio, and framework (eg, superiority, equivalence, non-inferiority, exploratory)	9
**Methods: Participants, interventions, and outcomes**			
Study setting	#9	Description of study settings (eg, community clinic, academic hospital) and list of countries where data will be collected. Reference to where list of study sites can be obtained	11
Eligibility criteria	#10	Inclusion and exclusion criteria for participants. If applicable, eligibility criteria for study centres and individuals who will	11

For peer review only

		perform the interventions (eg, surgeons, psychotherapists)	
Interventions: description	#11a	Interventions for each group with sufficient detail to allow replication, including how and when they will be administered	12
Interventions: modifications	#11b	Criteria for discontinuing or modifying allocated interventions for a given trial participant (eg, drug dose change in response to harms, participant request, or improving / worsening disease)	15
Interventions: adherance	#11c	Strategies to improve adherence to intervention protocols, and any procedures for monitoring adherence (eg, drug tablet return; laboratory tests)	17
Interventions: concomitant care	#11d	Relevant concomitant care and interventions that are permitted or prohibited during the trial	12
Outcomes	#12	Primary, secondary, and other outcomes, including the specific measurement variable (eg, systolic blood pressure), analysis metric (eg, change from baseline, final value, time to event), method of aggregation (eg, median, proportion), and time point for each outcome. Explanation of the clinical relevance of chosen efficacy and harm outcomes is strongly recommended	9
Participant timeline	#13	Time schedule of enrolment, interventions (including any run-ins and washouts), assessments, and visits for participants. A schematic diagram is highly recommended (see Figure)	15
Sample size	#14	Estimated number of participants needed to achieve study objectives and how it was determined, including clinical and statistical assumptions supporting any sample size calculations	17
Recruitment	#15	Strategies for achieving adequate participant enrolment to reach target sample size	11
**Methods: Assignment of interventions (for controlled trials)**			
Allocation: sequence generation	#16a	Method of generating the allocation sequence (eg, computer-generated random numbers), and list of any factors for stratification. To reduce predictability of a random sequence, details of any planned restriction (eg, blocking) should be provided in a separate document that is unavailable to those who enrol participants or assign interventions	12

For peer review only

Allocation concealment mechanism	#16b	Mechanism of implementing the allocation sequence (eg, central telephone; sequentially numbered, opaque, sealed envelopes), describing any steps to conceal the sequence until interventions are assigned	12
Allocation: implementation	#16c	Who will generate the allocation sequence, who will enrol participants, and who will assign participants to interventions	12
Blinding (masking)	#17a	Who will be blinded after assignment to interventions (eg, trial participants, care providers, outcome assessors, data analysts), and how	13
Blinding (masking): emergency unblinding	#17b	If blinded, circumstances under which unblinding is permissible, and procedure for revealing a participant's allocated intervention during the trial	13
**Methods: Data collection, management, and analysis**			
Data collection plan	#18a	Plans for assessment and collection of outcome, baseline, and other trial data, including any related processes to promote data quality (eg, duplicate measurements, training of assessors) and a description of study instruments (eg, questionnaires, laboratory tests) along with their reliability and validity, if known. Reference to where data collection forms can be found, if not in the protocol	10
Data collection plan: retention	#18b	Plans to promote participant retention and complete follow-up, including list of any outcome data to be collected for participants who discontinue or deviate from intervention protocols	11
Data management	#19	Plans for data entry, coding, security, and storage, including any related processes to promote data quality (eg, double data entry; range checks for data values). Reference to where details of data management procedures can be found, if not in the protocol	15
Statistics: outcomes	#20a	Statistical methods for analysing primary and secondary outcomes. Reference to where other details of the statistical analysis plan can be found, if not in the protocol	18
Statistics: additional analyses	#20b	Methods for any additional analyses (eg, subgroup and adjusted analyses)	18

For peer review only

Statistics: analysis population and missing data	#20c	Definition of analysis population relating to protocol non-adherence (eg, as randomised analysis), and any statistical methods to handle missing data (eg, multiple imputation)	17
**Methods: Monitoring**			
Data monitoring: formal committee	#21a	Composition of data monitoring committee (DMC); summary of its role and reporting structure; statement of whether it is independent from the sponsor and competing interests; and reference to where further details about its charter can be found, if not in the protocol. Alternatively, an explanation of why a DMC is not needed	16
Data monitoring: interim analysis	#21b	Description of any interim analyses and stopping guidelines, including who will have access to these interim results and make the final decision to terminate the trial	18
Harms	#22	Plans for collecting, assessing, reporting, and managing solicited and spontaneously reported adverse events and other unintended effects of trial interventions or trial conduct	15
Auditing	#23	Frequency and procedures for auditing trial conduct, if any, and whether the process will be independent from investigators and the sponsor	15
**Ethics and dissemination**			
Research ethics approval	#24	Plans for seeking research ethics committee / institutional review board (REC / IRB) approval	21
Protocol amendments	#25	Plans for communicating important protocol modifications (eg, changes to eligibility criteria, outcomes, analyses) to relevant parties (eg, investigators, REC / IRBs, trial participants, trial registries, journals, regulators)	21
Consent or assent	#26a	Who will obtain informed consent or assent from potential trial participants or authorised surrogates, and how (see Item 32)	21
Consent or assent: ancillary studies	#26b	Additional consent provisions for collection and use of participant data and biological specimens in ancillary studies, if applicable	22
Confidentiality	#27	How personal information about potential and enrolled participants will be collected, shared, and maintained in order to protect confidentiality before, during, and after the trial	22

For peer review only

Declaration of interests	#28	Financial and other competing interests for principal investigators for the overall trial and each study site	32
Data access	#29	Statement of who will have access to the final trial dataset, and disclosure of contractual agreements that limit such access for investigators	33
Ancillary and post trial care	#30	Provisions, if any, for ancillary and post-trial care, and for compensation to those who suffer harm from trial participation	15
Dissemination policy: trial results	#31a	Plans for investigators and sponsor to communicate trial results to participants, healthcare professionals, the public, and other relevant groups (eg, via publication, reporting in results databases, or other data sharing arrangements), including any publication restrictions	22
Dissemination policy: authorship	#31b	Authorship eligibility guidelines and any intended use of professional writers	32
Dissemination policy: reproducible research	#31c	Plans, if any, for granting public access to the full protocol, participant-level dataset, and statistical code	22
**Appendices**			
Informed consent materials	#32	Model consent form and other related documentation given to participants and authorised surrogates	14
Biological specimens	#33	Plans for collection, laboratory evaluation, and storage of biological specimens for genetic or molecular analysis in the current trial and for future use in ancillary studies, if applicable	10

The SPIRIT Explanation and Elaboration paper is distributed under the terms of the Creative Commons Attribution License CC-BY-NC. This checklist was completed on 16. March 2021 using https://www.goodreports.org/, a tool made by the EQUATOR Network in collaboration with Penelope.ai

For peer review only

BMJ Open

Efficacy, safety and cost-effectiveness of methotrexate, adalimumab, or their combination in non-infectious non-anterior uveitis: a protocol for a multicenter, randomized, parallel 3 arms, active-controlled, phase 3 open label with blinded outcome assessment study

Journal:	*BMJ Open*
Manuscript ID	bmjopen-2021-051378.R1
Article Type:	Protocol
Date Submitted by the Author:	14-Nov-2021

Complete List of Authors:	Rivas, Ana; La Fundacion para la Investigacion Biomedica del Hospital Clinico San Carlos<br>Lopez-Picado, Amanda; La Fundacion para la Investigacion Biomedica del Hospital Clinico San Carlos<br>Calamia, Valentina; Instituto de Investigación Biomédica de A Coruña<br>Carreño, Ester; Hospital Universitario Fundacion Jimenez Diaz<br>Cocho, Lidia; Institute of Applied Ophtalmobiology<br>Cordero-Coma, Miguel; University Hospital Centre León<br>Fonollosa, Alex; Cruces University Hospital<br>Francisco Hernandez, Felix; Hospital Universitario de Gran Canaria Dr Negrin<br>Garcia-Aparicio, Angel; Hospital Universitario de Toledo<br>Garcia-Gonzalez, Javier; Hospital Universitario 12 de Octubre<br>Mondejar, Jose Juan; Alicante General University Hospital<br>Lojo-Oliveira, Leticia; Hospital Universitario Infanta Leonor<br>Martínez-Costa, Llucí; Hospital Universitario Doctor Peset, Ophthalmology<br>Munoz, Santiago; Hospital Universitario Infanta Sofia<br>Peiteado, Diana; Hospital Universitario La Paz<br>Pinto, Jose Antonio; Complexo Hospitalario Universitario A Coruña Servizo de Pediatría<br>Rodriguez-Lozano, Beatriz; Hospital Universitario de Canarias<br>Pato, Esperanza; Hospital Clínico San Carlos<br>Diaz-Valle, David; Hospital Clínico San Carlos<br>Molina, Elena; La Fundacion para la Investigacion Biomedica del Hospital Clinico San Carlos<br>Tebar, Luis; La Fundacion para la Investigacion Biomedica del Hospital Clinico San Carlos<br>Rodriguez-Rodriguez, Luis; La Fundacion para la Investigacion Biomedica del Hospital Clinico San Carlos,

**Primary Subject Heading**:	Ophthalmology
Secondary Subject Heading:	Pharmacology and therapeutics
Keywords:	Clinical trials < THERAPEUTICS, OPHTHALMOLOGY, RHEUMATOLOGY

Efficacy, safety and cost-effectiveness of methotrexate, adalimumab, or their combination in non-infectious non-anterior uveitis: a protocol for a multicenter, randomized, parallel 3 arms, active-controlled, phase 3 open label with blinded outcome assessment study

Ana Rivas[1,2], Amanda López Picado[1], Valentina Calamia[3], Ester Carreño[4], Lidia Cocho[5], Miguel Cordero-Coma[6], Alex Fonollosa[7], Felix M Francisco Hernandez[8], Angel Garcia Aparicio[9], Javier Garcia Gonzalez[10], Jose Juan Mondejar[11], Leticia Lojo Oliveira[12], Llucí Martinez-Costa[13], Santiago Muñoz[14], Diana Peiteado[15], Jose Antonio Pinto[16], Beatriz Rodriguez Lozano[17], Esperanza Pato[18], David Diaz-Valle[19], Elena Molina[20], Luis Tebar[1], Luis Rodriguez-Rodriguez[21], CoTHEIA Study Group.

[1] Unidad de Investigación Clinica y Ensayos Clínicos. Hospital Clínico San Carlos,, IdISSC, Madrid (Spain), [2] Departamento de Enfermería. Facultad Enfermería, Fisioterapia y Podología. Universidad Complutense de Madrid, Madrid (Spain), [3]Unidad de Proteómica. Grupo de Investigación de Reumatología (GIR). Instituto de Investigación Biomédica de A Coruña (INIBIC), Complexo Hospitalario Universitario de A Coruña (CHUAC), and Universidade da Coruña (UDC), A Coruña (Spain), [4]University Hospital Fundacion Jimenez Diaz, and University Hospital Rey Juan Carlos, Madrid (Spain), [5]IOBA (Institute of Applied OphthalmoBiology), University of Valladolid, and Hospital Clínico Universitario de Valladolid, Valladolid (Spain), [6]Uveitis Unit. University Hospital of León, IBIOMED, and University of León, Leon (Spain), [7]Department of Ophthalmology, BioCruces Bizkaia Health Research Institute, Cruces University Hospital, University of the Basque Country, Barakaldo, Spain, [8]Rheumatology Department, Hospital Universitario de Gran Canaria Doctor Negrín, Las

For peer review only - http://bmjopen.bmj.com/site/about/guidelines.xhtml

Palmas de Gran Canaria (Spain), [9]Department of Rheumatology, Hospital Universitario de Toledo, Toledo (Spain), [10]Department of Rheumatology, Hospital Universitario 12 de Octubre, Madrid (Spain), [11]Ophthalmology Department, Hospital General Universitario de Alicante, Alicante (Spain), [12]Sección de Reumatología, Hospital Universitario Infanta Leonor, Madrid (Spain), [13]Ophthalmology Department, Hospital Universitario Doctor Peset, Valencia (Spain), [14]Rheumatology Department, Hospital Universitario Infanta Sofia, San Sebastián de los Reyes (Spain), [15]Rheumatology Unit. Hospital Universitario La Paz, Madrid (Spain), [16]Rheumatology Department, Complexo Hospitalario Universitario de A Coruña (CHUAC), A Coruña (Spain), [17]Rheumatology Department, Hospital Universitario de Canarias, Santa Cruz de Tenerife (Spain), [18]Rheumatology Department, Hospital Clínico San Carlos, IdISSC, Madrid (Spain), [19]Servicio de Oftalmología, Hospital Clínico San Carlos. Departamento de Inmunología, Oftalmología y ORL, Facultad de Medicina, Universidad Complutense de Madrid, IdISSC, Madrid (Spain), [20]Department of Pathology, Hospital Clínico San Carlos, IdISSC, Madrid (Spain), [21]Musculoskeletal Pathology Group, Rheumatology Department, Hospital Clínico San Carlos, IdISSC, Madrid (Spain)

Correspondence

Dr Luis Rodriguez-Rodriguez, Hospital Clínico San Carlos, c\ Prof. Martín Lagos s/n, 28040, Madrid (Spain); Phone: +34913303615; email:lrrodriguez@salud.madrid.org

Word Count: 4,678

For peer review only

For peer review only - http://bmjopen.bmj.com/site/about/guidelines.xhtml

ABSTRACT

Introduction: Non-infectious uveitis (NIUs) include a heterogeneous group of sight-threatening and incapacitating conditions.. Their correct management sometimes requires the use of immunosuppressive drugs (ISDs), prescribed in monotherapy or in combination. Several observational studies showed that the use of ISDs in combination could be more effective than and as safe as their use in monotherapy. However, a direct comparison between these two treatment strategies has not been carried out yet.

Methods and analysis: The Combination THerapy with mEthotrexate and adalImumAb for uveitis (CoTHEIA) study is a phase III, multicenter, prospective, randomized, single-blinded with masked outcome assessment, parallel three arms with 1:1:1 allocation, active-controlled, superiority study design, comparing the efficacy, safety and cost-effectiveness of methotrexate (MTX), adalimumab (ADA), or their combination in non-infectious non-anterior uveitis. We aim to recruit 192 subjects. The duration of the treatment and follow-up will last up to 52 weeks, plus 70 days follow-up with no treatment. The complete and maintained resolution of the ocular inflammation will be assessed by masked evaluators (primary outcome). In addition to other secondary measurements of efficacy (QoL, visual acuity, costs) and safety, we will identify subjects' subgroups with different treatment responses by developing prediction models based on machine learning techniques using genetic and proteomic biomarkers.

Ethics and dissemination: The protocol, annexes and informed consent forms were approved by the Reference Clinical Research Ethic Committee at the Hospital Clínico San Carlos (Madrid, Spain) and the Spanish Agency for Medicines and Health Products (AEMPS). We will elaborate a Dissemination plan including production of materials adapted to several formats to communicate the clinical trial progress and findings to a

For peer review only - http://bmjopen.bmj.com/site/about/guidelines.xhtml

broad group of stakeholders. The promoter will be the only with access to the participant-level data, although it can be shared within the legal situation.

Registration details: clinicaltrialsregister.eu (2020-000130-18) and clinicaltrials.gov (NCT04798755).

Keywords: Uveitis, adalimumab, methotrexate, combination therapy, randomised clinical trial

For peer review only

For peer review only - http://bmjopen.bmj.com/site/about/guidelines.xhtml

ARTICLE SUMMARY

Strengths and Limitations of this Study

1. This is the first randomized controlled study designed to compare the efficacy of combination therapy versus monotherapy for the treatment of non-infectious uveitis in subjects with no previous immunosuppressive treatment.

2. We have chosen quite a strict outcome (the requirement of a maintained controlled inflammation), more likely related to long-term outcomes (such as structural damage), and to patient reported outcomes measures.

3. Despite the previous point, our primary efficacy outcome is still a surrogate marker: the achievement of this outcome does not have necessarily to translate in an improvement of outcomes more important for the patient, such as quality of life or disability.

4. The requirement to control the inflammatory process early (by week 16) may cause an underestimation of drug efficacy, as it could take more time to control the inflammation but be associated with a similar long-term prognosis.

5.The lack of masking could introduce bias, although treatment characteristics and proven effectivity in non-infectious uveitis, the duration of the trial (up to 52 weeks), and the need for bi-weekly subcutaneous injections of one of the drugs (ADA), we consider unpractical for the subject the use of placebo in the present trial.

For peer review only - http://bmjopen.bmj.com/site/about/guidelines.xhtml

INTRODUCTION

Uveitides are potentially sight-threatening diseases[1]:worldwide, they represented up to 10% of causes of blindness (almost 4 million people)[2]. Furthermore, in the European Union and the United States, after diabetic retinopathy, uveitides represent the second major treatable cause of blindness in those 20-65 years of age[3](up to 10% of cases of blind registrations[3–6]). Additionally, a high percentage of patients suffer from uveitis-related complications, visual impairment[4,7,8], and a negative impact in quality-of-life (QoL)[9,10]. Considering their higher prevalence in young to middle-aged adults[11,12] uveitides cause an important economic, social, and personal burden[5,13–16].

The correct management of NIUs is essential for preserving visual function and avoiding ocular and extra-ocular morbidity[17]. Although glucocorticoids (GCs) are the mainstay of treatment[18], under certain circumstances, adding immunosuppressive drugs (ISDs) is needed to achieve a sustained control of the inflammatory process[19]. Several ISDs are used in the standard of care, such as methotrexate (MTX), and biological agents[19–21].

Regarding MTX, its effectiveness in NIU has been assessed in two randomized clinical trials, compared with mycophenolate mofetil (MMF): Rathinam et al[22] observed that 69% and 47% of patients treated with MTX and MMF, respectively, achieved complete control of inflammation and daily oral GCs dosage ≤10mg at 5 and 6 months ($p=0.09$). In a second trial[23], randomizing 200 patients, the percentage of subjects achieving a similar outcome was 67% and 57% for MTX and MMF, respectively ($p>0.05$), with similar tolerability. Regarding safety, adverse events (AEs) are generally mild and

For peer review only - http://bmjopen.bmj.com/site/about/guidelines.xhtml

discontinuations due to serious adverse events (SAEs) are less common than for most ISDs[24][25], translating it in higher retention rates[26,27].

Regarding ADA, its effectiveness in NIU has been shown in two randomized controlled trials[28,29]. The VISUAL I study[28] compared ADA with placebo in 217 NIU patients with active uveitis despite ≥10 mg/day of systemic GCs. Based on the cumulative number of subjects with treatment failure in each visit, by week 25 (about 6 months), 31 of 107 (29%) subjects in the placebo group and 62 of 110 (56%) subjects in the ADA group had not suffered a treatment failure. By 50 weeks, the numbers were reduced to 24 (22%) and 51 (46%), respectively. Regarding safety, many of the AEs are sufficiently mild to not require discontinuation, such as injection site pain, and anti-drug antibodies formation[19,30,31], reflecting in a high retention rate[32]. Regarding SAEs, there was no association with higher risk in a recent meta-analysis[33], compared with placebo or synthetic ISDs. However, ADA was associated to a higher risk of treatment discontinuation due to SAEs. One of the most important ADA's AEs are infections. However, a previous meta-analysis reported that the absolute risk was low (0.036% with TNF-alpha inhibitors vs. 0.017% with placebo)[34], which probably does not represent a clinically important constraint on the use of these agents. Finally, ADA has not showed an association with a higher risk of malignancy[33].

Although ISDs are usually used in monotherapy in uveitis, several observational studies have shown that in 21 to 52% percentage of NIU patients, the use of ISDs in monotherapy was unable to achieve a sustained control of the inflammatory process[27,35–37].Furthermore, other studies have provided evidence that the combination of 2 or more ISDs could offer advantages in terms of effectiveness and tolerability[38–41], in conditions such as Birdshot retinochoroidopathy[38], serpiginous

For peer review only

choroiditis[39,40], Vogt-Koyanagi-Harada syndrome (VHK)[42,43], ocular Behçet's disease[44,45], JIA associated uveitis[46], sympathetic ophthalmia[47] and intermediate uveitis[48]. Regarding the beneficial of combining both MTX and ADA, several RCTs have provided evidence in other immune mediated inflammatory diseases (IMIDs)[49]. In NIU, this combinations was tested in children with Juvenile Idiopathic Arthritis-associated Uveitis and a previous failure to MTX monotherapy [50]. The combination of MTX and ADA was more effective compared to MTX and placebo, although it was associated with a higher proportion of AEs (88% vs. 83% of patients) and SAEs (22% vs. 7%).

Despite all the evidence, a direct comparison between combination and monotherapy has not been tested yet, although some groups have adopted the use of combination therapy as the initial ISD treatment for particular conditions[51].

There are currently no tools able to predict the response or non-response to ISDs in those NIUs needing immunosuppression, as there is marked interpersonal variation in their efficacy and toxicity. Response to the first-line ISD treatment could be an important predictor of long-term outcomes, as the continuous or repeated eye inflammation could increase the risk of structural permanent damage, leading to blindness, disability, and deterioration in the QoL. Therefore, starting on the right ISD is likely a key factor in achieving a better and more cost-effective therapy, and improving the optimal allocation of health care resources. To achieve these aims, objectively measured and evaluated characteristics (biomarkers[52]) are required, in addition to the identification of clinical features associated with the outcomes of interest.

For peer review only - http://bmjopen.bmj.com/site/about/guidelines.xhtml

METHODS AND ANALYSIS

Study overview

The Combination THerapy with mEthotrexate and adalImumAb for uveitis (CoTHEIA) study is a phase III, multicenter, prospective, randomized, single-blinded with masked outcome assessment, parallel 3 arms with 1:1:1 allocation, active-controlled, superiority study design, comparing the efficacy, safety and cost-effectiveness of MTX, ADA, or their combination in non-infectious non-anterior uveitis (NINAU). The duration of the treatment and follow-up will last up to 52 weeks. A 70-day follow-up clinic visit or phone call will take place to assess safety after the last study drug dose. **Figure 1** provides an overview of the study timeline and interventions.

Objectives and Outcome Variables

The trial main hypothesis is that the use of combination therapy with MTX and ADA will be more effective in inducing and maintaining ocular inflammatory inactivity than either drug given in monotherapy.

The *primary efficacy objective* is to establish which treatment strategy results in a higher proportion of subjects achieving a complete and maintained resolution of the ocular inflammation, on an intent to treat basis. The *primary efficacy outcome* will be the proportion of patients achieving a **Good Clinical Response** between the combination therapy arm and the single immunosuppressive drugs arms. This outcome is defined as a complete resolution of the ocular inflammatory signs (including active chorioretinal lesions, active retinal vascular inflammation, uveitic macular oedema, presence of anterior chamber cells, and presence of vitreous haze), achieved within the

For peer review only - http://bmjopen.bmj.com/site/about/guidelines.xhtml

first 16 weeks of the study, and maintained during follow-up until the end of the study (week 52); furthermore, there must not be a treatment failure due to safety or intolerability; the subject must adhere to the initial (up to week 16) oral GCs tapering protocol; all study visits from Baseline to 16 weeks must be completed; and at the final visit (week 52), the subject must be treated with up to 7.5mg/day of oral prednisone (or equivalent) and up to 2 times a day of prednisolone acetate 1% (or equivalent).The *secondary efficacy objectives* and outcomes variables can be found at **Table 1**.

Table 1: Efficacy-related secondary objectives and outcome variables.

Objective	Outcome variable
To compare the fraction of subjects who achieve a complete inflammatory ocular inactivity by week 16	Complete abrogation of the ocular inflammatory signs, which is achieved within the first 16 weeks of the study, no treatment failure due to safety or intolerability; compliance with the initial (up to week 16) oral GCs tapering protocol, and completion of all study visits from Baseline to 16 weeks
To compare several Patient Reported Outcomes Measures (health- and vision-related quality of life, anxiety and depression) between treatment strategies	EuroQuol 5D-5L<br>Visual Functioning Questionnaire-25<br>Hospital Anxiety and Depression Scale
To compare the presence of the clinical components of the Good Clinical Response variable between treatment strategies during follow-up	Active chorioretinal lesions; active retinal vascular inflammation; macular edema; ACC; vitreous haze; and loss of CVA secondary to inflammation at baseline, week 16, week 52, and relapse visit
To compare the time to relapse after week 16 between treatment strategies	The time to inflammatory relapse between groups, defined as the time from visit 16 weeks until end of the study, loss of follow-up or appearance of at least one ocular inflammatory manifestation, in those individuals achieving a Good Clinical Response by visit 16 week
To compare the evolution of visual acuity during follow-up	Best corrected visual acuity (BCVA) during follow-up
To compare the development of anti-adalimumab antibodies during the follow-up, between those subjects treated with monotherapy and combination therapy	Assessment of anti-ADA antibodies (AAA) at week 15, 27, 51, and relapse.

For peer review only - http://bmjopen.bmj.com/site/about/guidelines.xhtml

To assess the cost-utility and cost-effectiveness from both a Health System and a Societal perspective of the combination therapy and the ADA monotherapy compared with MTX given alone	Direct and indirect cost, and Incremental Cost Effectiveness Ratios

Security-related objective will establish which treatment strategy has better tolerability. Safety outcomes will be collected in the form of AEs, physical examination, and laboratory tests throughout the treatment period and up to 70 days after the last dose in this study. Gender of the subjects will be taken into account[53].

Pharmacogenetic and Proteomic-related objectives include identifying groups of subjects more likely to respond to the different treatment strategies, using genetic and proteomic biomarkers. For the former, subjects will be genotyped for known and validated genetics single nucleotide polymorphisms (SNPs) associated with a MTX[54] and ADA[55] response in different IMIDs. For the latter, a Discovery (shotgun proteomic analysis), Verification (Targeted proteomics using Multiple reaction monitoring test) and Validation (antibody-based microarrays absolute quantification tests) phases will be carried out, following optimized protocols and procedures[56–61].

Finally, a *Biobank substudy-related objective* will be carried out with the aim of boosting the translational research in the field of NIUs, by creating a collection of blood-derived samples (serum, plasma, total blood RNA, and DNA) from the participant subjects in order to advance in the identification and validation of biomarkers associated with treatment response or deepen in the pathophysiology of these conditions.

Settings and eligibility

For peer review only - http://bmjopen.bmj.com/site/about/guidelines.xhtml

The study population encompasses subjects diagnosed with non-infectious intermediate, posterior, or panuveitis with active disease within 180 days before the start of the study (Baseline visit), and either a documented failure to systemic or local GCs, or a chronic disease requiring GC-sparing ISD treatment. Main inclusion and exclusion criteria can be found at **Table 2**.

Table 2: Study participant inclusion and exclusion criteria

Inclusion criteria	Exclusion criteria
Adult subjects (≥18 years old);	Subjects with ocular histoplasmosis syndrome, or ocular masquerade syndromes;
Diagnosed with non-infectious intermediate, posterior, or panuveitis in at least one eye;	Evidence or history of malignancy;
Who have active disease within the previous 180 days before Baseline, and either	Corneal, lens or vitreous opacities precluding the visualization of the fundus;
A documented failure to systemic or local GCs in the previous 6 months, OR	Uncontrolled intraocular pressure;
A chronic disease necessitating GC-sparing immunosuppressive treatment (such as Behcet's disease with posterior segment involvement, multifocal choroiditis with panuveitis, serpiginous choroidopathy, birdshot retinochoroidopathy, diffuse retinal vasculitis, Vogt-Koyanagi-Harada with bullous serous retinal and/or choroidal detachments, or sympathetic ophthalmia;	Best corrected visual acuity (CVA) <20/400;
Able and willing to self-administer subcutaneous (SC) injections or have a qualified person available to administer SC injections; and	History, symptoms and/or Magnetic Resonance Imaging (MRI) findings suggestive of a demyelinating disease;
A negative PPD test (or equivalent) and a chest x-ray (CXR) at Screening OR if positive PPD test (or equivalent) and/or a CXR consistent with prior tuberculosis (TB) exposure, the subject must initiate, be currently receiving or have documented completion of a course of TB prophylaxis therapy, according to clinical practice.	History of moderate to severe congestive heart failure (NYHA class III or IV)
	Previous exposure to TNFi therapies;
	Previous exposure to synthetic ISDs in the previous 6 months before Baseline;
	Prior intolerability, safety issues, or ineffectiveness of MTX and/or ADA;
	Use of GCs implants (Iluvien® within 3 years, Ozurdex® within 6 months before Baseline);

For peer review only

	Use of intraocular or periocular GCs injection within 90 days before Baseline;
	Ocular surgery within 30 days before Baseline;
	Planned (elective) eye surgery in the following 52 weeks from Baseline;
	Proliferative or severe non-proliferative diabetic retinopathy;
	Neovascular/wet age-related macular degeneration;
	Chronic structural damage considered by the Investigator to interfere with measurement of macular thickness, impede the potential for its normalization, or can cause damage independent of the inflammatory process;
	Systemic inflammatory disease considered by the Investigator as likely to require high GCs dosage or prohibited medications;
	Presence of chronic recurring infections (HBV, syphilis), active TB and/or a history of invasive infection;
	Positive pregnancy test;
	Breast-feeding or considering becoming pregnant during the study.

Subjects will be recruited from 16 Spanish academic hospitals located in different regions of Spain (Galicia, País Vasco, Castilla y León, Comunidad de Madrid, Castilla La Mancha, Comunidad Valenciana, and Comunidad Canaria). In addition, the Instituto de Investigación Biomédica de A Coruña (INIBIC) will carry out the proteomic analysis, and the Instituto de Investigación Sanitaria San Carlos (IdISSC) Musculoskeletal Pathology Group will be responsible for the pharmacogenetic analysis and development of prediction models for drug response.

For peer review only

Recruitment

It will extend over 18 months. The participating study sites attends several tens of new patients every year, with several hundreds or thousands being followed-up, and many of these sites act as reference centres for other secondary and tertiary hospitals. Candidate subjects will be identified from patients lists AND/OR research databases AND among the new patients attended at the sites.

Subject will not receive any financial compensation for participating in the study.

To ensure subject's retention during follow-up, we will request contact details, carry out reminder phone calls/send emails, review barriers to attend appointments, and educate them in the significance of research follow-up even if they decide to discontinue the study drugs.

Intervention

All subjects entering the study will be centrally randomized at the Baseline visit into one of three study arms, in a 1:1:1 ratio:

Arm 1 will receive at the Baseline visit ADA 80 mg SC loading dose followed a week later by 40 mg every-other-week starting at Week 1. They will also receive MTX oral at the Baseline visit, with initial dose of 15mg/week, increasing up to 25 mg/week;

Arm 2: MTX with the same schedule as in Arm 1;

Arm 3: ADA with the same schedule as in Arm 1.

In addition to the study drugs, topical adjunctive eye medication will be allowed according to standard practice (intraocular pressure-lowering medication, cycloplegic agents, artificial tears, topical non-steroidal anti-inflammatory drugs…). The use of any biologic therapies with a potential therapeutic impact in NIUs, live vaccines, any other ISD besides the study drugs, intraocular GCs implants, or intraocular surgery will be prohibited for the duration of the trial.

To ensure a balance of patients across treatment groups and uveitis anatomic locations, patients will be stratified according to the main site of ocular inflammation (intermediate, OR posterior/panuveitis). Randomization will not be stratified by site due to the small-expected number of subjects per site.

For peer review only - http://bmjopen.bmj.com/site/about/guidelines.xhtml

For peer review only

Block randomization will ensure that an equal number of patients are randomized to each study arm.

The allocation sequence will consist of a computer-generated random number list generated and held in the Clinical Research Unit of Hospital Clínico San Carlos (HCSC), hidden from participating Investigators. The allocation sequence will be computer-generated, and it will be implemented through the electronic case report form (eCRF; REDCap), which will assign the treatment group. Study data will be collected and managed using REDCap electronic data capture tools hosted at The Health Research Institute of the Hospital Clínico San Carlos[62,63] REDCap (Research Electronic Data Capture) is a secure, web-based software platform designed to support data capture for research studies, providing 1) an intuitive interface for validated data capture; 2) audit trails for tracking data manipulation and export procedures; 3) automated export procedures for seamless data downloads to common statistical packages; and 4) procedures for data integration and interoperability with external sources. After obtaining the informed consent for enrolment and confirming that all eligibility requirements have been met, the Unmasked Investigator (see next section) will log into the eCRF and perform the randomization. Unmasked investigators will then give their assigned treatment to the subjects.

Authorized Rescue Medication

During whole study up to two inflammatory relapses (unilateral or bilateral) will be allowed, one during the first period (baseline-week 16: ACC relapse) and 1 during the second period (week 16 -week 52: any location), before declaring the lack of response to the assigned medication. Based on the location and severity of the relapse, a protocolized rescue treatment with Topical AND/OR Local OR Oral GCs will be

For peer review only

For peer review only - http://bmjopen.bmj.com/site/about/guidelines.xhtml

allowed. In case inflammation cannot be supressed in 4 weeks, or a new relapse takes place after 4 weeks, it will be declared treatment failure and the subject will exit the study.

Masking

For the duration of the trial, both the study subject and the Unmasked Investigators will be aware of the treatment assigned. Considering the differences between study drugs (MTX, ADA) regarding appearance, route of administration and schedule, in order to make them unaware of the medication prescribed, the use of placebo would be necessary. However, taking into account that both drugs have been proven effective in the treatment of non-infectious uveitis, the duration of the trial (up to 52 weeks), and the need for bi-weekly subcutaneous injections of one of the drugs (ADA), we consider unethical for the subject the use of placebo in the present trial.

Besides the Unmasked Investigators, the rest of participating investigators will be considered Masked Investigators (ophthalmologists performing the clinical eye exams, visual acuity examiners, OCT operators, fundus photographers, fundus graders, and administrators of subject's questionnaires) and will not be aware of the treatment assigned to prevent bias in study outcomes.

Several steps will be taken to avoid the Masked Investigators discovering the subject assignment: they will have no part in handling or prescribing medication; subjects will be given dark bags to place and keep their medication in throughout the trial and study visits to minimize the chances of the Masked Investigators seeing the medications. Additionally, subjects will meet with the Unmasked Investigator first, before seeing any Masked Investigators, keeping any study medication in his/her office for the entire

For peer review only - http://bmjopen.bmj.com/site/about/guidelines.xhtml

patient visit; reviewing the appearance of any AE with the subjects before seeing any Masked Investigators, and reminding the subjects not to discuss their dosing and mediation name with the Masked Investigators.

Study Procedures

Study visits will be the Baseline visit, visits at Week 1, 4 and every 4 weeks thereafter until a) the subject is determined as unable to achieve complete resolution of the ocular inflammatory process by week 16, OR; b) the subject is determined as unable to maintain a complete resolution of the ocular inflammatory process, between weeks 16 and 52 OR; c) the subject completes 52 weeks of this clinical trial, OR; d) the study is stopped due to the findings of the Data Security Monitoring Board, OR; e) the subject meets any of the study finalization criteria.

Informed consent has to be acquired before carrying out any study procedure, including screening tests (**supplementary file 1** contains a sample informed consent). Before Baseline (when the subject is randomized), several screening visits can take place up to 14 days before that visit, in order to obtain all the required complementary tests to assess eligibility. The visit window for all scheduled visits is ±3 days through Week 4 and ±7 days for all visits following the Week 4 study visit. **Table 3** provides an overview of the study activities.

After the trial, all patients will return to standard care and will be able to continue with their assigned study medication.

Table 3: Study activities.

Activity	Screening	Baseline	w1	w4	w8	w12	w16	w20	w24	w28	w32	w36	w40	w44	w48	FET	Relapse	Unsch.	70-day Follow-up

For peer review only - http://bmjopen.bmj.com/site/about/guidelines.xhtml

Informed Consent	X																		
Inclusion/Exclusion Criteria	X	X																	
Medical/Surgical History	X	X																	
History of tobacco and alcohol use	X	X																	
Uveitis History	X	X																	
Vital Signs/ Weight/Height	X	X														X	(X)		
Physical Exam	X	X														X	(X)		
Symptom Directed Physical Exam			X	X	X	X	X	X	X	X	X	X	X	X	X		X		
Chest X-Ray	X															X[g]			
Cerebral MRI[a]	(X)																		
TB Screening	X																		
Serum Pregnancy Test[f]	X	X	X	X	X	X	X	X	X	X	X	X	X	X	X	X			
Hepatitis B/C Screening	X																		
Syphilis Testing (FTA)	X																		
Randomization		X																	
Best Corrected Visual Acuity Testing	(X)[b]	(X)[b]		X	X	X	X	X	X	X	X	X	X	X	X	X	X		
Tonometry	(X)[b]	(X)[b]		X	X	X	X	X	X	X	X	X	X	X	X	X	X		
Slit Lamp Exam	(X)[b]	(X)[b]		X	X	X	X	X	X	X	X	X	X	X	X	X	X		
Dilated Indirect Ophthalmoscopy	(X)[b]	(X)[b]		X	X	X	X	X	X	X	X	X	X	X	X	X	X		
Fundus Photography	(X)[b]	(X)[b]					X									X	X		
Optical Coherence Tomography	(X)[b]	(X)[b]		X	X	X	X	X	X	X	X	X	X	X	X	X	X		
EuroQol-5D	(X)[b]	(X)[b]		X	X	X	X	X	X	X	X	X	X	X	X	X	X		
Visual Functioning Questionnaire-25	(X)[b]	(X)[b]					X									X	X		
Hospital Anxiety and Depression Scale	(X)[b]	(X)[b]					X									X	X		
Hematology/Chemistry	X	(X)[c]	X	X	X	X	X		X		X		X		X	X	(X)		
Monitor Adverse Events		(X)	X	X	X	X	X	X	X	X	X	X	X	X	X	X	X	X	X
Monitor Concomitant Medication	X	X	X	X	X	X	X	X	X	X	X	X	X	X	X	X	X	X	X

For peer review only - http://bmjopen.bmj.com/site/about/guidelines.xhtml

Review of Patient Diary			X	X	X	X	X	X	X	X	X	X	X	X	X	X	(X)		
Perform Drug Accountability			X	X	X	X	X	X	X	X	X	X	X	X	X	X	(X)		
Assessment of direct and indirect costs			X	X	X	X	X	X	X	X	X	X	X	X	X	X	(X)		
Dispense Study Drugs		X	X	X	X	X	X	X	X	X	X	X	X	X	X		(X)		
Proteomic blood sample	(X)[d]	(X)[d]					X									X	X		
Pharmacogenetic blood sample	(X)[d]	(X)[d]																	
Adalimumab levels and Anti-Adalimumab Antibodies	(X)[d]	(X)[d]					X[e]			X[e]						X[e]	X[e]		

FET: Final (w52) / Early termination visit. Unsch: Unscheduled visit.

[a]Cerebral MRI will be performed only in those subjects with intermediate uveitis or signs of intermediate uveitis.

[b]Values from the Baseline visit or from a screening visit up to 14 days before the Baseline Visit will be used as the values from the Baseline visit.

[c]Total Blood count, renal and liver function test from up to 4 weeks before the Baseline visit will the required before entering the study.

[d]Blood sample will be drawn after signature of the informed consent, as soon as possible, ideally before start of oral GCs.

[e]Blood samples for the Anti-Adalimumab Antibodies will be drawn the week before visits 16, 28, 52, Early termination and relapse visits, up to 24h before the patient is scheduled to receive the ADA dosage, and always before the medication is taken.

[F] Only in women in childbearing

[g]Chest X ray will be performed at the 52 weeks/Early termination visit in case the patient had a positive TB test at abaseline.

Data Management and Monitoring

For peer review only - http://bmjopen.bmj.com/site/about/guidelines.xhtml

An Unmasked Investigator in each centre will review and crosscheck for consistency and completeness all data collected in RedCap within 24h of the study visit.If the forms are not filled out completely, the responsible person will be contacted for providing the missing data.. An external monitoring service will conduct regular checks of the data regarding errors and inconsistencies, supervising data collection, management and quality control, and will submit queries to the site investigators.

Safety

Non-serious AEs will be defined as an unfavorable and unintended sign (including abnormal laboratory findings), symptom or disease temporally associated with the use of the study medication or procedure, whether or not considered related to the study medication. Both MTX and ADA may be temporarily suspended in case of non-serious AEs, such as laboratory alterations, infections, and intolerance. In addition, MTX dosage may also be reduced in case of laboratory alterations and/or intolerance. Dose reductions and temporary discontinuations will be decided by the Masked Investigator, who must remain unaware of the medication and dosage assigned to the subject, so preconceptions regarding the study drugs do not interfere with their management. The Masked Investigator will issue three recommendations; one for each medication arm the subject may be included. Then, the Unmasked Investigator will implement the recommendation according to the arm the subject is included.

SAEs will be defined as any adverse event that results in death, is life threatening, requires hospitalization, or prolongation of existing hospitalization, results in persistent or significant disability or incapacity, or is a congenital anomaly or birth defect.

All AEs will be recorded in clinical records and a medically qualified investigator will assess the relationship of SAEs to the study medications.

For peer review only - http://bmjopen.bmj.com/site/about/guidelines.xhtml

All SAE that occurs during the trial must be reported immediately by mail or fax to the Pharmacovigilance Unit within 24 hours of its occurrence. The investigator will complete and sign the SAE notification form to be sent by e-mail.

The Pharmacovigilance Unit will review the form received and, if applicable, ask for additional information to the investigator. The investigator should provide the requested information or any new information regarding the case, especially if the initial assessment in severity or causality has been changed, following the procedure previously described.

The Pharmacovigilance Unit is responsible for submitting as soon as possible all Suspected Unexpected Serious Adverse Reactions (SUSARs) collected during the study to the Spanish Health Authorities and Ethic Committee, according with the Spanish legislation: no later than 15 calendar days (7 in case of fatal or life-threatening cases) after first knowledge by the sponsor that the case meets the minimum criteria for expedited reporting.

Data and Safety Monitoring Committee

Only after the Data and Safety Monitoring Committee (DSMC) reviews and approves the protocol will patients be enrolled. In addition, the group will meet regularly throughout the study and review information on data quality, enrolment, patient retention, and study outcomes according to DSMC charter. The DSMC will be independent from the Sponsor, Funding Body and Principal Investigator, and will include experts in the fields of ophthalmology, rheumatology and epidemiology.

Adherence

Adherence will be monitored using a Patient's Diary (where they will register all study medication administered outside of the study visit (i.e., at home), including reasons for

For peer review only - http://bmjopen.bmj.com/site/about/guidelines.xhtml

missing dosages) and by verifying the returned empty medication (partially/completely empty blisters of study medication, AND/OR cartons and sharps containers for MTX, ADA and oral GCs).

Patient and Public Involvement

No patient involved.

Statistical analysis plan

The Intention to treat (ITT) set will include all subjects who were randomized.

The safety set will consist of all subjects who received at least one dose of study medications. Per protocol analysis will also carry out. Missing data will be imputed using multiple imputation-chained equations

Sample Size Determination

ADA arm: after one year (50 weeks) of treatment, 20.3% of NIU patients achieved an outcome similar to our *primary efficacy outcome*[64];

MTX arm: after 6 months of treatment, 65% of NIU patients achieve also similar outcome[65]; we estimate that of those patients, 18%[66] and 16%[67] will be unable to maintain our outcome due to AEs and inefficacy, respectively; therefore, at 1 year, 43% will achieve our *primary efficacy outcome*. This figure will be assumed as the percentaje of subjects treated with monotherapy achieving the primary efficacy outcome.

MTX+ADA arm: we will assume that combination therapy will increase the percentage of subjects achieving our *primary efficacy outcome* by 23% compared with the monotherapy arms.

For peer review only - http://bmjopen.bmj.com/site/about/guidelines.xhtml

To detect statistically significant differences between groups with a power of 80% and a significance level of 0.05, it will be necessary to recruit 54 patients per study arm (162 in total). Since the follow-up period is 52 weeks, losses of 15% will be assumed, increasing the sample size to 64 patients per study arm (192 patients in total).

Planned Methods of Statistical Analysis for efficacy-related objectives

The primary analysis will be a Mantel–Haenszel test (MHT), stratified by NIU location, comparing the combination therapy arm and the single immunosuppressive drugs arms (both arms combined), and performed in the ITT set. Achievement of the Good Clinical response will be considered the binary response variable: those subjects achieving a Good Clinical vs. those not achieving the outcome (regardless the cause). The exposure variables will be the treatment arm: those subjects receiving combination therapy vs. those receiving either monotherapy. Uveitis location will be the strata: intermediate uveitis vs. posterior OR panuveitis. If the results of the primary analysis are significant, then pairwise comparisons using MHT stratified by NIU location will be carried out. P-values of the pairwise comparisons will be adjusted using the Bonferroni method. No interim efficacy-related analysis will be carried out.

Secondary efficacy-related analyses are designed to test the hypothesis that treatment assignment affects a given outcome, after controlling for selected covariates. Details can be found at **Table 4**.

Table 4: Planned Methods of Statistical Analysis for efficacy-related secondary outcomes.

Outcome variable	Statistical Analysis
Complete abrogation of the ocular inflammatory signs, which is achieved within the	MHT, stratified by NIU location. If p-value < 0.05, pairwise comparisons using MHT stratified by NIU location will be

For peer review only - http://bmjopen.bmj.com/site/about/guidelines.xhtml

first 16 weeks of the study, no treatment failure due to safety or intolerability; compliance with the initial (up to week 16) oral GCs tapering protocol, and completion of all study visits from Baseline to 16 weeks	carried out with Bonferroni adjustment of the pairwise p-values
EQ5D	GEE models nested by patient[68,69] and adjusted by study visit (continuous) and treatment arm (discrete) will be carried out, using a Gaussian family and Identity as link function. Different covariable structures will be tested (independent and exchangeable) and compared using the Bayesian Information Criteria. Time x Study arm interactions will assess different effects of time in the evolution of the outcome by arm. p-value <0.05 will be considered as a significant interaction
Visual Functioning Questionnaire-25<br>Hospital Anxiety and Depression Scale	Changes between Baseline and w16, and the FET visit will be compared between treatment groups using ANOVA
Active chorioretinal lesions; active retinal vascular lesions; macular edema; ACC; vitreous haze; and loss of CVA secondary to inflammation at baseline, week 16, week 52, and relapse visit	MHT, stratified by NIU location. If p-value < 0.05, pairwise comparisons using MHT stratified by NIU location will be carried out with Bonferroni adjustment of the pairwise p-values
The time to inflammatory relapse between groups, defined as the time from visit 16 weeks until end of the study, loss of follow-up or appearance of at least one ocular inflammatory manifestation, in those individuals achieving a Good Clinical Response by visit 16 week	Time to relapse between arms will be analyzed using log-rank test at a two-sided significance level of 5%. Dropouts due to reasons other than inability to maintain a Good Clinical Response will be considered as censored observations at the time of dropping out. Only subjects able to achieve a Good Clinical Response by visit week 16 will be analyzed. We will consider both the time until the onset of the first inflammatory manifestation, each inflammatory manifestation, the first inflammatory manifestation that does not resolve in the following 4 weeks, and each inflammatory manifestation that does not resolve in the following 4 weeks.
Best corrected visual acuity during follow-up	GEE models
Assessment of anti-ADA antibodies (AAA) at week 15, 27, 51, and relapse.	MHT, stratified by NIU location. If p-value < 0.05, pairwise comparisons using MHT stratified by NIU location will be carried out with Bonferroni adjustment of the pairwise p-values
Direct and indirect cost, and Incremental Cost Effectiveness Ratios	Cost utility and cost-effectiveness analysis from the perspectives of the National Health System and the Society will be performed. EQ5D scores will derive utility values representing health related quality of life. QALYs will be calculated by the area under the curve assuming a linear evolution of EQ5D values between visits. The average

For peer review only - http://bmjopen.bmj.com/site/about/guidelines.xhtml

	number of QALYs per patient will be calculated for each study arms. Effectiveness will be defined using our primary efficacy outcome. An average cost per patient will be calculated for each study arm, including direct (drug cost, outpatient and inpatient care, and other medical cost), and indirect cost (home care, productivity loss): Incremental cost-effectiveness ratios will be calculated, using the MTX monotherapy arm as comparison

GCs: Glucocorticoids; MTX: Methotrexate; ADA: Adalimumab; QALYs: Quality-adjusted life years; EQ5D: EuroQuol 5D-5L; GEE: Generalized estimating equations; MTH: Mantel-Haetzel; NIU: Non-infectious uveitis

Planned Methods of Statistical Analysis for Safety-related aims

The safety analysis will be performed in the safety set. Treatment-emergent AEs (events with an onset date on or after the first study drug administration until 70 days following the last study drug administration) will be summarized by treatment group using descriptive statistics. SAEs with onset after informed consent but before the first study drug administration will be considered as pretreatment SAEs and reported separately. AEs will be tabulated by system organ class and preferred term whereby the most current implemented MedDRA dictionary will be used. In addition, summaries by severity and relationship to study drug will be done. Certain AEs, such as serious or severe, leading to premature withdrawal, will be listed and described in detail. AEs of special interest for treatments will be defined in the Statistical analysis plan (SAP) and analysed separately. In addition to the descriptive statistics provided, Fisher's exact test will be used for comparisons between treatment groups.

Genetic Analysis

SNPs genotypes will be determined by real-time PCR amplification using Taqman probes and following standard procedures. Duplicate genotypes of 10% of the samples,

For peer review only - http://bmjopen.bmj.com/site/about/guidelines.xhtml

concordance (all $p > 0.05$) with the Hardy–Weinberg equilibrium and with SNP frequencies in the HapMap European collection will be used for quality control. Comparison of the proportion of subjects achieving the primary efficacy outcome between genotypes will be carried out using a χ^2 test or Fisher's exact test, when required. Dominant, recessive, and additive models of effects will be considered for each SNP. P-values will be adjusted using the Bonferroni method. Each study arm will be analysed separately.

Proteomic Analyses

Discovery Phase: Shotgun proteomic analysis[56–58] will be performed on serum samples from a representative group of subjects from each study arm with extreme responses (n=20): those achieving the *primary efficacy outcome* and those not being able to achieve a good clinical response by Week 16.

Verification Phase: Targeted proteomics will be used for verification of protein markers with predictive potential in a randomly selected larger samples set (n=80). After multiple reaction monitoring (MRM) tests, relative quantification methods of the proteins will be designed[59].

Validation Phase: Best candidates from the previous phase will be validated using absolute quantification tests (antibody-based microarrays) in the whole set of subjects[60,61].

Patient subgroup identification

Based on Baseline visits patient's characteristics (demographic, disease, and clinical-related variables), those genotypes significantly associated with the primary efficacy outcome, and the previously identified serum proteins in the verification phase,

For peer review only - http://bmjopen.bmj.com/site/about/guidelines.xhtml

prediction models for MTX, ADA, and Combination therapy response will be developed using a machine learning method (Random Forests[70]). Models' performance will be assessed with the area under the receiver operating characteristic curve, and calibration curves. Models using only clinical data, only biomarkers data, and the combination of both will be developed, to assess the contribution of biomarkers to the models' predictive ability. Due to the modest sample size we plan to recruit, we will not divide our sample in training, validation and test datasets. All subjects will be considered part of the training dataset, and a ten-fold cross-validation will be carried out to internally validate our models.

ETHICS AND DISSEMINATION

The protocol (version 2, 11/09/2020), annexes and informed consent forms have been approved by the Clinical Research Ethic Committee (CREC) at the Hospital Clínico San Carlos (Madrid, Spain) and the Spanish Agency for Medicines and Health Products (AEMPS). The local approvals corresponding to the participating centers will be obtained and documented before starting the study in that center as per center requirements. The promoter will be the CREC interlocutor corresponding to his/her center in everything related to the present study. It will keep CREC informed of the evolution of the study in the center and of the possible minor incidents and modifications that may occur. Any relevant modification to the protocol must receive express approval from the reference CREC and the AEMPS before its implementation, unless there are risk circumstances for the participating subjects, in which case the precise measures to ensure the integrity of the study subjects will be implemented immediately, pending the corresponding approvals. The trial is registered at

For peer review only - http://bmjopen.bmj.com/site/about/guidelines.xhtml

clinicaltrialsregister.eu (EudraCT:2020-000130-18) and clinicaltrials.gov (NCT04798755).

No study related activities will be carried out before obtaining a written informed consent from the patient. The investigator will be responsible for a) providing each patient with an information sheet about the trial and the objectives, methods, foreseeable benefits and potential risks of the study, b) discussing the information with the patient, in terms understandable for the subject, and c) explaining to patients that they are totally free to refuse their participation in the study or to abandon it at any time and for any reason. If the subject agrees to participate in the Biobank Substudy, a second independent informed consent will be collected, which will include the possibility of storing the samples not used in the present study in the Collection of Samples for Research in Rheumatic Diseases of the Rheumatology Department of the Hospital Clinico San Carlos (and, in a second phase, when the Coordinating Investigator of this study deems it appropriate and always in the event that the samples has not been used up, the remainder will be stored in the Hospital Clinico San Carlos Biobank).

All the data will be treated confidentially at any times: data will be seudonimized, the paper forms will be kept in locked cabinets, the eCRFis located in a secure server, and the person in charge of the analysis will not be able to access identification data of the subjects.

Table 5 summarises the study protocol and trial registration information.

Table 5: Trial registration data and protocol summary

Data category	Information
Primary registry and trial identifying number	EudraCT: 2020-000130-18

For peer review only - http://bmjopen.bmj.com/site/about/guidelines.xhtml

Date of registration in primary registry	09/03/2021
Secondary identifying numbers	ClinicalTrials.gov: NCT04798755
Source of monetary or material support	Instituto de Salud Carlos III
Primary sponsor	Fundación para la Investigación Biomédica del Hospital Clínico San Carlos
Contact for queries	Luis Rodriguez-Rodriguez, MD (lrrodriguez@salud.madrid.org)
Public Title	Combination THerapy with mEthotrexate and adalImumAb for uveitis (CoTHEIA)
Scientific title	Efficacy , safety and cost-effectiveness of methotrexate, adalimumab, or their combination in non-infectious non-anterior uveitis: a multicenter, randomized, parallel 3 arms, active-controlled, phase 3 open label with blinded outcome assessment study
Countries of recruitment	Spain
Health condition or problem studied	Non-infectious non anterior uveitis
Intervention(s)	Intervention 1: Adalimumab 40 mg every-other-week (plus a 80 mg SC loading dose) + Methotrexate oral, up to 25 mg/week, both for a duration of 52 weeks.
	Intervention 2: Methotrexate with the same schedule as in Intervention 1
	Intervention 3: Adalimumab with the same schedule as in Intervention 1
Key inclusion and exclusion criteria	Age ≥18 years old<br>Diagnosed with non-infectious intermediate, posterior, or panuveitis in at least one eye;<br>Active ocular disease<br>Lack of satisfactory response to systemic and/or local glucocorticoid (GC) therapy AND/OR diagnoses of a chronic disease usually necessitating GC-sparing immunosuppressive treatment.
Study type	Phase III, multicenter, prospective, randomized, single-blinded with masked outcome assessment, parallel 3 arms with 1:1:1 allocation, active-controlled, superiority study design
Date of first enrolment	N/A
Target sample size	64 per treatment arm (192 in total)
Recruitment status	Not yet recruiting
Primary outcome	Complete resolution of the ocular inflammatory signs, which is achieved within the first 16 weeks of the study, and maintained during follow-up until the end of the study (week 52);

For peer review only - http://bmjopen.bmj.com/site/about/guidelines.xhtml

Key secondary outcomes	Safety, cost-effectiveness

Data obtained through this study may be provided to qualified researchers with academic interest in uveitis. Data or samples shared will be coded, and donated to a Registered Biobank and made available under legal requirement. Approval of the request and execution of all applicable agreements are prerequisites to the sharing of data with the requesting party.

Regarding dissemination, in order to communicate the clinical trial progress and findings to a broad group of stakeholders, we will elaborate a Dissemination plan which will include production of materials adapted to scientific meetings, scientific publications, patients, and other stakeholders. A summary of the final version of the study protocol will be made available through the Spanish Clinical Trial Registry and Clinicaltrials.gov database. The promoter will be the only with access to the participant-level data, following the regulation on data protection.

CONTRIBUTORSHIP STATEMENT

Luis Rodriguez-Rodriguez (LRR), Amanda López Picado (ALP), Esperanza Pato Cour (EPC) and David Diaz Valle (DDV) conceived of the study.

LRR, ALP, EPC, DDV, Ester Carreño (EC), Miguel Cordero-Coma (MCC), Alex Fonollosa (AF), Angel Garcia Aparicio (AGA), Javier Garcia Gonzalez (JGG), Santiago Muñoz (SM), Ana Rivas (AR), and Luis Tebar (LT) initiated the study design and helped with implementation.

LRR is the funding holder.

ALP and AR provided statistical expertise in clinical trial design.

Valentina Calamia (VC) provided expertise regarding the proteomic analysis design.

For peer review only - http://bmjopen.bmj.com/site/about/guidelines.xhtml

LRR provided expertise regarding the pharmacogenetic analysis.

ALP, AR and LRR will conduct the primary statistical analysis.

AR, ALP, VC, EC, Lidia Cocho (LC), MCC, AF, Felix M Francisco Hernandez (FMFH), AGA, JGG, Jose Juan Mondejar (JJM), Leticia Lojo Oliveira (LLO), Llucí Martinez-Costa (LMC), SMM, Diana Peiteado (DP), Jose Antonio Pinto (JAP), Beatriz Rodriguez Lozano (BRL), EPC, DDV, Elena Molina (EM), LT and LRR contributed to refinement of the study protocol and approved the final manuscript.

For peer review only

COMPETING INTERESTS

None declared

FUNDING

This work was supported by the Instituto de Salud Carlos III, grant number [ICI19/00020].

REFERENCES

1 Caspi RR. A look at autoimmunity and inflammation in the eye. *J Clin Invest* 2010;**120**:3073–83. doi:10.1172/JCI42440

2 WHO. The World Health Report, Global data on visual impairments. 2010.http://www.who.int/blindness/GLOBALDATAFINALforweb.pdf

3 Suttorp-Schulten MS, Rothova A. The possible impact of uveitis in blindness: a literature survey. *Br J Ophthalmol* 1996;**80**:844–8. doi:10.1136/bjo.80.9.844

4 Rothova A, Suttorp-van Schulten MS, Frits Treffers W, *et al.* Causes and frequency of blindness in patients with intraocular inflammatory disease. *Br J Ophthalmol* 1996;**80**:332–6.http://www.pubmedcentral.nih.gov/articlerender.fcgi?artid=505460&tool=pmcentrez&rendertype=abstract (accessed 25 Jul 2014).

5 de Smet MD, Taylor SRJ, Bodaghi B, *et al.* Understanding uveitis: The impact of research on visual outcomes. *Prog Retin Eye Res* 2011;**30**:452–70. doi:10.1016/j.preteyeres.2011.06.005

6 Williams GJ, Brannan S, Forrester J V, *et al.* The prevalence of sight-threatening uveitis in Scotland. *Br J Ophthalmol* 2007;**91**:33–6. doi:10.1136/bjo.2006.101386

7 Gritz DC, Wong IG. Incidence and prevalence of uveitis in Northern California: The Northern California Epidemiology of Uveitis Study. Ophthalmology. 2004;**111**:491–500. doi:10.1016/j.ophtha.2003.06.014

8 Chang JH-M, Wakefield D. Uveitis: a global perspective. *Ocul Immunol Inflamm* 2002;**10**:263–79. doi:10.1076/ocii.10.4.263.15592

9 Arriola-Villalobos P, Abásolo L, García-Feijoo J, *et al.* Vision-related Quality of Life in Patients with Non-infectious Uveitis: A Cross-sectional Study. *Ocul Immunol Inflamm* 2017;:1–9. doi:10.1080/09273948.2017.1285034

10 Miserocchi E, Modorati G, Mosconi P, *et al.* Quality of life in patients with uveitis on chronic systemic immunosuppressive treatment. *Ocul Immunol Inflamm* 2010;**18**:297–304. doi:10.3109/09273941003637510

11 DARRELL RW, WAGENER HP, KURLAND LT. Epidemiology of uveitis. Incidence and prevalence in a small urban community. *Arch Ophthalmol (Chicago, Ill 1960)* 1962;**68**:502–

For peer review only

For peer review only - http://bmjopen.bmj.com/site/about/guidelines.xhtml

14.http://www.ncbi.nlm.nih.gov/pubmed/13883604

12 Suhler EB, Lloyd MJ, Choi D, *et al.* Incidence and prevalence of uveitis in Veterans Affairs Medical Centers of the Pacific Northwest. *Am J Ophthalmol* 2008;**146**:890-6.e8. doi:10.1016/j.ajo.2008.09.014

13 E. Kirbach, S., A. Hayes, O., A. Cifaldi M. The Economic Burden of Uveitis. *Arthritis Rheum* 2010;**62**:788. doi:10.1002/art.28556

14 Adán-Civera AM, Benítez-Del-Castillo JM, Blanco-Alonso R, *et al.* Burden and direct costs of non infectious uveitis in Spain. *Reumatol Clin* 2015;**12**:196–200. doi:10.1016/j.reuma.2015.08.004

15 Durrani OM, Meads CA, Murray PI. Uveitis: A potentially blinding disease. Ophthalmologica. 2004;**218**:223–36. doi:10.1159/000078612

16 Jalil A, Yin K, Coyle L, *et al.* Vision-related quality of life and employment status in patients with uveitis of working age: a prospective study. *Ocul Immunol Inflamm* 2012;**20**:262–5. doi:10.3109/09273948.2012.684420

17 Dick AD, Rosenbaum JT, Al-Dhibi HA, *et al.* Guidance on Noncorticosteroid Systemic Immunomodulatory Therapy in Noninfectious Uveitis. *Ophthalmology* 2018;**125**:757–73. doi:10.1016/j.ophtha.2017.11.017

18 Smith JR, Rosenbaum JT. Management of uveitis: a rheumatologic perspective. *Arthritis Rheum* 2002;**46**:309–18. doi:10.1002/art.503

19 Jabs D a, Rosenbaum JT, Foster CS, *et al.* Guidelines for the use of immunosuppressive drugs in patients with ocular inflammatory disorders: recommendations of an expert panel. *Am J Ophthalmol* 2000;**130**:492–513. doi:10.1016/S0002-9394(00)00659-0

20 Becker MD, Smith JR, Max R, *et al.* Management of sight-threatening uveitis: new therapeutic options. *Drugs* 2005;**65**:497–519. doi:10.2165/00003495-

For peer review only - http://bmjopen.bmj.com/site/about/guidelines.xhtml

For peer review only

200565040-00005

21 Jabs DA. Immunosuppression for the Uveitides. *Ophthalmology* 2018;**125**:193–202. doi:10.1016/j.ophtha.2017.08.007

22 Rathinam SR, Babu M, Thundikandy R, *et al.* A randomized clinical trial comparing methotrexate and mycophenolate mofetil for noninfectious uveitis. *Ophthalmology* 2014;**121**:1863–70. doi:10.1016/j.ophtha.2014.04.023

23 Rathinam SR, Gonzales JA, Thundikandy R, *et al.* Effect of Corticosteroid-Sparing Treatment With Mycophenolate Mofetil vs Methotrexate on Inflammation in Patients With Uveitis: A Randomized Clinical Trial. *JAMA* 2019;**322**:936–45. doi:10.1001/jama.2019.12618

24 Gangaputra S, Newcomb CW, Liesegang TL, *et al.* Methotrexate for Ocular Inflammatory Diseases. *Ophthalmology* 2009;**116**:2188–99. doi:10.1016/j.ophtha.2009.04.020

25 Salliot C, van der Heijde D. Long-term safety of methotrexate monotherapy in patients with rheumatoid arthritis: a systematic literature research. *Ann Rheum Dis* 2009;**68**:1100–4. doi:10.1136/ard.2008.093690

26 Baker KB, Spurrier NJ, Watkins AS, *et al.* Retention time for corticosteroid-sparing systemic immunosuppressive agents in patients with inflammatory eye disease. *Br J Ophthalmol* 2006;**90**:1481–5. doi:10.1136/bjo.2006.097998

27 Abásolo L, Rosales Z, Díaz-Valle D, *et al.* Immunosuppressive Drug Discontinuation in Noninfectious Uveitis From Real-Life Clinical Practice: A Survival Analysis. *Am J Ophthalmol* 2016;**169**:1–8. doi:10.1016/j.ajo.2016.06.007

28 Jaffe GJ, Dick AD, Brézin AP, *et al.* Adalimumab in Patients with Active Noninfectious Uveitis. *N Engl J Med* 2016;**375**:932–43.

For peer review only - http://bmjopen.bmj.com/site/about/guidelines.xhtml

doi:10.1056/NEJMoa1509852

29 Nguyen QD, Merrill PT, Jaffe GJ, *et al.* Adalimumab for prevention of uveitic flare in patients with inactive non-infectious uveitis controlled by corticosteroids (VISUAL II): a multicentre, double-masked, randomised, placebo-controlled phase 3 trial. *Lancet (London, England)* 2016;**388**:1183–92. doi:10.1016/S0140-6736(16)31339-3

30 Arida A, Fragiadaki K, Giavri E, *et al.* Anti-TNF agents for Behçet's disease: analysis of published data on 369 patients. *Semin Arthritis Rheum* 2011;**41**:61–70. doi:10.1016/j.semarthrit.2010.09.002

31 Leinonen ST, Aalto K, Kotaniemi KM, *et al.* Anti-adalimumab antibodies in juvenile idiopathic arthritis-related uveitis. *Clin Exp Rheumatol*;**35**:1043–6.http://www.ncbi.nlm.nih.gov/pubmed/29148422

32 Llorenç V, Cordero-Coma M, Blanco-Esteban A, *et al.* Drug Retention Rate and Causes of Discontinuation of Adalimumab in Uveitis: Real-World Data from the Biotherapies in Uveitis (BioÚvea) Study Group. *Ophthalmology* 2020;**127**:814–25. doi:10.1016/j.ophtha.2019.11.024

33 Michaud TL, Rho YH, Shamliyan T, *et al.* The comparative safety of tumor necrosis factor inhibitors in rheumatoid arthritis: a meta-analysis update of 44 trials. *Am J Med* 2014;**127**:1208–32. doi:10.1016/j.amjmed.2014.06.012

34 Bongartz T, Sutton AJ, Sweeting MJ, *et al.* Anti-TNF antibody therapy in rheumatoid arthritis and the risk of serious infections and malignancies: systematic review and meta-analysis of rare harmful effects in randomized controlled trials. *JAMA* 2006;**295**:2275–85. doi:10.1001/jama.295.19.2275

35 Joshi L, Talat L, Yaganti S, *et al.* Outcomes of changing immunosuppressive therapy after treatment failure in patients with noninfectious uveitis.

For peer review only - http://bmjopen.bmj.com/site/about/guidelines.xhtml

Ophthalmology 2014;**121**:1119–24. doi:10.1016/j.ophtha.2013.11.032

36 Menezo V, Lau C, Comer M, *et al.* Clinical outcome of chronic immunosuppression in patients with non-infectious uveitis. *Clin Experiment Ophthalmol* 2005;**33**:16–21. doi:10.1111/j.1442-9071.2005.00904.x

37 Goldberg NR, Lyu T, Moshier E, *et al.* Success with single-agent immunosuppression for multifocal choroidopathies. *Am J Ophthalmol* 2014;**158**:1310–7. doi:10.1016/j.ajo.2014.08.039

38 Cervantes-Castañeda RA, Gonzalez-Gonzalez LA, Cordero-Coma M, *et al.* Combined therapy of cyclosporine A and mycophenolate mofetil for the treatment of birdshot retinochoroidopathy: a 12-month follow-up. *Br J Ophthalmol* 2013;**97**:637–43. doi:10.1136/bjophthalmol-2012-302123

39 Akpek EK, Baltatzis S, Yang J, *et al.* Long-term immunosuppressive treatment of serpiginous choroiditis. *Ocul Immunol Inflamm* 2001;**9**:153–67.http://www.ncbi.nlm.nih.gov/pubmed/11815884

40 Hooper PL, Kaplan HJ. Triple agent immunosuppression in serpiginous choroiditis. *Ophthalmology* 1991;**98**:944–51; discussion 951-2.http://www.ncbi.nlm.nih.gov/pubmed/1866149

41 Pascalis L, Pia G, Aresu G, *et al.* Combined cyclosporin A-steroid-MTX treatment in endogenous non-infectious uveitis. *J Autoimmun* 1993;**6**:467–80. doi:10.1006/jaut.1993.1039

42 Agarwal M, Ganesh SK, Biswas J. Triple agent immunosuppressive therapy in Vogt-Koyanagi-Harada syndrome. *Ocul Immunol Inflamm* 2006;**14**:333–9. doi:10.1080/09273940600976938

43 Arcinue CA, Radwan A, Lebanan MO, *et al.* Comparison of two different combination immunosuppressive therapies in the treatment of Vogt-Koyonagi-

For peer review only

For peer review only - http://bmjopen.bmj.com/site/about/guidelines.xhtml

Harada syndrome. *Ocul Immunol Inflamm* 2013;**21**:47–52. doi:10.3109/09273948.2012.728668

44 Davatchi F, Sadeghi Abdollahi B, Shams H, *et al.* Combination of pulse cyclophosphamide and azathioprine in ocular manifestations of Behcet's disease: longitudinal study of up to 10 years. *Int J Rheum Dis* 2014;**17**:444–52. doi:10.1111/1756-185X.12248

45 Katsuyama A, Kusuhara S, Nishisho R, *et al.* Long-term efficacy and safety of infliximab and cyclosporine combination therapy for refractory uveoretinitis in Behçet's disease. *Clin Ophthalmol* 2019;**13**:521–7. doi:10.2147/OPTH.S198648

46 Kotaniemi K. Late onset uveitis in juvenile-type chronic polyarthritis controlled with prednisolone, cyclosporin A and methotrexate. *Clin Exp Rheumatol*;**16**:469–71.http://www.ncbi.nlm.nih.gov/pubmed/9706430

47 Ganesh SK, Narayana KM, Biswas J. Peripapillary choroidal atrophy in sympathetic ophthalmia and management with triple-agent immunosuppression. *Ocul Immunol Inflamm* 2003;**11**:61–5.http://www.ncbi.nlm.nih.gov/pubmed/12854028

48 Cimino L, Finzi G, Mora P, *et al.* Effect of a drug combination treatment on ocular perfusion in recurrent idiopathic intermediate uveitis. *Ocul Immunol Inflamm* 2003;**11**:299–303.http://www.ncbi.nlm.nih.gov/pubmed/14704901

49 Hazlewood GS, Barnabe C, Tomlinson G, *et al.* Methotrexate monotherapy and methotrexate combination therapy with traditional and biologic disease modifying antirheumatic drugs for rheumatoid arthritis: abridged Cochrane systematic review and network meta-analysis. *BMJ* 2016;**353**:i1777.http://www.ncbi.nlm.nih.gov/pubmed/27102806

50 Ramanan A V, Dick AD, Jones AP, *et al.* Adalimumab plus Methotrexate for

For peer review only - http://bmjopen.bmj.com/site/about/guidelines.xhtml

Uveitis in Juvenile Idiopathic Arthritis. *N Engl J Med* 2017;**376**:1637–46. doi:10.1056/NEJMoa1614160

51 Kiss S, Ahmed M, Letko E, *et al.* Long-term follow-up of patients with birdshot retinochoroidopathy treated with corticosteroid-sparing systemic immunomodulatory therapy. *Ophthalmology* 2005;**112**:1066–71. doi:10.1016/j.ophtha.2004.12.036

52 Biomarkers Definitions Working Group. Biomarkers and surrogate endpoints: preferred definitions and conceptual framework. *Clin Pharmacol Ther* 2001;**69**:89–95. doi:10.1067/mcp.2001.113989

53 LaMattina KC, Escott SM, Malalis JF, *et al.* The Influence of Race and Sex on the Side Effect Profile of Methotrexate in the Treatment of Uveitis. *Ocul Immunol Inflamm* 2018;:1–5. doi:10.1080/09273948.2018.1498905

54 López-Rodríguez R, Ferreiro-Iglesias A, Lima A, *et al.* Replication study of polymorphisms associated with response to methotrexate in patients with rheumatoid arthritis. *Sci Rep* 2018;**8**:7342. doi:10.1038/s41598-018-25634-y

55 Bek S, Bojesen AB, Nielsen J V, *et al.* Systematic review and meta-analysis: pharmacogenetics of anti-TNF treatment response in rheumatoid arthritis. *Pharmacogenomics J* 2017;**17**:403–11. doi:10.1038/tpj.2017.26

56 Fernández-Puente P, Mateos J, Fernández-Costa C, *et al.* Identification of a panel of novel serum osteoarthritis biomarkers. *J Proteome Res* 2011;**10**:5095–101. doi:10.1021/pr200695p

57 Lourido L, Calamia V, Mateos J, *et al.* Quantitative proteomic profiling of human articular cartilage degradation in osteoarthritis. *J Proteome Res* 2014;**13**:6096–106. doi:10.1021/pr501024p

58 Lourido L, Calamia V, Fernández-Puente P, *et al.* Secretome analysis of human

For peer review only

For peer review only - http://bmjopen.bmj.com/site/about/guidelines.xhtml

articular chondrocytes unravels catabolic effects of nicotine on the joint. *Proteomics Clin Appl* 2016;**10**:671–80. doi:10.1002/prca.201400186

59 Fernández-Puente P, Calamia V, González-Rodríguez L, *et al.* Multiplexed mass spectrometry monitoring of biomarker candidates for osteoarthritis. *J Proteomics* 2017;**152**:216–25. doi:10.1016/j.jprot.2016.11.012

60 Lourido L, Ayoglu B, Fernández-Tajes J, *et al.* Discovery of circulating proteins associated to knee radiographic osteoarthritis. *Sci Rep* 2017;**7**:137. doi:10.1038/s41598-017-00195-8

61 Drobin K, Nilsson P, Schwenk JM. Highly multiplexed antibody suspension bead arrays for plasma protein profiling. *Methods Mol Biol* 2013;**1023**:137–45. doi:10.1007/978-1-4614-7209-4_8

62 Harris PA, Taylor R, Thielke R, *et al.* Research electronic data capture (REDCap)--a metadata-driven methodology and workflow process for providing translational research informatics support. *J Biomed Inform* 2009;**42**:377–81. doi:10.1016/j.jbi.2008.08.010

63 Harris PA, Taylor R, Minor BL, *et al.* The REDCap consortium: Building an international community of software platform partners. *J Biomed Inform* 2019;**95**:103208. doi:10.1016/j.jbi.2019.103208

64 Jaffe GJ, Dick AD, Brézin AP, *et al.* Adalimumab in Patients with Active Noninfectious Uveitis. *N Engl J Med* 2016;**375**:932–43. doi:10.1056/NEJMoa1509852

65 NCT01829295: Methotrexate and Mycophenolate Mofetil for UVEITIS (FAST). https://clinicaltrials.gov/ct2/show/study/NCT01829295?cond=Uveitis&draw=2&rank=35

66 Samson CM, Waheed N, Baltatzis S, *et al.* Methotrexate therapy for chronic

For peer review only - http://bmjopen.bmj.com/site/about/guidelines.xhtml

noninfectious uveitis: Analysis of a case series of 160 patients. *Ophthalmology* 2001;**108**:1134–9.

67 Galor A, Jabs DA, Leder HA, *et al.* Comparison of antimetabolite drugs as corticosteroid-sparing therapy for noninfectious ocular inflammation. *Ophthalmology* 2008;**115**:1826–32. doi:10.1016/j.ophtha.2008.04.026

68 Martínez C, Ortiz AM, Juarranz Y, *et al.* Serum levels of vasoactive intestinal peptide as a prognostic marker in early arthritis. *PLoS One* 2014;**9**:e85248. doi:10.1371/journal.pone.0085248

69 Abasolo L, Ivorra-Cortes J, Leon L, *et al.* Contribution of the bone and cartilage/soft tissue components of the joint damage to the level of disability in rheumatoid arthritis patients: a longitudinal study. *Clin Rheumatol* Published Online First: 16 October 2018. doi:10.1007/s10067-018-4335-4

70 Madrid-García A, Font-Urgelles J, Vega-Barbas M, *et al.* Outpatient Readmission in Rheumatology: A Machine Learning Predictive Model of Patient's Return to the Clinic. *J Clin Med* 2019;**8**. doi:10.3390/jcm8081156

For peer review only

FIGURE LEGENDS

Figure 1: Overview of the study timeline and interventions.

ACKNOWLEDGEMENTS

Sponsor

Fundación para la Investigacion Biomédica del Hospital Clínico San Carlos. The study sponsor had no role in the study design; collection, management, analysis and interpretation of data; writing of the report and the decision to submit the report for publication.

Committees

Executive Committee: Administrative and executive arm of the clinical trial, providing overall oversight for the study and making decisions on day-to-day operational issues [Study Coordinator (Luis Rodriguez-Rodriguez), a representative from the Spanish Clinical Trial Network (Amanda López Picado), and 5 Site Directors (these seats will be rotatory, with changes every 6 months)]; Data Coordinating and Analysis Committee: Supervising data collection,management and quality control, designing the statistical analysis plan, performing unmasked data analysis, and preparing interim and final reports for the Data Security Monitoring Board and the Executy Committee [Study Coordinator (Luis Rodriguez-Rodriguez), a representative from the Spanish Clinical

For peer review only - http://bmjopen.bmj.com/site/about/guidelines.xhtml

Trial Network (Amanda López Picado), and Ester Carreño]; Biobank and Biomarker Identification Committee (Maintaining an up-to-date manual of operations for blood extraction, processing and storage, and monitoring procedures adherence, supervising biological sample collection, sample shipment coordination, coordinating the phamacogenetic and proteomic analysis [Study Coordinator (Luis Rodriguez-Rodriguez), a representative from the Instituto de Salud Carlos III Biobank Platform (Elena Molino), a representative the Instituto de Investigación Biomédica de A Coruña, a representative from, the Data Coordinating and Analysis Committee]; Data Security Monitoring Committee (PierGiogio Neri, Andrew Dick, Loreto Carmona).

Patient consent for publication

Not required.

CoTHEIA Study Group

Mara Albert Fort (Hospital Universitario Doctor Peset), Mayte Ariño Gutierrez (Hospital Clínico San Carlos), Pedro Arriola Villalobos (Hospital Clínico San Carlos), Joseba Artaraz Beobide (Hospital Universitario Cruces), Antonio Atanes Sandoval (Complejo Hospitalario Universitario A Coruña), Jose Manuel Benitez Del Castillo Sanchez (Hospital Clínico San Carlos), Francisco Blázquez (Instituto Universitario de Oftalmología Aplicada, Universidad de Valladolid), Pablo Borges Deniz (Hospital Universitario Fundación Jiménez Díaz), Lara Borrego Sanz (Hospital Clínico San Carlos), Gabriela Bustamante Sanchez-Arnedo (Instituto Universitario de Oftalmología Aplicada, Universidad de Valladolid), Bruno Casco Silva (Complejo Hospitalario Universitario de Toledo), Ricardo Cuiña Sardiña (Hospital Clínico San Carlos), Almudena De Pablo Cabrera (Hospital Universitario 12 de Octubre), Maria Del Mar

For peer review only - http://bmjopen.bmj.com/site/about/guidelines.xhtml

Esteban Ortega (Hospital Universitario Infanta Sofía), Patricia Fernandez Puente (Instituto de Investigación Biomédica de A Coruña), Dalifer Dayanira Freites Nuñez (Hospital Clínico San Carlos), Javier Garcia Bella (Hospital Clínico San Carlos), Sara Garcia Carazo (Hospital Universitario La Paz), Luis Garcia Onrubia (Instituto Universitario de Oftalmología Aplicada, Universidad de Valladolid), Jose Maria Garcia Ruiz De Morales (Complejo Asistencial Universitario de León), Jose Antonio Gegundez Fernandez (Hospital Clínico San Carlos), Vanesa Hernandez Hernandez (Complejo Hospitalario Universitario de Canarias), Inés Hernanz Rodriguez (Hospital Universitario Fundación Jiménez Díaz), Jose Maria Herreras Cantalapiedra (Instituto Universitario de Oftalmología Aplicada, Universidad de Valladolid), Ventura Hidalgo Barrero (Hospital Universitario La Paz), Maria De La Vega Jovani Casano (Hospital General Universitario de Alicante), Maria Isabel Lopez Rodriguez (Complejo Hospitalario Universitario A Coruña), Sara López Sierra (Hospital Universitario Doctor Peset), Virginia Lozano Lopez (Complejo Hospitalario Universitario de Canarias), Alfredo Madrid Garcia (Hospital Clínico San Carlos), Rosalia Mendez Fernandez (Hospital Clínico San Carlos), Pilar Nozal Aranda (Hospital Universitario La Paz), Eugenio Perez Blazquez (Hospital Universitario 12 de Octubre), Chamaida Plasencia Rodriguez (Hospital Universitario La Paz), Sheila Recuero Diaz (Hospital Universitario Fundación Jiménez Díaz), Ignacio Robles Barrena (Hospital Universitario Rey Juan Carlos), Mª Esther Rodriguez Almaraz (Hospital Universitario 12 de Octubre), Fayna Maria Rodriguez Gonzalez (Hospital Universitario de Gran Canaria Doctor Negrín), Guadalupe Rodriguez Martinez (Complejo Hospitalario Universitario A Coruña), Fredeswinda Isabel Romero Bueno (Hospital Universitario Fundación Jiménez Díaz), Ioana Ruiz Arruza (Hospital Universitario Cruces), Beatriz Sanchez Marugan (Hospital Universitario Infanta Leonor), Olga Sanchez Pernaute (Hospital Universitario

For peer review only

For peer review only - http://bmjopen.bmj.com/site/about/guidelines.xhtml

Fundación Jiménez Díaz), Armelle Schlinker Giraud (Hospital Universitario La Paz), Alvaro Seijas Lopez (Complejo Hospitalario Universitario A Coruña), Patricia Simon Alonso (Complejo Hospitalario Universitario A Coruña), Marta Tejera Santana (Hospital Universitario de Gran Canaria Doctor Negrín), Elia Valls Pascual (Hospital Universitario Doctor Peset).

For peer review only

For peer review only - http://bmjopen.bmj.com/site/about/guidelines.xhtml

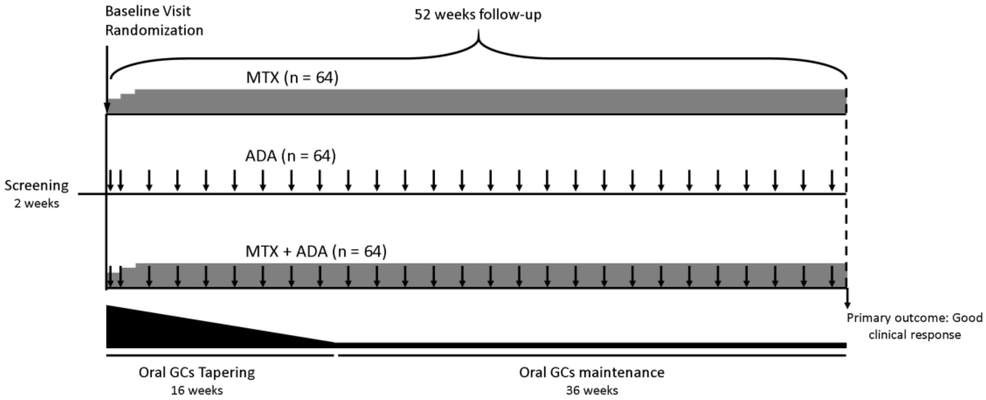

Overview of the study timeline and interventions

HOJA DE INFORMACIÓN AL PACIENTE

Solicitamos su participación en un proyecto de investigación promovido por investigadores de su centro y dirigido por el doctor Luis Rodriguez del Hospital Clínico San Carlos (Madrid). El estudio se titula **"Eficacia, seguridad y coste-efectividad del metotrexato, adalimumab, o su combinación en uveítis no anterior no infecciosa: un estudio multicéntrico, aleatorizado, paralelo de 3 brazos, con control activo, de fase 3, abierto, con evaluador cegado: Co-THEIA (CombinationTHerapy with mEthotrexate and adalImumAb for uveitis)"**

Siguiendo los estándares internacionalmente reconocidos y la normativa legal vigente en España sobre investigación, este ensayo clínico cumple todas las exigencias legales y ha sido aprobado por el Comité Ético de Investigación con medicamentos del Hospital Clínico San Carlos así como por la Agencia Española de Medicamentos y Productos Sanitarios.

Sin embargo, antes de aceptar participar o no, es importante que lea detenidamente la siguiente información y que realice todas las preguntas y aclare todas las cuestiones que crea conveniente con un familiar/amigo o con su médico.

¿Tengo que participar?

La participación en el estudio es completamente voluntaria. Rechazarla no acarreará ningún deterioro en la calidad de la asistencia ni en su atención médica. Además, podrá retirarse en cualquier momento del estudio sin tener que dar explicaciones y sin que ello repercuta en los

For peer review only - http://bmjopen.bmj.com/site/about/guidelines.xhtml

cuidados médicos que reciba. Su médico también podrá decidir, por alguna consideración médica o por cualquier otra razón, retirarle del estudio si así lo cree conveniente.

Así mismo, el hecho de que usted participe en este estudio no altera de ninguna manera ni sus tratamientos habituales ni el seguimiento de las enfermedades que tenga (en caso de que las tuviera).

¿En qué consiste el estudio y cómo se va a llevar a cabo?

Su médico le ha diagnosticado una uveítis no infecciosa, una inflamación de una parte del ojo que no está causada por un microorganismo (bacteria, hongo o virus).

Para tratar esta inflamación es posible que su oftalmólogo y/o reumatólogo/inmunólogo/especialista en Medicina Interna ya le haya pautado distintos tratamientos, principalmente corticoides. Sin embargo, estos fármacos a veces no son suficientes para controlar enfermedad. Por otro lado, es posible que haya sido diagnosticado/a de algún tipo de uveitis que se asocie a una inflamación más persistente. En ambos casos, en la actualidad disponemos de distintos medicamentos para el tratamiento pero solo tiene indicación aprobada el adalimumab. En este estudio vamos a comparar este medicamento y el metotrexato, solos o combinados.

El metotrexato es un fármaco que inhibe el sistema inmunitario y esto permite reducir la inflación del ojo. Se lleva utilizando desde hace más de 50 años en el tratamiento de otras enfermedades inflamatorias crónicas y más reccientemente (más de 30 años) en el tratamiento

For peer review only - http://bmjopen.bmj.com/site/about/guidelines.xhtml

de las uveitis. Se puede tomar en pastillas o inyectado en la piel (vía subcutánea), su médico según su caso le indicara como y cuando tomarlo.

El adalimumab es otro medicamente que también inhibe el sistema inmunitario y reduce la inflamación, pero utiliza otro mecanismo de acción. Se lleva utilizando en el tratamiento de las uveitis desde hace más de 10 años, administrándose por vía subcutánea.

Si usted decide participar en el estudio podrá ser asignado a uno de los tres grupos en estudio: metotrexato, adalimumab y ambos (metotrexato+adalimumab). La asignación será al azar, es decir ni usted ni su médico decidirá que tratamiento recibe. Indistintamente el grupo al que sea asignado, las visitas y las pruebas que se le realicen serán las que habitualmente se realizan a pacientes como usted. Además, en el caso de que haya sido diagnosticado de algunos subtipos de uveítis (como es la uveítis intermedia), se le realizará una resonancia magnética cerebral con gadolinio antes de iniciar el estudio. Esto es debido a que estos subtipos de uveitis podrían estar asociados a la presencia de enfermedades desmielinizantes del sistema nervioso central, en las que el uso de adalimumab se encuentra contraindicado.

For peer review only

Usted tendrá acudir cada 4 semanas al centro. En estas visitas su médico se encargará revisar la situación del ojo (examen del ojo, agudeza visual…), los eventos adversos y la adherencia al tratamiento. Además, deberá completar algunos cuestionarios para evaluar su calidad de vida, los costes y los fármacos que toma (aproximadamente 15 minutos). Para completar estos cuestionarios dispondrá de ayuda del personal del centro.

For peer review only - http://bmjopen.bmj.com/site/about/guidelines.xhtml

Adicionalmente le solicitaremos permiso para la extracción de unas muestras de sangre al inicio, tras 16 semanas, cuando finalice y en el caso de que tuviera algún brote de la uveítis durante el estudio. Estas muestras serán utilizadas para hacer análisis genéticos y de proteómica para identificar marcadores que permitan conocer más su enfermedad y /o que puedan estar asociados a una mejor respuesta al tratamiento. También, en el caso de que fuera a recibir adalimumab, para medir los niveles en sangre de ese fármaco. Por último, parte de estas muestras serán conservadas en la Colección de Muestras para la investigación en enfermedades reumatológicas del Servicio de Reumatología del Hospital Clínico San Carlos, con el objetivo de llevar a cabo futuros estudios que nos ayuden a conocer mejor la enfermedad que usted padece. Estas muestras solo serán conservadas y utilizadas en estudios futuros en caso de que usted de consentimiento informado expreso.

¿Qué beneficios puedo esperar por el hecho de participar en el estudio?

Puede ocurrir que usted no obtenga ningún beneficio personal de la participación en este proyecto de investigación. En cualquier caso, su participación en este estudio contribuirá al avance del conocimiento sobre el tratamiento de la uveítis no infecciosa.

¿Qué incomodidades o riesgos puede suponer mi participación en el estudio?

Entre los efectos secundarios más habituales del metotrexato están el dolor de cabeza, los moratones y sangrados, el aumento de las infecciones, las náuseas y vómitos, la diarrea, los dolores musculares, el cansancio, la pérdida de cabello y la afectación hepática.

For peer review only - http://bmjopen.bmj.com/site/about/guidelines.xhtml

Por su parte, el adalimumab presenta los siguientes efectos secundarios: molestias o dolor en el punto de inyección, erupción cutánea, dolor de cabeza e infecciones (siendo las más habituales las de las vías respiratorias altas (catarros entre otros) y la sinusitis).

En el caso de administrar de manera conjunta ambos fármacos los efectos adversos podrían ser más intensos y aparecer algunos desconocidos. En este momento, la administración de estos dos medicamentos de manera conjunta no esta autorizada.

En el caso de que se le tuviese que realizar una resonancia nuclear magnética con gadolinio, el uso de este contraste puede verse asociado a eventos adverso agudos en 1 de cada 1000 individuos, siendo los más frecuentes de la aparición de rash, urticaria y nauseas. Además, ha sido descrita la posibilidad de depósito de esta substancia a nivel cerebral y en otros tegidos, tras administraciones repetidas.

¿Cuáles son mis derechos y cómo van a ser tratados mis datos?

Toda la información que se registrará de usted será estrictamente confidencial. De acuerdo con el Reglamento General de Protección de Datos (RGPD) (Reglamento (EU) 2016/679), además de los derechos de acceso, rectificación, oposición y cancelación de datos (Ley Orgánica 3/2018, de 5 de diciembre, de Protección de Datos Personales y garantía de los derechos digitales), también tiene derecho a limitar el tratamiento de datos y solicitar una copia o que se trasladen a un tercero (portabilidad) los datos que usted ha facilitado para el estudio. Para ejercitar sus derechos, diríjase al investigador principal del estudio. No obstante, le recordamos que los datos no se pueden eliminar, aunque deje de participar en el ensayo para garantizar la validez de la investigación y cumplir con los deberes legales y los requisitos de autorización de medicamentos. Así mismo tiene derecho a dirigirse a la Agencia de Protección de Datos si no quedara satisfecho.

For peer review only

For peer review only - http://bmjopen.bmj.com/site/about/guidelines.xhtml

En todo momento se mantendrá el anonimato de los pacientes y, para ello, en ninguno de los documentos del estudio aparecerá su nombre, sino que le será asignado un número que será el que se utilice en todos ellos. En todos los informes escritos y publicaciones, sólo aparecerá su número de referencia. Sólo el médico responsable del estudio guardará, en condiciones de seguridad, la lista que relaciona los nombres de los pacientes con los números de referencia asignados a cada uno.

Sólo tendrán acceso a los datos del estudio el equipo investigador y el monitor del estudio, miembros del comité, autoridades sanitarias competentes y/o de la agencia reguladora (AEMPS) para asegurar que el estudio se está llevando a cabo con las leyes vigentes y la reglamentación sanitaria. Firmando este documento, usted está autorizando este acceso. Además, los resultados del estudio siempre serán presentados de manera global y nunca de forma individualizada.

El Investigador y el Promotor están obligados a conservar los datos recogidos para el estudio al menos hasta 25 años tras su finalización. Posteriormente, su información personal solo se conservará por el centro para el cuidado de su salud y por el promotor para otros fines de investigación científica si usted hubiera otorgado su consentimiento para ello, y si así lo permite la ley y requisitos éticos aplicables.

Si realizáramos transferencia de sus datos codificados fuera de la UE a las entidades de nuestro grupo, a prestadores de servicios o a investigadores científicos que colaboren con nosotros, los

For peer review only - http://bmjopen.bmj.com/site/about/guidelines.xhtml

datos del participante quedarán protegidos con salvaguardas tales como contratos u otros mecanismos por las autoridades de protección de datos. Si el participante quiere saber más al respecto, puede contactar al Delegado de Protección de Datos del promotor o Investigador Principal.

¿Mi participación supondrá algún coste o compensación económica?

Su participación en este estudio no supondrá para usted ningún coste económico, así como tampoco será recompensado económicamente por ello.

estudio dispone de una póliza de seguros que se ajusta a la legislación vigente (Real decreto 1090/2015) y que le proporcionará la compensación e indemnización en caso de menoscabo de su salud o de lesiones que pudieran producirse en relación con su participación en el estudio, siempre que no sean consecuencia de la propia enfermedad que se estudia o de la evolución propia de su enfermedad como consecuencia de la ineficacia del tratamiento. Este estudio se considera de Bajo Nivel de Intervención (estudios de bajo riesgo, muy parecidos a la práctica clinica habitual), por lo que la póliza con la que esta usted cubierto es la póliza de Sistema de Salud.

Si usted tiene alguna duda o quiere más información, no dude en consultar con el médico responsable que le está solicitando este consentimiento.

For peer review only - http://bmjopen.bmj.com/site/about/guidelines.xhtml

¿Cómo obtener información adicional?

Si desea información adicional sobre el estudio puede contactar con:

____

GRACIAS POR LEER ESTA INFORMACIÓN

For peer review only

For peer review only - http://bmjopen.bmj.com/site/about/guidelines.xhtml

Código Protocolo: Co-THEIA Versión: 2
Fecha: 11 de septiembre de 2020

CONSENTIMIENTO INFORMADO PARA LOS PACIENTES

Título del estudio: **"Eficacia, seguridad y coste-efectividad del metotrexato, adalimumab, o su combinación en uveítis no anterior no infecciosa: un estudio multicéntrico, aleatorizado, paralelo de 3 brazos, con control activo, de fase 3, abierto, con evaluador cegado: Co-THEIA (CombinationTHerapy with mEthotrexate and adalImumAb for uveitis)"**

Yo…………………………………………………………………………..

(Nombre y apellidos del participante, puño y letra del paciente)

DECLARO:

- Que he hablado con ……………………………………………..

(Nombre del médico, puño y letra del paciente)

- Que he leído la hoja de información que se me ha entregado sobre el estudio.
- He comprendido la información recibida y he podido formular todas las preguntas que he creído oportunas.
- Comprendo que mi participación es voluntaria y que en cualquier momento puedo revocar mi consentimiento sin tener que dar explicaciones y sin que afecte en ningún aspecto a mi relación con el personal médico ni a la atención recibida por su parte.
- He recibido una copia firmada y fechada de este Consentimiento Informado.

☐ **Autorizo la conservación de muestras biológicas obtenidas y su uso para estudios futuros**

EN CONSECUENCIA, DOY MI CONSENTIMIENTO PARA FORMAR PARTE DE ESTE PROYECTO DE INVESTIGACION.

Firma del paciente	Firma del médico responsable
……………………………………	…………………………………
Nombre y apellidos	Nombre y apellidos
Fecha…………………………	Fecha………………………..

For peer review only - http://bmjopen.bmj.com/site/about/guidelines.xhtml

Reporting checklist for protocol of a clinical trial.

Based on the SPIRIT guidelines.

Instructions to authors

Complete this checklist by entering the page numbers from your manuscript where readers will find each of the items listed below.

Your article may not currently address all the items on the checklist. Please modify your text to include the missing information. If you are certain that an item does not apply, please write "n/a" and provide a short explanation.

Upload your completed checklist as an extra file when you submit to a journal.

In your methods section, say that you used the SPIRITreporting guidelines, and cite them as:

Chan A-W, Tetzlaff JM, Gøtzsche PC, Altman DG, Mann H, Berlin J, Dickersin K, Hróbjartsson A, Schulz KF, Parulekar WR, Krleža-Jerić K, Laupacis A, Moher D. SPIRIT 2013 Explanation and Elaboration: Guidance for protocols of clinical trials. BMJ. 2013;346:e7586

		Reporting Item	Page Number
**Administrative information**			
Title	#1	Descriptive title identifying the study design, population, interventions, and, if applicable, trial acronym	1
Trial registration	#2a	Trial identifier and registry name. If not yet registered, name of intended registry	21
Trial registration: data set	#2b	All items from the World Health Organization Trial Registration Data Set	22
Protocol version	#3	Date and version identifier	21
Funding	#4	Sources and types of financial, material, and other support	32
Roles and responsibilities: contributorship	#5a	Names, affiliations, and roles of protocol contributors	32

For peer review only

Roles and responsibilities: sponsor contact information	#5b	Name and contact information for the trial sponsor	33
Roles and responsibilities: sponsor and funder	#5c	Role of study sponsor and funders, if any, in study design; collection, management, analysis, and interpretation of data; writing of the report; and the decision to submit the report for publication, including whether they will have ultimate authority over any of these activities	33
Roles and responsibilities: committees	#5d	Composition, roles, and responsibilities of the coordinating centre, steering committee, endpoint adjudication committee, data management team, and other individuals or groups overseeing the trial, if applicable (see Item 21a for data monitoring committee)	33
**Introduction**			
Background and rationale	#6a	Description of research question and justification for undertaking the trial, including summary of relevant studies (published and unpublished) examining benefits and harms for each intervention	7
Background and rationale: choice of comparators	#6b	Explanation for choice of comparators	6
Objectives	#7	Specific objectives or hypotheses	9
Trial design	#8	Description of trial design including type of trial (eg, parallel group, crossover, factorial, single group), allocation ratio, and framework (eg, superiority, equivalence, non-inferiority, exploratory)	9
**Methods: Participants, interventions, and outcomes**			
Study setting	#9	Description of study settings (eg, community clinic, academic hospital) and list of countries where data will be collected. Reference to where list of study sites can be obtained	11
Eligibility criteria	#10	Inclusion and exclusion criteria for participants. If applicable, eligibility criteria for study centres and individuals who will	11

For peer review only

		perform the interventions (eg, surgeons, psychotherapists)	
Interventions: description	#11a	Interventions for each group with sufficient detail to allow replication, including how and when they will be administered	12
Interventions: modifications	#11b	Criteria for discontinuing or modifying allocated interventions for a given trial participant (eg, drug dose change in response to harms, participant request, or improving / worsening disease)	15
Interventions: adherance	#11c	Strategies to improve adherence to intervention protocols, and any procedures for monitoring adherence (eg, drug tablet return; laboratory tests)	17
Interventions: concomitant care	#11d	Relevant concomitant care and interventions that are permitted or prohibited during the trial	12
Outcomes	#12	Primary, secondary, and other outcomes, including the specific measurement variable (eg, systolic blood pressure), analysis metric (eg, change from baseline, final value, time to event), method of aggregation (eg, median, proportion), and time point for each outcome. Explanation of the clinical relevance of chosen efficacy and harm outcomes is strongly recommended	9
Participant timeline	#13	Time schedule of enrolment, interventions (including any run-ins and washouts), assessments, and visits for participants. A schematic diagram is highly recommended (see Figure)	15
Sample size	#14	Estimated number of participants needed to achieve study objectives and how it was determined, including clinical and statistical assumptions supporting any sample size calculations	17
Recruitment	#15	Strategies for achieving adequate participant enrolment to reach target sample size	11
**Methods: Assignment of interventions (for controlled trials)**			
Allocation: sequence generation	#16a	Method of generating the allocation sequence (eg, computer-generated random numbers), and list of any factors for stratification. To reduce predictability of a random sequence, details of any planned restriction (eg, blocking) should be provided in a separate document that is unavailable to those who enrol participants or assign interventions	12

For peer review only

Allocation concealment mechanism	#16b	Mechanism of implementing the allocation sequence (eg, central telephone; sequentially numbered, opaque, sealed envelopes), describing any steps to conceal the sequence until interventions are assigned	12
Allocation: implementation	#16c	Who will generate the allocation sequence, who will enrol participants, and who will assign participants to interventions	12
Blinding (masking)	#17a	Who will be blinded after assignment to interventions (eg, trial participants, care providers, outcome assessors, data analysts), and how	13
Blinding (masking): emergency unblinding	#17b	If blinded, circumstances under which unblinding is permissible, and procedure for revealing a participant's allocated intervention during the trial	13
**Methods: Data collection, management, and analysis**			
Data collection plan	#18a	Plans for assessment and collection of outcome, baseline, and other trial data, including any related processes to promote data quality (eg, duplicate measurements, training of assessors) and a description of study instruments (eg, questionnaires, laboratory tests) along with their reliability and validity, if known. Reference to where data collection forms can be found, if not in the protocol	10
Data collection plan: retention	#18b	Plans to promote participant retention and complete follow-up, including list of any outcome data to be collected for participants who discontinue or deviate from intervention protocols	11
Data management	#19	Plans for data entry, coding, security, and storage, including any related processes to promote data quality (eg, double data entry; range checks for data values). Reference to where details of data management procedures can be found, if not in the protocol	15
Statistics: outcomes	#20a	Statistical methods for analysing primary and secondary outcomes. Reference to where other details of the statistical analysis plan can be found, if not in the protocol	18
Statistics: additional analyses	#20b	Methods for any additional analyses (eg, subgroup and adjusted analyses)	18

For peer review only

Statistics: analysis population and missing data	#20c	Definition of analysis population relating to protocol non-adherence (eg, as randomised analysis), and any statistical methods to handle missing data (eg, multiple imputation)	17
**Methods: Monitoring**			
Data monitoring: formal committee	#21a	Composition of data monitoring committee (DMC); summary of its role and reporting structure; statement of whether it is independent from the sponsor and competing interests; and reference to where further details about its charter can be found, if not in the protocol. Alternatively, an explanation of why a DMC is not needed	16
Data monitoring: interim analysis	#21b	Description of any interim analyses and stopping guidelines, including who will have access to these interim results and make the final decision to terminate the trial	18
Harms	#22	Plans for collecting, assessing, reporting, and managing solicited and spontaneously reported adverse events and other unintended effects of trial interventions or trial conduct	15
Auditing	#23	Frequency and procedures for auditing trial conduct, if any, and whether the process will be independent from investigators and the sponsor	15
**Ethics and dissemination**			
Research ethics approval	#24	Plans for seeking research ethics committee / institutional review board (REC / IRB) approval	21
Protocol amendments	#25	Plans for communicating important protocol modifications (eg, changes to eligibility criteria, outcomes, analyses) to relevant parties (eg, investigators, REC / IRBs, trial participants, trial registries, journals, regulators)	21
Consent or assent	#26a	Who will obtain informed consent or assent from potential trial participants or authorised surrogates, and how (see Item 32)	21
Consent or assent: ancillary studies	#26b	Additional consent provisions for collection and use of participant data and biological specimens in ancillary studies, if applicable	22
Confidentiality	#27	How personal information about potential and enrolled participants will be collected, shared, and maintained in order to protect confidentiality before, during, and after the trial	22

For peer review only

Declaration of interests	#28	Financial and other competing interests for principal investigators for the overall trial and each study site	32
Data access	#29	Statement of who will have access to the final trial dataset, and disclosure of contractual agreements that limit such access for investigators	33
Ancillary and post trial care	#30	Provisions, if any, for ancillary and post-trial care, and for compensation to those who suffer harm from trial participation	15
Dissemination policy: trial results	#31a	Plans for investigators and sponsor to communicate trial results to participants, healthcare professionals, the public, and other relevant groups (eg, via publication, reporting in results databases, or other data sharing arrangements), including any publication restrictions	22
Dissemination policy: authorship	#31b	Authorship eligibility guidelines and any intended use of professional writers	32
Dissemination policy: reproducible research	#31c	Plans, if any, for granting public access to the full protocol, participant-level dataset, and statistical code	22
**Appendices**			
Informed consent materials	#32	Model consent form and other related documentation given to participants and authorised surrogates	14
Biological specimens	#33	Plans for collection, laboratory evaluation, and storage of biological specimens for genetic or molecular analysis in the current trial and for future use in ancillary studies, if applicable	10

The SPIRIT Explanation and Elaboration paper is distributed under the terms of the Creative Commons Attribution License CC-BY-NC. This checklist was completed on 16. March 2021 using https://www.goodreports.org/, a tool made by the EQUATOR Network in collaboration with Penelope.ai

For peer review only

BMJ Open

Efficacy, safety and cost-effectiveness of methotrexate, adalimumab, or their combination in non-infectious non-anterior uveitis: a protocol for a multicenter, randomized, parallel 3 arms, active-controlled, phase 3 open label with blinded outcome assessment study

Journal:	*BMJ Open*
Manuscript ID	bmjopen-2021-051378.R2
Article Type:	Protocol
Date Submitted by the Author:	04-Feb-2022
Complete List of Authors:	Rivas, Ana; La Fundacion para la Investigacion Biomedica del Hospital Clinico San Carlos<br>Lopez-Picado, Amanda; La Fundacion para la Investigacion Biomedica del Hospital Clinico San Carlos<br>Calamia, Valentina; Instituto de Investigación Biomédica de A Coruña<br>Carreño, Ester; Hospital Universitario Fundacion Jimenez Diaz<br>Cocho, Lidia; Institute of Applied Ophtalmobiology<br>Cordero-Coma, Miguel; University Hospital Centre León<br>Fonollosa, Alex; Cruces University Hospital<br>Francisco Hernandez, Felix; Hospital Universitario de Gran Canaria Dr Negrin<br>Garcia-Aparicio, Angel; Hospital Universitario de Toledo<br>Garcia-Gonzalez, Javier; Hospital Universitario 12 de Octubre<br>Mondejar, Jose Juan; Alicante General University Hospital<br>Lojo-Oliveira, Leticia; Hospital Universitario Infanta Leonor<br>Martínez-Costa, Llucí; Hospital Universitario Doctor Peset, Ophthalmology<br>Munoz, Santiago; Hospital Universitario Infanta Sofia<br>Peiteado, Diana; Hospital Universitario La Paz<br>Pinto, Jose Antonio; Complexo Hospitalario Universitario A Coruña Servizo de Pediatría<br>Rodriguez-Lozano, Beatriz; Hospital Universitario de Canarias<br>Pato, Esperanza; Hospital Clínico San Carlos<br>Diaz-Valle, David; Hospital Clínico San Carlos<br>Molina, Elena; La Fundacion para la Investigacion Biomedica del Hospital Clinico San Carlos<br>Tebar, Luis; La Fundacion para la Investigacion Biomedica del Hospital Clinico San Carlos<br>Rodriguez-Rodriguez, Luis; La Fundacion para la Investigacion Biomedica del Hospital Clinico San Carlos,
**Primary Subject Heading**:	Ophthalmology
Secondary Subject Heading:	Pharmacology and therapeutics
Keywords:	Clinical trials < THERAPEUTICS, OPHTHALMOLOGY, RHEUMATOLOGY

SCHOLARONE™
Manuscripts

Efficacy, safety and cost-effectiveness of methotrexate, adalimumab, or their combination in non-infectious non-anterior uveitis: a protocol for a multicenter, randomized, parallel 3 arms, active-controlled, phase 3 open label with blinded outcome assessment study

Ana Rivas[1,2], Amanda López Picado[1], Valentina Calamia[3], Ester Carreño[4], Lidia Cocho[5], Miguel Cordero-Coma[6], Alex Fonollosa[7], Felix M Francisco Hernandez[8], Angel Garcia Aparicio[9], Javier Garcia Gonzalez[10], Jose Juan Mondejar[11], Leticia Lojo Oliveira[12], Llucí Martinez-Costa[13], Santiago Muñoz[14], Diana Peiteado[15], Jose Antonio Pinto[16], Beatriz Rodriguez Lozano[17], Esperanza Pato[18], David Diaz-Valle[19], Elena Molina[20], Luis Tebar[1], Luis Rodriguez-Rodriguez[21], CoTHEIA Study Group.

[1] Unidad de Investigación Clinica y Ensayos Clínicos. Hospital Clínico San Carlos, IdISSC, Madrid (Spain), [2] Departamento de Enfermería. Facultad Enfermería, Fisioterapia y Podología. Universidad Complutense de Madrid, Madrid (Spain), [3]Unidad de Proteómica. Grupo de Investigación de Reumatología (GIR). Instituto de Investigación Biomédica de A Coruña (INIBIC), Complexo Hospitalario Universitario de A Coruña (CHUAC), and Universidade da Coruña (UDC), A Coruña (Spain), [4]University Hospital Fundacion Jimenez Diaz, and University Hospital Rey Juan Carlos, Madrid (Spain), [5]IOBA (Institute of Applied OphthalmoBiology), University of Valladolid, and Hospital Clínico Universitario de Valladolid, Valladolid (Spain), [6]Uveitis Unit. University Hospital of León, IBIOMED, and University of León, Leon (Spain), [7]Department of Ophthalmology, BioCruces Bizkaia Health Research Institute, Cruces University Hospital, University of the Basque Country, Barakaldo, Spain, [8]Rheumatology Department, Hospital Universitario de Gran Canaria Doctor Negrín, Las

For peer review only - http://bmjopen.bmj.com/site/about/guidelines.xhtml

Palmas de Gran Canaria (Spain), [9]Department of Rheumatology, Hospital Universitario de Toledo, Toledo (Spain), [10]Department of Rheumatology, Hospital Universitario 12 de Octubre, Madrid (Spain), [11]Ophthalmology Department, Hospital General Universitario de Alicante, Alicante (Spain), [12]Sección de Reumatología, Hospital Universitario Infanta Leonor, Madrid (Spain), [13]Ophthalmology Department, Hospital Universitario Doctor Peset, Valencia (Spain), [14]Rheumatology Department, Hospital Universitario Infanta Sofia, San Sebastián de los Reyes (Spain), [15]Rheumatology Unit. Hospital Universitario La Paz, Madrid (Spain), [16]Rheumatology Department, Complexo Hospitalario Universitario de A Coruña (CHUAC), A Coruña (Spain), [17]Rheumatology Department, Hospital Universitario de Canarias, Santa Cruz de Tenerife (Spain), [18]Rheumatology Department, Hospital Clínico San Carlos, IdISSC, Madrid (Spain), [19]Servicio de Oftalmología, Hospital Clínico San Carlos. Departamento de Inmunología, Oftalmología y ORL, Facultad de Medicina, Universidad Complutense de Madrid, IdISSC, Madrid (Spain), [20]Department of Pathology, Hospital Clínico San Carlos, IdISSC, Madrid (Spain), [21]Musculoskeletal Pathology Group, Rheumatology Department, Hospital Clínico San Carlos, IdISSC, Madrid (Spain)

Correspondence

Dr Luis Rodriguez-Rodriguez, Hospital Clínico San Carlos, c\ Prof. Martín Lagos s/n, 28040, Madrid (Spain); Phone: +34913303615; email:lrrodriguez@salud.madrid.org

Word Count: 4,678

For peer review only - http://bmjopen.bmj.com/site/about/guidelines.xhtml

ABSTRACT

Introduction: Non-infectious uveitis (NIUs) include a heterogeneous group of sight-threatening and incapacitating conditions. Their correct management sometimes requires the use of immunosuppressive drugs (ISDs), prescribed in monotherapy or in combination. Several observational studies showed that the use of ISDs in combination could be more effective than and as safe as their use in monotherapy. However, a direct comparison between these two treatment strategies has not been carried out yet.

Methods and analysis: The Combination THerapy with mEthotrexate and adalImumAb for uveitis (CoTHEIA) study is a phase III, multicenter, prospective, randomized, single-blinded with masked outcome assessment, parallel three arms with 1:1:1 allocation, active-controlled, superiority study design, comparing the efficacy, safety and cost-effectiveness of methotrexate (MTX), adalimumab (ADA), or their combination in non-infectious non-anterior uveitis. We aim to recruit 192 subjects. The duration of the treatment and follow-up will last up to 52 weeks, plus 70 days follow-up with no treatment. The complete and maintained resolution of the ocular inflammation will be assessed by masked evaluators (primary outcome). In addition to other secondary measurements of efficacy (QoL, visual acuity, costs) and safety, we will identify subjects' subgroups with different treatment responses by developing prediction models based on machine learning techniques using genetic and proteomic biomarkers.

Ethics and dissemination: The protocol, annexes and informed consent forms were approved by the Reference Clinical Research Ethic Committee at the Hospital Clínico San Carlos (Madrid, Spain) and the Spanish Agency for Medicines and Health Products (AEMPS). We will elaborate a Dissemination plan including production of materials adapted to several formats to communicate the clinical trial progress and findings to a

For peer review only - http://bmjopen.bmj.com/site/about/guidelines.xhtml

broad group of stakeholders. The promoter will be the only with access to the participant-level data, although it can be shared within the legal situation.

Registration details: clinicaltrialsregister.eu (2020-000130-18) and clinicaltrials.gov (NCT04798755).

Keywords: Uveitis, adalimumab, methotrexate, combination therapy, randomised clinical trial

For peer review only

ARTICLE SUMMARY

Strengths and Limitations of this Study

1. This is the first randomized controlled study designed to compare the efficacy of combination therapy versus monotherapy for the treatment of non-infectious uveitis in subjects with no previous immunosuppressive treatment.

2. We have chosen quite a strict outcome (the requirement of a maintained controlled inflammation), more likely related to long-term outcomes (such as structural damage), and to patient reported outcomes measures.

3. Despite the previous point, our primary efficacy outcome is still a surrogate marker: the achievement of this outcome does not have necessarily to translate in an improvement of outcomes more important for the patient, such as quality of life or disability.

4. The requirement to control the inflammatory process early (by week 16) may cause an underestimation of drug efficacy, as it could take more time to control the inflammation but be associated with a similar long-term prognosis.

5.The lack of masking could introduce bias, although treatment characteristics and proven effectivity in non-infectious uveitis, the duration of the trial (up to 52 weeks), and the need for bi-weekly subcutaneous injections of one of the drugs (ADA), we consider unpractical for the subject the use of placebo in the present trial.

For peer review only - http://bmjopen.bmj.com/site/about/guidelines.xhtml

INTRODUCTION

Uveitides are potentially sight-threatening diseases[1]:worldwide, they represented up to 10% of causes of blindness (almost 4 million people)[2]. Furthermore, in the European Union and the United States, after diabetic retinopathy, uveitides represent the second major treatable cause of blindness in those 20-65 years of age[3](up to 10% of cases of blind registrations[3–6]). Additionally, a high percentage of patients suffer from uveitis-related complications, visual impairment[4,7,8], and a negative impact in quality-of-life (QoL)[9,10]. Considering their higher prevalence in young to middle-aged adults[11,12] uveitides cause an important economic, social, and personal burden[5,13–16].

The correct management of NIUs is essential for preserving visual function and avoiding ocular and extra-ocular morbidity[17]. Although glucocorticoids (GCs) are the mainstay of treatment[18], under certain circumstances, adding immunosuppressive drugs (ISDs) is needed to achieve a sustained control of the inflammatory process[19]. Several ISDs are used in the standard of care, such as methotrexate (MTX), and biological agents[19–21].

Regarding MTX, its effectiveness in NIU has been assessed in two randomized clinical trials, compared with mycophenolate mofetil (MMF): Rathinam et al[22] observed that 69% and 47% of patients treated with MTX and MMF, respectively, achieved complete control of inflammation and daily oral GCs dosage ≤10mg at 5 and 6 months ($p=0.09$). In a second trial[23], randomizing 200 patients, the percentage of subjects achieving a similar outcome was 67% and 57% for MTX and MMF, respectively ($p>0.05$), with similar tolerability. Regarding safety, adverse events (AEs) are generally mild and

For peer review only - http://bmjopen.bmj.com/site/about/guidelines.xhtml

discontinuations due to serious adverse events (SAEs) are less common than for most ISDs[24][25], translating it in higher retention rates[26,27].

Regarding ADA, its effectiveness in NIU has been shown in two randomized controlled trials[28,29]. The VISUAL I study[28] compared ADA with placebo in 217 NIU patients with active uveitis despite ≥10 mg/day of systemic GCs. Based on the cumulative number of subjects with treatment failure in each visit, by week 25 (about 6 months), 31 of 107 (29%) subjects in the placebo group and 62 of 110 (56%) subjects in the ADA group had not suffered a treatment failure. By 50 weeks, the numbers were reduced to 24 (22%) and 51 (46%), respectively. Regarding safety, many of the AEs are sufficiently mild to not require discontinuation, such as injection site pain, and anti-drug antibodies formation[19,30,31], reflecting in a high retention rate[32]. Regarding SAEs, there was no association with higher risk in a recent meta-analysis[33], compared with placebo or synthetic ISDs. However, ADA was associated to a higher risk of treatment discontinuation due to SAEs. One of the most important ADA's AEs are infections. However, a previous meta-analysis reported that the absolute risk was low (0.036% with TNF-alpha inhibitors vs. 0.017% with placebo)[34], which probably does not represent a clinically important constraint on the use of these agents. Finally, ADA has not showed an association with a higher risk of malignancy[33].

Although ISDs are usually used in monotherapy in uveitis, several observational studies have shown that in 21 to 52% percentage of NIU patients, the use of ISDs in monotherapy was unable to achieve a sustained control of the inflammatory process[27,35–37].Furthermore, other studies have provided evidence that the combination of 2 or more ISDs could offer advantages in terms of effectiveness and tolerability[38–41], in conditions such as Birdshot retinochoroidopathy[38], serpiginous

For peer review only - http://bmjopen.bmj.com/site/about/guidelines.xhtml

choroiditis[39,40], Vogt-Koyanagi-Harada syndrome (VHK)[42,43], ocular Behçet's disease[44,45], JIA associated uveitis[46], sympathetic ophthalmia[47] and intermediate uveitis[48]. Regarding the beneficial of combining both MTX and ADA, several RCTs have provided evidence in other immune mediated inflammatory diseases (IMIDs)[49]. In NIU, this combinations was tested in children with Juvenile Idiopathic Arthritis-associated Uveitis and a previous failure to MTX monotherapy [50]. The combination of MTX and ADA was more effective compared to MTX and placebo, although it was associated with a higher proportion of AEs (88% vs. 83% of patients) and SAEs (22% vs. 7%).

Despite all the evidence, a direct comparison between combination and monotherapy has not been tested yet, although some groups have adopted the use of combination therapy as the initial ISD treatment for particular conditions[51].

There are currently no tools able to predict the response or non-response to ISDs in those NIUs needing immunosuppression, as there is marked interpersonal variation in their efficacy and toxicity. Response to the first-line ISD treatment could be an important predictor of long-term outcomes, as the continuous or repeated eye inflammation could increase the risk of structural permanent damage, leading to blindness, disability, and deterioration in the QoL. Therefore, starting on the right ISD is likely a key factor in achieving a better and more cost-effective therapy, and improving the optimal allocation of health care resources. To achieve these aims, objectively measured and evaluated characteristics (biomarkers[52]) are required, in addition to the identification of clinical features associated with the outcomes of interest.

For peer review only - http://bmjopen.bmj.com/site/about/guidelines.xhtml

METHODS AND ANALYSIS

Study overview

The Combination THerapy with mEthotrexate and adalImumAb for uveitis (CoTHEIA) study is a phase III, multicentre, prospective, randomized, single-blinded with masked outcome assessment, parallel 3 arms with 1:1:1 allocation, active-controlled, superiority study design, comparing the efficacy, safety and cost-effectiveness of MTX, ADA, or their combination in non-infectious non-anterior uveitis (NINAU). The duration of the treatment and follow-up will last up to 52 weeks. A 70-day follow-up clinic visit or phone call will take place to assess safety after the last study drug dose. **Figure 1** provides an overview of the study timeline and interventions.

Objectives and Outcome Variables

The trial main hypothesis is that the use of combination therapy with MTX and ADA will be more effective in inducing and maintaining ocular inflammatory inactivity than either drug given in monotherapy.

The *primary efficacy objective* is to establish which treatment strategy results in a higher proportion of subjects achieving a complete and maintained resolution of the ocular inflammation, on an intent to treat basis. The *primary efficacy outcome* will be the proportion of patients achieving a **Good Clinical Response** between the combination therapy arm and the single immunosuppressive drugs arms. This outcome is defined as a complete resolution of the ocular inflammatory signs (including active chorioretinal lesions, active retinal vascular inflammation, uveitis macular oedema, presence of anterior chamber cells, and presence of vitreous haze), achieved within the

For peer review only - http://bmjopen.bmj.com/site/about/guidelines.xhtml

first 16 weeks of the study, and maintained during follow-up until the end of the study (week 52); furthermore, there must not be a treatment failure due to safety or intolerability; the subject must adhere to the initial (up to week 16) oral GCs tapering protocol; all study visits from Baseline to 16 weeks must be completed; and at the final visit (week 52), the subject must be treated with up to 7.5mg/day of oral prednisone (or equivalent) and up to 2 times a day of prednisolone acetate 1% (or equivalent).The *secondary efficacy objectives* and outcomes variables can be found at **Table 1**.

Table 1: Efficacy-related secondary objectives and outcome variables.

Objective	Outcome variable
To compare the fraction of subjects who achieve a complete inflammatory ocular inactivity by week 16	Complete abrogation of the ocular inflammatory signs, which is achieved within the first 16 weeks of the study, no treatment failure due to safety or intolerability; compliance with the initial (up to week 16) oral GCs tapering protocol, and completion of all study visits from Baseline to 16 weeks
To compare several Patient Reported Outcomes Measures (health- and vision-related quality of life, anxiety and depression) between treatment strategies	EuroQuol 5D-5L<br>Visual Functioning Questionnaire-25<br>Hospital Anxiety and Depression Scale
To compare the presence of the clinical components of the Good Clinical Response variable between treatment strategies during follow-up	Active chorioretinal lesions; active retinal vascular inflammation; macular edema; ACC; vitreous haze; and loss of CVA secondary to inflammation at baseline, week 16, week 52, and relapse visit
To compare the time to relapse after week 16 between treatment strategies	The time to inflammatory relapse between groups, defined as the time from visit 16 weeks until end of the study, loss of follow-up or appearance of at least one ocular inflammatory manifestation, in those individuals achieving a Good Clinical Response by visit 16 week
To compare the evolution of visual acuity during follow-up	Best corrected visual acuity (BCVA) during follow-up
To compare the development of anti-adalimumab antibodies during the follow-up, between those subjects treated with monotherapy and combination therapy	Assessment of anti-ADA antibodies (AAA) at week 15, 27, 51, and relapse.

For peer review only - http://bmjopen.bmj.com/site/about/guidelines.xhtml

To assess the cost-utility and cost-effectiveness from both a Health System and a Societal perspective of the combination therapy and the ADA monotherapy compared with MTX given alone	Direct and indirect cost, and Incremental Cost Effectiveness Ratios. Drug costs will be calculated individually for each patient taking as reference the price published in 2022 by the Health Ministry for the Spanish Health System. Outpatient and inpatient care, other medical cost, home care and productivity loss will be estimated based on the data from eSalud database[53] and from the Minimum Basic Data Set of the Spanish Health Ministry[54].

Security-related objective will establish which treatment strategy has better tolerability. Safety outcomes will be collected in the form of AEs, physical examination, and laboratory tests throughout the treatment period and up to 70 days after the last dose in this study. Gender of the subjects will be taken into account[55].

Pharmacogenetic and Proteomic-related objectives include identifying groups of subjects more likely to respond to the different treatment strategies, using genetic and proteomic biomarkers. For the former, subjects will be genotyped for known and validated genetics single nucleotide polymorphisms (SNPs) associated with a MTX[56] and ADA[57] response in different IMIDs. For the latter, a Discovery (shotgun proteomic analysis), Verification (Targeted proteomics using Multiple reaction monitoring test) and Validation (antibody-based microarrays absolute quantification tests) phases will be carried out, following optimized protocols and procedures[58–63].

Finally, a *Biobank substudy-related objective* will be carried out with the aim of boosting the translational research in the field of NIUs, by creating a collection of blood-derived samples (serum, plasma, total blood RNA, and DNA) from the participant subjects in order to advance in the identification and validation of biomarkers associated with treatment response or deepen in the pathophysiology of these conditions.

For peer review only

Settings and eligibility

The study population encompasses subjects diagnosed with non-infectious intermediate, posterior, or panuveitis with active disease within 180 days before the start of the study (Baseline visit), and either a documented failure to systemic or local GCs, or a chronic disease requiring GC-sparing ISD treatment. Main inclusion and exclusion criteria can be found at **Table 2**.

Table 2: Study participant inclusion and exclusion criteria

Inclusion criteria	Exclusion criteria
Adult subjects (≥18 years old);	Subjects with ocular histoplasmosis syndrome, or ocular masquerade syndromes;
Diagnosed with non-infectious intermediate, posterior, or panuveitis in at least one eye;	Evidence or history of malignancy;
Who have active disease within the previous 180 days before Baseline, and either	Corneal, lens or vitreous opacities precluding the visualization of the fundus;
A documented failure to systemic or local GCs in the previous 6 months, OR	Uncontrolled intraocular pressure;
A chronic disease necessitating GC-sparing immunosuppressive treatment (such as multifocal choroiditis with panuveitis, serpiginous choroidopathy, birdshot retinochoroidopathy, diffuse retinal vasculitis, Vogt-Koyanagi-Harada with bullous serous retinal and/or choroidal detachments, or sympathetic ophthalmia;	Best corrected visual acuity (CVA) <20/400;
Able and willing to self-administer subcutaneous (SC) injections or have a qualified person available to administer SC injections; and	History, symptoms and/or Magnetic Resonance Imaging (MRI) findings suggestive of a demyelinating disease;
A negative PPD test (or equivalent) and a chest x-ray (CXR) at Screening OR if positive PPD test (or equivalent) and/or a CXR consistent with prior tuberculosis (TB) exposure, the subject must initiate, be currently receiving or have documented completion of a course of TB prophylaxis therapy, according to clinical practice.	History of moderate to severe congestive heart failure (NYHA class III or IV)
	Behçet's disease or suspected of Behçet's disease;
	Previous exposure to TNFi therapies;

For peer review only - http://bmjopen.bmj.com/site/about/guidelines.xhtml

	Previous exposure to synthetic ISDs in the previous 6 months before Baseline;
	Prior intolerability, safety issues, or ineffectiveness of MTX and/or ADA;
	Use of GCs implants (Iluvien® within 3 years, Ozurdex® within 6 months before Baseline);
	Use of intraocular or periocular GCs injection within 90 days before Baseline;
	Ocular surgery within 30 days before Baseline;
	Planned (elective) eye surgery in the following 52 weeks from Baseline;
	Proliferative or severe non-proliferative diabetic retinopathy;
	Neovascular/wet age-related macular degeneration;
	Chronic structural damage considered by the Investigator to interfere with measurement of macular thickness, impede the potential for its normalization, or can cause damage independent of the inflammatory process;
	Systemic inflammatory disease considered by the Investigator as likely to require high GCs dosage or prohibited medications;
	Presence of chronic recurring infections (HBV, syphilis), active TB and/or a history of invasive infection;
	Positive pregnancy test;
	Breast-feeding or considering becoming pregnant during the study.

Subjects will be recruited from 16 Spanish academic hospitals located in different regions of Spain (Galicia, País Vasco, Castilla y León, Comunidad de Madrid, Castilla La Mancha, Comunidad Valenciana, and Comunidad Canaria). In addition, the Instituto de Investigación Biomédica de A Coruña (INIBIC) will carry out the proteomic analysis, and the Instituto de Investigación Sanitaria San Carlos (IdISSC) Musculoskeletal Pathology Group will be responsible for the pharmacogenetic analysis and development of prediction models for drug response.

Recruitment

It will extend over 18 months. The participating study sites attends several tens of new patients every year, with several hundreds or thousands being followed-up, and many of

For peer review only - http://bmjopen.bmj.com/site/about/guidelines.xhtml

these sites act as reference centres for other secondary and tertiary hospitals. Candidate subjects will be identified from patients lists AND/OR research databases AND among the new patients attended at the sites.

Subject will not receive any financial compensation for participating in the study.

To ensure subject's retention during follow-up, we will request contact details, carry out reminder phone calls/send emails, review barriers to attend appointments, and educate them in the significance of research follow-up even if they decide to discontinue the study drugs.

Intervention

All subjects entering the study will be centrally randomized at the Baseline visit into one of three study arms, in a 1:1:1 ratio:

Arm 1 will receive at the Baseline visit ADA 80 mg SC loading dose followed a week later by 40 mg every-other-week starting at Week 1. They will also receive MTX oral at the Baseline visit, with initial dose of 15mg/week, increasing up to 25 mg/week;

Arm 2: MTX with the same schedule as in Arm 1;

Arm 3: ADA with the same schedule as in Arm 1.

In addition to the study drugs, topical adjunctive eye medication will be allowed according to standard practice (intraocular pressure-lowering medication, cycloplegic agents, artificial tears, topical non-steroidal anti-inflammatory drugs…). The use of any biologic therapies with a potential therapeutic impact in NIUs, live vaccines, any other ISD besides the study drugs, intraocular GCs implants, or intraocular surgery will be prohibited for the duration of the trial.

To ensure a balance of patients across treatment groups and uveitis anatomic locations, patients will be stratified according to the main site of ocular inflammation (intermediate,

For peer review only - http://bmjopen.bmj.com/site/about/guidelines.xhtml

For peer review only

OR posterior/panuveitis). Randomization will not be stratified by site due to the small-expected number of subjects per site.

Block randomization will ensure that an equal number of patients are randomized to each study arm.

The allocation sequence will consist of a computer-generated random number list generated and held in the Clinical Research Unit of Hospital Clínico San Carlos (HCSC), hidden from participating Investigators. The allocation sequence will be computer-generated, and it will be implemented through the electronic case report form (eCRF; REDCap), which will assign the treatment group. Study data will be collected and managed using REDCap electronic data capture tools hosted at The Health Research Institute of the Hospital Clínico San Carlos[64,65] REDCap (Research Electronic Data Capture) is a secure, web-based software platform designed to support data capture for research studies, providing 1) an intuitive interface for validated data capture; 2) audit trails for tracking data manipulation and export procedures; 3) automated export procedures for seamless data downloads to common statistical packages; and 4) procedures for data integration and interoperability with external sources. After obtaining the informed consent for enrolment and confirming that all eligibility requirements have been met, the Unmasked Investigator (see next section) will log into the eCRF and perform the randomization. Unmasked investigators will then give their assigned treatment to the subjects.

Authorized Rescue Medication

During whole study up to two inflammatory relapses (unilateral or bilateral) will be allowed, one during the first period (baseline-week 16: ACC relapse) and 1 during the second period (week 16 -week 52: any location), before declaring the lack of response

For peer review only - http://bmjopen.bmj.com/site/about/guidelines.xhtml

to the assigned medication. Based on the location and severity of the relapse, a protocolized rescue treatment with Topical AND/OR Local OR Oral GCs will be allowed. In case inflammation cannot be supressed in 4 weeks, or a new relapse takes place after 4 weeks, it will be declared treatment failure and the subject will exit the study.

Masking

For the duration of the trial, both the study subject and the Unmasked Investigators will be aware of the treatment assigned. Considering the differences between study drugs (MTX, ADA) regarding appearance, route of administration and schedule, in order to make them unaware of the medication prescribed, the use of placebo would be necessary. However, taking into account that both drugs have been proven effective in the treatment of non-infectious uveitis, the duration of the trial (up to 52 weeks), and the need for bi-weekly subcutaneous injections of one of the drugs (ADA), we consider unethical for the subject the use of placebo in the present trial.

Besides the Unmasked Investigators, the rest of participating investigators will be considered Masked Investigators (ophthalmologists performing the clinical eye exams, visual acuity examiners, OCT operators, fundus photographers, fundus graders, and administrators of subject's questionnaires) and will not be aware of the treatment assigned to prevent bias in study outcomes.

Several steps will be taken to avoid the Masked Investigators discovering the subject assignment: they will have no part in handling or prescribing medication; subjects will be given dark bags to place and keep their medication in throughout the trial and study visits to minimize the chances of the Masked Investigators seeing the medications.

For peer review only

For peer review only - http://bmjopen.bmj.com/site/about/guidelines.xhtml

Additionally, subjects will meet with the Unmasked Investigator first, before seeing any Masked Investigators, keeping any study medication in his/her office for the entire patient visit; reviewing the appearance of any AE with the subjects before seeing any Masked Investigators, and reminding the subjects not to discuss their dosing and mediation name with the Masked Investigators.

Study Procedures

Study visits will be the Baseline visit, visits at Week 1, 4 and every 4 weeks thereafter until a) the subject is determined as unable to achieve complete resolution of the ocular inflammatory process by week 16, OR; b) the subject is determined as unable to maintain a complete resolution of the ocular inflammatory process, between weeks 16 and 52 OR; c) the subject completes 52 weeks of this clinical trial, OR; d) the study is stopped due to the findings of the Data Security Monitoring Board, OR; e) the subject meets any of the study finalization criteria.

Informed consent has to be acquired before carrying out any study procedure, including screening tests (**supplementary file 1** contains a sample informed consent). Before Baseline (when the subject is randomized), several screening visits can take place up to 14 days before that visit, in order to obtain all the required complementary tests to assess eligibility. The visit window for all scheduled visits is ±3 days through Week 4 and ±7 days for all visits following the Week 4 study visit. **Table 3** provides an overview of the study activities.

After the trial, all patients will return to standard care and will be able to continue with their assigned study medication.

Table 3: Study activities.

For peer review only - http://bmjopen.bmj.com/site/about/guidelines.xhtml

Activity	Screening	Baseline	w1	w4	w8	w12	w16	w20	w24	w28	w32	w36	w40	w44	w48	FET	Relapse	Unsch.	70-day Follow-up
Informed Consent	X																		
Inclusion/Exclusion Criteria	X	X																	
Medical/Surgical History	X	X																	
History of tobacco and alcohol use	X	X																	
Uveitis History	X	X																	
Vital Signs/ Weight/Height	X	X														X	(X)		
Physical Exam	X	X														X	(X)		
Symptom Directed Physical Exam			X	X	X	X	X	X	X	X	X	X	X	X	X		X		
Chest X-Ray	X															X[g]			
Cerebral MRI[a]	(X)																		
TB Screening	X																		
Serum Pregnancy Test[f]	X	X	X	X	X	X	X	X	X	X	X	X	X	X	X	X			
Hepatitis B/C Screening	X																		
Syphilis Testing (FTA)	X																		
Randomization		X																	
Best Corrected Visual Acuity Testing	(X)[b]	(X)[b]		X	X	X	X	X	X	X	X	X	X	X	X	X	X		
Tonometry	(X)[b]	(X)[b]		X	X	X	X	X	X	X	X	X	X	X	X	X	X		
Slit Lamp Exam	(X)[b]	(X)[b]		X	X	X	X	X	X	X	X	X	X	X	X	X	X		
Dilated Indirect Ophthalmoscopy	(X)[b]	(X)[b]		X	X	X	X	X	X	X	X	X	X	X	X	X	X		
Fundus Photography	(X)[b]	(X)[b]					X									X	X		
Optical Coherence Tomography	(X)[b]	(X)[b]		X	X	X	X	X	X	X	X	X	X	X	X	X	X		
EuroQol-5D	(X)[b]	(X)[b]		X	X	X	X	X	X	X	X	X	X	X	X	X	X		
Visual Functioning Questionnaire-25	(X)[b]	(X)[b]					X									X	X		
Hospital Anxiety and Depression Scale	(X)[b]	(X)[b]					X									X	X		
Hematology/Chemistry	X	(X)[c]	X	X	X	X	X		X		X		X		X	X	(X)		
Monitor Adverse Events		(X)	X	X	X	X	X	X	X	X	X	X	X	X	X	X	X	X	X

Monitor Concomitant Medication	X	X	X	X	X	X	X	X	X	X	X	X	X	X	X	X	X	X	X
Review of Patient Diary			X	X	X	X	X	X	X	X	X	X	X	X	X	X	(X)		
Perform Drug Accountability			X	X	X	X	X	X	X	X	X	X	X	X	X	X	(X)		
Assessment of direct and indirect costs			X	X	X	X	X	X	X	X	X	X	X	X	X	X	(X)		
Dispense Study Drugs		X	X	X	X	X	X	X	X	X	X	X	X	X	X		(X)		
Proteomic blood sample	(X)[d]	(X)[d]					X									X	X		
Pharmacogenetic blood sample	(X)[d]	(X)[d]																	
Adalimumab levels and Anti-Adalimumab Antibodies	(X)[d]	(X)[d]					X[e]			X[e]						X[e]	X[e]		

FET: Final (w52) / Early termination visit. Unsch: Unscheduled visit.

[a]Cerebral MRI will be performed only in those subjects with intermediate uveitis or signs of intermediate uveitis.

[b]Values from the Baseline visit or from a screening visit up to 14 days before the Baseline Visit will be used as the values from the Baseline visit.

[c]Total Blood count, renal and liver function test from up to 4 weeks before the Baseline visit will the required before entering the study.

[d]Blood sample will be drawn after signature of the informed consent, as soon as possible, ideally before start of oral GCs.

[e]Blood samples for the Anti-Adalimumab Antibodies will be drawn the week before visits 16, 28, 52, Early termination and relapse visits, up to 24h before the patient is scheduled to receive the ADA dosage, and always before the medication is taken.

[F] Only in women in childbearing

[g]Chest X ray will be performed at the 52 weeks/Early termination visit in case the patient had a positive TB test at abaseline.

For peer review only - http://bmjopen.bmj.com/site/about/guidelines.xhtml

Data Management and Monitoring

An Unmasked Investigator in each centre will review and crosscheck for consistency and completeness all data collected in RedCap within 24h of the study visit.If the forms are not filled out completely, the responsible person will be contacted for providing the missing data.. An external monitoring service will conduct regular checks of the data regarding errors and inconsistencies, supervising data collection, management and quality control, and will submit queries to the site investigators.

Safety

Non-serious AEs will be defined as an unfavorable and unintended sign (including abnormal laboratory findings), symptom or disease temporally associated with the use of the study medication or procedure, whether or not considered related to the study medication. Both MTX and ADA may be temporarily suspended in case of non-serious AEs, such as laboratory alterations, infections, and intolerance. In addition, MTX dosage may also be reduced in case of laboratory alterations and/or intolerance. Dose reductions and temporary discontinuations will be decided by the Masked Investigator, who must remain unaware of the medication and dosage assigned to the subject, so preconceptions regarding the study drugs do not interfere with their management. The Masked Investigator will issue three recommendations; one for each medication arm the subject may be included. Then, the Unmasked Investigator will implement the recommendation according to the arm the subject is included.

SAEs will be defined as any adverse event that results in death, is life threatening, requires hospitalization, or prolongation of existing hospitalization, results in persistent or significant disability or incapacity, or is a congenital anomaly or birth defect.

For peer review only - http://bmjopen.bmj.com/site/about/guidelines.xhtml

All AEs will be recorded in clinical records and a medically qualified investigator will assess the relationship of SAEs to the study medications.

All SAE that occurs during the trial must be reported immediately by mail or fax to the Pharmacovigilance Unit within 24 hours of its occurrence. The investigator will complete and sign the SAE notification form to be sent by e-mail.

The Pharmacovigilance Unit will review the form received and, if applicable, ask for additional information to the investigator. The investigator should provide the requested information or any new information regarding the case, especially if the initial assessment in severity or causality has been changed, following the procedure previously described.

The Pharmacovigilance Unit is responsible for submitting as soon as possible all Suspected Unexpected Serious Adverse Reactions (SUSARs) collected during the study to the Spanish Health Authorities and Ethic Committee, according with the Spanish legislation: no later than 15 calendar days (7 in case of fatal or life-threatening cases) after first knowledge by the sponsor that the case meets the minimum criteria for expedited reporting.

Data and Safety Monitoring Committee

Only after the Data and Safety Monitoring Committee (DSMC) reviews and approves the protocol will patients be enrolled. In addition, the group will meet regularly throughout the study and review information on data quality, enrolment, patient retention, and study outcomes according to DSMC charter. The DSMC will be independent from the Sponsor, Funding Body and Principal Investigator, and will include experts in the fields of ophthalmology, rheumatology and epidemiology.

Adherence

For peer review only - http://bmjopen.bmj.com/site/about/guidelines.xhtml

Adherence will be monitored using a Patient's Diary (where they will register all study medication administered outside of the study visit (i.e., at home), including reasons for missing dosages) and by verifying the returned empty medication (partially/completely empty blisters of study medication, AND/OR cartons and sharps containers for MTX, ADA and oral GCs).

Patient and Public Involvement

No patient involved.

Statistical analysis plan

The Intention to treat (ITT) set will include all subjects who were randomized.

The safety set will consist of all subjects who received at least one dose of study medications. Per protocol analysis will also carry out. Missing data will be imputed using multiple imputation-chained equations

Sample Size Determination

ADA arm: after one year (50 weeks) of treatment, 20.3% of NIU patients achieved an outcome similar to our *primary efficacy outcome*[66];

MTX arm: after 6 months of treatment, 65% of NIU patients achieve also similar outcome[67]; we estimate that of those patients, 18%[68] and 16%[69] will be unable to maintain our outcome due to AEs and inefficacy, respectively; therefore, at 1 year, 43% will achieve our *primary efficacy outcome*. This figure will be assumed as the percentage of subjects treated with monotherapy achieving the primary efficacy outcome.

For peer review only - http://bmjopen.bmj.com/site/about/guidelines.xhtml

MTX+ADA arm: we will assume that combination therapy will increase the percentage of subjects achieving our *primary efficacy outcome* by 23% compared with the monotherapy arms.

To detect statistically significant differences between groups with a power of 80% and a significance level of 0.05, it will be necessary to recruit 54 patients per study arm (162 in total). Since the follow-up period is 52 weeks, losses of 15% will be assumed, increasing the sample size to 64 patients per study arm (192 patients in total). In order to test the difference between the treatments, superiority or relevant clinical improvement has been considered from a delta of 5% of the effect.

Planned Methods of Statistical Analysis for efficacy-related objectives

The primary analysis will be a Mantel–Haenszel test (MHT), stratified by NIU location, comparing the combination therapy arm and the single immunosuppressive drugs arms (both arms combined), and performed in the ITT set. Achievement of the Good Clinical response will be considered the binary response variable: those subjects achieving a Good Clinical vs. those not achieving the outcome (regardless the cause). The exposure variables will be the treatment arm: those subjects receiving combination therapy vs. those receiving either monotherapy. Uveitis location will be the strata: intermediate uveitis vs. posterior OR panuveitis. If the results of the primary analysis are significant, then pairwise comparisons using MHT stratified by NIU location will be carried out. P-values of the pairwise comparisons will be adjusted using the Bonferroni method. No interim efficacy-related analysis will be carried out.

Secondary efficacy-related analyses are designed to test the hypothesis that treatment assignment affects a given outcome, after controlling for selected covariates. Details can be found at **Table 4**.

For peer review only - http://bmjopen.bmj.com/site/about/guidelines.xhtml

Table 4: Planned Methods of Statistical Analysis for efficacy-related secondary outcomes.

Outcome variable	Statistical Analysis
Complete abrogation of the ocular inflammatory signs, which is achieved within the first 16 weeks of the study, no treatment failure due to safety or intolerability; compliance with the initial (up to week 16) oral GCs tapering protocol, and completion of all study visits from Baseline to 16 weeks	MHT, stratified by NIU location. If p-value < 0.05, pairwise comparisons using MHT stratified by NIU location will be carried out with Bonferroni adjustment of the pairwise p-values
EQ5D	GEE models nested by patient[70,71] and adjusted by study visit (continuous) and treatment arm (discrete) will be carried out, using a Gaussian family and Identity as link function. Different covariable structures will be tested (independent and exchangeable) and compared using the Bayesian Information Criteria. Time x Study arm interactions will assess different effects of time in the evolution of the outcome by arm. p-value <0.05 will be considered as a significant interaction
Visual Functioning Questionnaire-25<br>Hospital Anxiety and Depression Scale	Changes between Baseline and w16, and the FET visit will be compared between treatment groups using ANOVA
Active chorioretinal lesions; active retinal vascular lesions; macular edema; ACC; vitreous haze; and loss of CVA secondary to inflammation at baseline, week 16, week 52, and relapse visit	MHT, stratified by NIU location. If p-value < 0.05, pairwise comparisons using MHT stratified by NIU location will be carried out with Bonferroni adjustment of the pairwise p-values
The time to inflammatory relapse between groups, defined as the time from visit 16 weeks until end of the study, loss of follow-up or appearance of at least one ocular inflammatory manifestation, in those individuals achieving a Good Clinical Response by visit 16 week	Time to relapse between arms will be analyzed using log-rank test at a two-sided significance level of 5%. Dropouts due to reasons other than inability to maintain a Good Clinical Response will be considered as censored observations at the time of dropping out. Only subjects able to achieve a Good Clinical Response by visit week 16 will be analyzed. We will consider both the time until the onset of the first inflammatory manifestation, each inflammatory manifestation, the first inflammatory manifestation that does not resolve in the following 4 weeks, and each inflammatory manifestation that does not resolve in the following 4 weeks.
Best corrected visual acuity during follow-up	GEE models

For peer review only - http://bmjopen.bmj.com/site/about/guidelines.xhtml

Assessment of anti-ADA antibodies (AAA) at week 15, 27, 51, and relapse.	MHT, stratified by NIU location. If p-value < 0.05, pairwise comparisons using MHT stratified by NIU location will be carried out with Bonferroni adjustment of the pairwise p-values
Direct and indirect cost, and Incremental Cost Effectiveness Ratios	Cost utility and cost-effectiveness analysis from the perspectives of the National Health System and the Society will be performed. EQ5D scores will derive utility values representing health related quality of life. QALYs will be calculated by the area under the curve assuming a linear evolution of EQ5D values between visits. The average number of QALYs per patient will be calculated for each study arms. Effectiveness will be defined using our primary efficacy outcome. An average cost per patient will be calculated for each study arm, including direct (drug cost, outpatient and inpatient care, and other medical cost), and indirect cost (home care, productivity loss): Incremental cost-effectiveness ratios will be calculated, using the MTX monotherapy arm as comparison

GCs: Glucocorticoids; MTX: Methotrexate; ADA: Adalimumab; QALYs: Quality-adjusted life years; EQ5D: EuroQuol 5D-5L; GEE: Generalized estimating equations; MTH: Mantel-Haetzel; NIU: Non-infectious uveitis

Planned Methods of Statistical Analysis for Safety-related aims

The safety analysis will be performed in the safety set. Treatment-emergent AEs (events with an onset date on or after the first study drug administration until 70 days following the last study drug administration) will be summarized by treatment group using descriptive statistics. SAEs with onset after informed consent but before the first study drug administration will be considered as pretreatment SAEs and reported separately. AEs will be tabulated by system organ class and preferred term whereby the most current implemented MedDRA dictionary will be used. In addition, summaries by severity and relationship to study drug will be done. Certain AEs, such as serious or severe, leading to premature withdrawal, will be listed and described in detail. AEs of special interest for treatments will be defined in the Statistical analysis plan (SAP) and

For peer review only - http://bmjopen.bmj.com/site/about/guidelines.xhtml

analysed separately. In addition to the descriptive statistics provided, Fisher's exact test will be used for comparisons between treatment groups.

Genetic Analysis

SNPs genotypes will be determined by real-time PCR amplification using Taqman probes and following standard procedures. Duplicate genotypes of 10% of the samples, concordance (all $p > 0.05$) with the Hardy–Weinberg equilibrium and with SNP frequencies in the HapMap European collection will be used for quality control. Comparison of the proportion of subjects achieving the primary efficacy outcome between genotypes will be carried out using a χ^2 test or Fisher's exact test, when required. Dominant, recessive, and additive models of effects will be considered for each SNP. P-values will be adjusted using the Bonferroni method. Each study arm will be analysed separately.

Proteomic Analyses

Discovery Phase: Shotgun proteomic analysis[58–60] will be performed on serum samples from a representative group of subjects from each study arm with extreme responses (n=20): those achieving the *primary efficacy outcome* and those not being able to achieve a good clinical response by Week 16.

Verification Phase: Targeted proteomics will be used for verification of protein markers with predictive potential in a randomly selected larger samples set (n=80). After multiple reaction monitoring (MRM) tests, relative quantification methods of the proteins will be designed[61].

For peer review only

Validation Phase: Best candidates from the previous phase will be validated using absolute quantification tests (antibody-based microarrays) in the whole set of subjects[62,63].

Patient subgroup identification

Based on Baseline visits patient's characteristics (demographic, disease, and clinical-related variables), those genotypes significantly associated with the primary efficacy outcome, and the previously identified serum proteins in the verification phase, prediction models for MTX, ADA, and Combination therapy response will be developed using a machine learning method (Random Forests[72]). Models' performance will be assessed with the area under the receiver operating characteristic curve, and calibration curves. Models using only clinical data, only biomarkers data, and the combination of both will be developed, to assess the contribution of biomarkers to the models' predictive ability. Due to the modest sample size we plan to recruit, we will not divide our sample in training, validation and test datasets. All subjects will be considered part of the training dataset, and a ten-fold cross-validation will be carried out to internally validate our models.

ETHICS AND DISSEMINATION

The protocol (version 2, 11/09/2020), annexes and informed consent forms have been approved by the Clinical Research Ethic Committee (CREC) at the Hospital Clínico San Carlos (Madrid, Spain) and the Spanish Agency for Medicines and Health Products (AEMPS). The local approvals corresponding to the participating centres will be obtained and documented before starting the study in that centre as per centre

For peer review only - http://bmjopen.bmj.com/site/about/guidelines.xhtml

requirements. The promoter will be the CREC interlocutor corresponding to his/her centre in everything related to the present study. It will keep CREC informed of the evolution of the study in the centre and of the possible minor incidents and modifications that may occur. Any relevant modification to the protocol must receive express approval from the reference CREC and the AEMPS before its implementation, unless there are risk circumstances for the participating subjects, in which case the precise measures to ensure the integrity of the study subjects will be implemented immediately, pending the corresponding approvals. The trial is registered at clinicaltrialsregister.eu (EudraCT:2020-000130-18) and clinicaltrials.gov (NCT04798755).

No study related activities will be carried out before obtaining a written informed consent from the patient. The investigator will be responsible for: a) providing each patient with an information sheet about the trial and the objectives, methods, foreseeable benefits and potential risks of the study, b) discussing the information with the patient, in terms understandable for the subject, and c) explaining to patients that they are totally free to refuse their participation in the study or to abandon it at any time and for any reason. If the subject agrees to participate in the Biobank Substudy, a second independent informed consent will be collected, which will include the possibility of storing the samples not used in the present study in the Collection of Samples for Research in Rheumatic Diseases of the Rheumatology Department of the Hospital Clinico San Carlos (and, in a second phase, when the Coordinating Investigator of this study deems it appropriate and always in the event that the samples has not been used up, the remainder will be stored in the Hospital Clinico San Carlos Biobank).

All the data will be treated confidentially at any times: data will be seudonimized, the paper forms will be kept in locked cabinets, the eCRFis located in a secure server, and

For peer review only - http://bmjopen.bmj.com/site/about/guidelines.xhtml

the person in charge of the analysis will not be able to access identification data of the subjects.

Table 5 summarises the study protocol and trial registration information.

Table 5: Trial registration data and protocol summary

Data category	Information
Primary registry and trial identifying number	EudraCT: 2020-000130-18
Date of registration in primary registry	09/03/2021
Secondary identifying numbers	ClinicalTrials.gov: NCT04798755
Source of monetary or material support	Instituto de Salud Carlos III
Primary sponsor	Fundación para la Investigación Biomédica del Hospital Clínico San Carlos
Contact for queries	Luis Rodriguez-Rodriguez, MD (lrrodriguez@salud.madrid.org)
Public Title	Combination THerapy with mEthotrexate and adalImumAb for uveitis (CoTHEIA)
Scientific title	Efficacy , safety and cost-effectiveness of methotrexate, adalimumab, or their combination in non-infectious non-anterior uveitis: a multicenter, randomized, parallel 3 arms, active-controlled, phase 3 open label with blinded outcome assessment study
Countries of recruitment	Spain
Health condition or problem studied	Non-infectious non anterior uveitis
Intervention(s)	Intervention 1: Adalimumab 40 mg every-other-week (plus a 80 mg SC loading dose) + Methotrexate oral, up to 25 mg/week, both for a duration of 52 weeks.
	Intervention 2: Methotrexate with the same schedule as in Intervention 1
	Intervention 3: Adalimumab with the same schedule as in Intervention 1
Key inclusion and exclusion criteria	Age ≥18 years old<br>Diagnosed with non-infectious intermediate, posterior, or panuveitis in at least one eye;<br>Active ocular disease

For peer review only - http://bmjopen.bmj.com/site/about/guidelines.xhtml

	Lack of satisfactory response to systemic and/or local glucocorticoid (GC) therapy AND/OR diagnoses of a chronic disease usually necessitating GC-sparing immunosuppressive treatment.
Study type	Phase III, multicenter, prospective, randomized, single-blinded with masked outcome assessment, parallel 3 arms with 1:1:1 allocation, active-controlled, superiority study design
Date of first enrolment	N/A
Target sample size	64 per treatment arm (192 in total)
Recruitment status	Not yet recruiting
Primary outcome	Complete resolution of the ocular inflammatory signs, which is achieved within the first 16 weeks of the study, and maintained during follow-up until the end of the study (week 52);
Key secondary outcomes	Safety, cost-effectiveness

Data obtained through this study may be provided to qualified researchers with academic interest in uveitis. Data or samples shared will be coded, and donated to a Registered Biobank and made available under legal requirement. Approval of the request and execution of all applicable agreements are prerequisites to the sharing of data with the requesting party.

Regarding dissemination, in order to communicate the clinical trial progress and findings to a broad group of stakeholders, we will elaborate a Dissemination plan which will include production of materials adapted to scientific meetings, scientific publications, patients, and other stakeholders. A summary of the final version of the study protocol will be made available through the Spanish Clinical Trial Registry and Clinicaltrials.gov database. The promoter will be the only with access to the participant-level data, following the regulation on data protection.

CONTRIBUTORSHIP STATEMENT

Luis Rodriguez-Rodriguez (LRR), Amanda López Picado (ALP), Esperanza Pato Cour (EPC) and David Diaz Valle (DDV) conceived of the study.

For peer review only - http://bmjopen.bmj.com/site/about/guidelines.xhtml

LRR, ALP, EPC, DDV, Ester Carreño (EC), Miguel Cordero-Coma (MCC), Alex Fonollosa (AF), Angel Garcia Aparicio (AGA), Javier Garcia Gonzalez (JGG), Santiago Muñoz (SM), Ana Rivas (AR), and Luis Tebar (LT) initiated the study design and helped with implementation.

LRR is the funding holder.

ALP and AR provided statistical expertise in clinical trial design.

Valentina Calamia (VC) provided expertise regarding the proteomic analysis design.

LRR provided expertise regarding the pharmacogenetic analysis.

ALP, AR and LRR will conduct the primary statistical analysis.

AR, ALP, VC, EC, Lidia Cocho (LC), MCC, AF, Felix M Francisco Hernandez (FMFH), AGA, JGG, Jose Juan Mondejar (JJM), Leticia Lojo Oliveira (LLO), Llucí Martinez-Costa (LMC), SMM, Diana Peiteado (DP), Jose Antonio Pinto (JAP), Beatriz Rodriguez Lozano (BRL), EPC, DDV, Elena Molina (EM), LT and LRR contributed to refinement of the study protocol and approved the final manuscript.

COMPETING INTERESTS

None declared

FUNDING

This work was supported by the Instituto de Salud Carlos III, grant number [ICI19/00020].

For peer review only - http://bmjopen.bmj.com/site/about/guidelines.xhtml

REFERENCES

1 Caspi RR. A look at autoimmunity and inflammation in the eye. *J Clin Invest* 2010;**120**:3073–83. doi:10.1172/JCI42440

2 WHO. The World Health Report, Global data on visual impairments. 2010.http://www.who.int/blindness/GLOBALDATAFINALforweb.pdf

3 Suttorp-Schulten MS, Rothova A. The possible impact of uveitis in blindness: a literature survey. *Br J Ophthalmol* 1996;**80**:844–8. doi:10.1136/bjo.80.9.844

4 Rothova A, Suttorp-van Schulten MS, Frits Treffers W, *et al.* Causes and frequency of blindness in patients with intraocular inflammatory disease. *Br J Ophthalmol* 1996;**80**:332–6.http://www.pubmedcentral.nih.gov/articlerender.fcgi?artid=505460&tool=pmcentrez&rendertype=abstract (accessed 25 Jul 2014).

5 de Smet MD, Taylor SRJ, Bodaghi B, *et al.* Understanding uveitis: The impact of research on visual outcomes. *Prog Retin Eye Res* 2011;**30**:452–70. doi:10.1016/j.preteyeres.2011.06.005

6 Williams GJ, Brannan S, Forrester J V, *et al.* The prevalence of sight-threatening uveitis in Scotland. *Br J Ophthalmol* 2007;**91**:33–6. doi:10.1136/bjo.2006.101386

7 Gritz DC, Wong IG. Incidence and prevalence of uveitis in Northern California: The Northern California Epidemiology of Uveitis Study. Ophthalmology. 2004;**111**:491–500. doi:10.1016/j.ophtha.2003.06.014

8 Chang JH-M, Wakefield D. Uveitis: a global perspective. *Ocul Immunol Inflamm* 2002;**10**:263–79. doi:10.1076/ocii.10.4.263.15592

9 Arriola-Villalobos P, Abásolo L, García-Feijoo J, *et al.* Vision-related Quality of Life in Patients with Non-infectious Uveitis: A Cross-sectional Study. *Ocul*

For peer review only - http://bmjopen.bmj.com/site/about/guidelines.xhtml

Immunol Inflamm 2017;:1–9. doi:10.1080/09273948.2017.1285034

10 Miserocchi E, Modorati G, Mosconi P, *et al.* Quality of life in patients with uveitis on chronic systemic immunosuppressive treatment. *Ocul Immunol Inflamm* 2010;**18**:297–304. doi:10.3109/09273941003637510

11 DARRELL RW, WAGENER HP, KURLAND LT. Epidemiology of uveitis. Incidence and prevalence in a small urban community. *Arch Ophthalmol (Chicago, Ill 1960)* 1962;**68**:502–14.http://www.ncbi.nlm.nih.gov/pubmed/13883604

12 Suhler EB, Lloyd MJ, Choi D, *et al.* Incidence and prevalence of uveitis in Veterans Affairs Medical Centers of the Pacific Northwest. *Am J Ophthalmol* 2008;**146**:890-6.e8. doi:10.1016/j.ajo.2008.09.014

13 E. Kirbach, S., A. Hayes, O., A. Cifaldi M. The Economic Burden of Uveitis. *Arthritis Rheum* 2010;**62**:788. doi:10.1002/art.28556

14 Adán-Civera AM, Benítez-Del-Castillo JM, Blanco-Alonso R, *et al.* Burden and direct costs of non infectious uveitis in Spain. *Reumatol Clin* 2015;**12**:196–200. doi:10.1016/j.reuma.2015.08.004

15 Durrani OM, Meads CA, Murray PI. Uveitis: A potentially blinding disease. Ophthalmologica. 2004;**218**:223–36. doi:10.1159/000078612

16 Jalil A, Yin K, Coyle L, *et al.* Vision-related quality of life and employment status in patients with uveitis of working age: a prospective study. *Ocul Immunol Inflamm* 2012;**20**:262–5. doi:10.3109/09273948.2012.684420

17 Dick AD, Rosenbaum JT, Al-Dhibi HA, *et al.* Guidance on Noncorticosteroid Systemic Immunomodulatory Therapy in Noninfectious Uveitis. *Ophthalmology* 2018;**125**:757–73. doi:10.1016/j.ophtha.2017.11.017

18 Smith JR, Rosenbaum JT. Management of uveitis: a rheumatologic perspective.

For peer review only - http://bmjopen.bmj.com/site/about/guidelines.xhtml

Arthritis Rheum 2002;**46**:309–18. doi:10.1002/art.503

19 Jabs D a, Rosenbaum JT, Foster CS, *et al.* Guidelines for the use of immunosuppressive drugs in patients with ocular inflammatory disorders: recommendations of an expert panel. *Am J Ophthalmol* 2000;**130**:492–513. doi:10.1016/S0002-9394(00)00659-0

20 Becker MD, Smith JR, Max R, *et al.* Management of sight-threatening uveitis: new therapeutic options. *Drugs* 2005;**65**:497–519. doi:10.2165/00003495-200565040-00005

21 Jabs DA. Immunosuppression for the Uveitides. *Ophthalmology* 2018;**125**:193–202. doi:10.1016/j.ophtha.2017.08.007

22 Rathinam SR, Babu M, Thundikandy R, *et al.* A randomized clinical trial comparing methotrexate and mycophenolate mofetil for noninfectious uveitis. *Ophthalmology* 2014;**121**:1863–70. doi:10.1016/j.ophtha.2014.04.023

23 Rathinam SR, Gonzales JA, Thundikandy R, *et al.* Effect of Corticosteroid-Sparing Treatment With Mycophenolate Mofetil vs Methotrexate on Inflammation in Patients With Uveitis: A Randomized Clinical Trial. *JAMA* 2019;**322**:936–45. doi:10.1001/jama.2019.12618

24 Gangaputra S, Newcomb CW, Liesegang TL, *et al.* Methotrexate for Ocular Inflammatory Diseases. *Ophthalmology* 2009;**116**:2188–99. doi:10.1016/j.ophtha.2009.04.020

25 Salliot C, van der Heijde D. Long-term safety of methotrexate monotherapy in patients with rheumatoid arthritis: a systematic literature research. *Ann Rheum Dis* 2009;**68**:1100–4. doi:10.1136/ard.2008.093690

26 Baker KB, Spurrier NJ, Watkins AS, *et al.* Retention time for corticosteroid-sparing systemic immunosuppressive agents in patients with inflammatory eye

For peer review only

For peer review only - http://bmjopen.bmj.com/site/about/guidelines.xhtml

disease. *Br J Ophthalmol* 2006;**90**:1481–5. doi:10.1136/bjo.2006.097998

27 Abásolo L, Rosales Z, Díaz-Valle D, *et al.* Immunosuppressive Drug Discontinuation in Noninfectious Uveitis From Real-Life Clinical Practice: A Survival Analysis. *Am J Ophthalmol* 2016;**169**:1–8. doi:10.1016/j.ajo.2016.06.007

28 Jaffe GJ, Dick AD, Brézin AP, *et al.* Adalimumab in Patients with Active Noninfectious Uveitis. *N Engl J Med* 2016;**375**:932–43. doi:10.1056/NEJMoa1509852

29 Nguyen QD, Merrill PT, Jaffe GJ, *et al.* Adalimumab for prevention of uveitic flare in patients with inactive non-infectious uveitis controlled by corticosteroids (VISUAL II): a multicentre, double-masked, randomised, placebo-controlled phase 3 trial. *Lancet (London, England)* 2016;**388**:1183–92. doi:10.1016/S0140-6736(16)31339-3

30 Arida A, Fragiadaki K, Giavri E, *et al.* Anti-TNF agents for Behçet's disease: analysis of published data on 369 patients. *Semin Arthritis Rheum* 2011;**41**:61–70. doi:10.1016/j.semarthrit.2010.09.002

31 Leinonen ST, Aalto K, Kotaniemi KM, *et al.* Anti-adalimumab antibodies in juvenile idiopathic arthritis-related uveitis. *Clin Exp Rheumatol*;**35**:1043–6.http://www.ncbi.nlm.nih.gov/pubmed/29148422

32 Llorenç V, Cordero-Coma M, Blanco-Esteban A, *et al.* Drug Retention Rate and Causes of Discontinuation of Adalimumab in Uveitis: Real-World Data from the Biotherapies in Uveitis (BioÚvea) Study Group. *Ophthalmology* 2020;**127**:814–25. doi:10.1016/j.ophtha.2019.11.024

33 Michaud TL, Rho YH, Shamliyan T, *et al.* The comparative safety of tumor necrosis factor inhibitors in rheumatoid arthritis: a meta-analysis update of 44

For peer review only - http://bmjopen.bmj.com/site/about/guidelines.xhtml

trials. *Am J Med* 2014;**127**:1208–32. doi:10.1016/j.amjmed.2014.06.012

34 Bongartz T, Sutton AJ, Sweeting MJ, *et al.* Anti-TNF antibody therapy in rheumatoid arthritis and the risk of serious infections and malignancies: systematic review and meta-analysis of rare harmful effects in randomized controlled trials. *JAMA* 2006;**295**:2275–85. doi:10.1001/jama.295.19.2275

35 Joshi L, Talat L, Yaganti S, *et al.* Outcomes of changing immunosuppressive therapy after treatment failure in patients with noninfectious uveitis. *Ophthalmology* 2014;**121**:1119–24. doi:10.1016/j.ophtha.2013.11.032

36 Menezo V, Lau C, Comer M, *et al.* Clinical outcome of chronic immunosuppression in patients with non-infectious uveitis. *Clin Experiment Ophthalmol* 2005;**33**:16–21. doi:10.1111/j.1442-9071.2005.00904.x

37 Goldberg NR, Lyu T, Moshier E, *et al.* Success with single-agent immunosuppression for multifocal choroidopathies. *Am J Ophthalmol* 2014;**158**:1310–7. doi:10.1016/j.ajo.2014.08.039

38 Cervantes-Castañeda RA, Gonzalez-Gonzalez LA, Cordero-Coma M, *et al.* Combined therapy of cyclosporine A and mycophenolate mofetil for the treatment of birdshot retinochoroidopathy: a 12-month follow-up. *Br J Ophthalmol* 2013;**97**:637–43. doi:10.1136/bjophthalmol-2012-302123

39 Akpek EK, Baltatzis S, Yang J, *et al.* Long-term immunosuppressive treatment of serpiginous choroiditis. *Ocul Immunol Inflamm* 2001;**9**:153–67.http://www.ncbi.nlm.nih.gov/pubmed/11815884

40 Hooper PL, Kaplan HJ. Triple agent immunosuppression in serpiginous choroiditis. *Ophthalmology* 1991;**98**:944–51; discussion 951-2.http://www.ncbi.nlm.nih.gov/pubmed/1866149

41 Pascalis L, Pia G, Aresu G, *et al.* Combined cyclosporin A-steroid-MTX

For peer review only - http://bmjopen.bmj.com/site/about/guidelines.xhtml

treatment in endogenous non-infectious uveitis. *J Autoimmun* 1993;**6**:467–80. doi:10.1006/jaut.1993.1039

42 Agarwal M, Ganesh SK, Biswas J. Triple agent immunosuppressive therapy in Vogt-Koyanagi-Harada syndrome. *Ocul Immunol Inflamm* 2006;**14**:333–9. doi:10.1080/09273940600976938

43 Arcinue CA, Radwan A, Lebanan MO, *et al.* Comparison of two different combination immunosuppressive therapies in the treatment of Vogt-Koyonagi-Harada syndrome. *Ocul Immunol Inflamm* 2013;**21**:47–52. doi:10.3109/09273948.2012.728668

44 Davatchi F, Sadeghi Abdollahi B, Shams H, *et al.* Combination of pulse cyclophosphamide and azathioprine in ocular manifestations of Behcet's disease: longitudinal study of up to 10 years. *Int J Rheum Dis* 2014;**17**:444–52. doi:10.1111/1756-185X.12248

45 Katsuyama A, Kusuhara S, Nishisho R, *et al.* Long-term efficacy and safety of infliximab and cyclosporine combination therapy for refractory uveoretinitis in Behçet's disease. *Clin Ophthalmol* 2019;**13**:521–7. doi:10.2147/OPTH.S198648

46 Kotaniemi K. Late onset uveitis in juvenile-type chronic polyarthritis controlled with prednisolone, cyclosporin A and methotrexate. *Clin Exp Rheumatol*;**16**:469–71.http://www.ncbi.nlm.nih.gov/pubmed/9706430

47 Ganesh SK, Narayana KM, Biswas J. Peripapillary choroidal atrophy in sympathetic ophthalmia and management with triple-agent immunosuppression. *Ocul Immunol Inflamm* 2003;**11**:61–5.http://www.ncbi.nlm.nih.gov/pubmed/12854028

48 Cimino L, Finzi G, Mora P, *et al.* Effect of a drug combination treatment on ocular perfusion in recurrent idiopathic intermediate uveitis. *Ocul Immunol*

For peer review only - http://bmjopen.bmj.com/site/about/guidelines.xhtml

Inflamm 2003;**11**:299–303.http://www.ncbi.nlm.nih.gov/pubmed/14704901

49 Hazlewood GS, Barnabe C, Tomlinson G, *et al.* Methotrexate monotherapy and methotrexate combination therapy with traditional and biologic disease modifying antirheumatic drugs for rheumatoid arthritis: abridged Cochrane systematic review and network meta-analysis. *BMJ* 2016;**353**:i1777.http://www.ncbi.nlm.nih.gov/pubmed/27102806

50 Ramanan A V, Dick AD, Jones AP, *et al.* Adalimumab plus Methotrexate for Uveitis in Juvenile Idiopathic Arthritis. *N Engl J Med* 2017;**376**:1637–46. doi:10.1056/NEJMoa1614160

51 Kiss S, Ahmed M, Letko E, *et al.* Long-term follow-up of patients with birdshot retinochoroidopathy treated with corticosteroid-sparing systemic immunomodulatory therapy. *Ophthalmology* 2005;**112**:1066–71. doi:10.1016/j.ophtha.2004.12.036

52 Biomarkers Definitions Working Group. Biomarkers and surrogate endpoints: preferred definitions and conceptual framework. *Clin Pharmacol Ther* 2001;**69**:89–95. doi:10.1067/mcp.2001.113989

53 Gisbert R, Brosa M. Spanish Health Costs and cost-effectiveness ratios Database: eSalud. Barcelona Oblik. Consult. S.L. 2022.http://www.oblikue.com/bddcostes/

54 Spanish Health Ministry. RAE-CMBD. MBDS: Minimum Basic Data Set of the Spanish Heath Ministry. 2022.https://pestadistico.inteligenciadegestion.mscbs.es/publicoSNS/N/rae-cmbd/rae-cmbd

55 LaMattina KC, Escott SM, Malalis JF, *et al.* The Influence of Race and Sex on the Side Effect Profile of Methotrexate in the Treatment of Uveitis. *Ocul Immunol Inflamm* 2018;:1–5. doi:10.1080/09273948.2018.1498905

For peer review only

56 López-Rodríguez R, Ferreiro-Iglesias A, Lima A, *et al.* Replication study of polymorphisms associated with response to methotrexate in patients with rheumatoid arthritis. *Sci Rep* 2018;**8**:7342. doi:10.1038/s41598-018-25634-y

57 Bek S, Bojesen AB, Nielsen J V, *et al.* Systematic review and meta-analysis: pharmacogenetics of anti-TNF treatment response in rheumatoid arthritis. *Pharmacogenomics J* 2017;**17**:403–11. doi:10.1038/tpj.2017.26

58 Fernández-Puente P, Mateos J, Fernández-Costa C, *et al.* Identification of a panel of novel serum osteoarthritis biomarkers. *J Proteome Res* 2011;**10**:5095–101. doi:10.1021/pr200695p

59 Lourido L, Calamia V, Mateos J, *et al.* Quantitative proteomic profiling of human articular cartilage degradation in osteoarthritis. *J Proteome Res* 2014;**13**:6096–106. doi:10.1021/pr501024p

60 Lourido L, Calamia V, Fernández-Puente P, *et al.* Secretome analysis of human articular chondrocytes unravels catabolic effects of nicotine on the joint. *Proteomics Clin Appl* 2016;**10**:671–80. doi:10.1002/prca.201400186

61 Fernández-Puente P, Calamia V, González-Rodríguez L, *et al.* Multiplexed mass spectrometry monitoring of biomarker candidates for osteoarthritis. *J Proteomics* 2017;**152**:216–25. doi:10.1016/j.jprot.2016.11.012

62 Lourido L, Ayoglu B, Fernández-Tajes J, *et al.* Discovery of circulating proteins associated to knee radiographic osteoarthritis. *Sci Rep* 2017;**7**:137. doi:10.1038/s41598-017-00195-8

63 Drobin K, Nilsson P, Schwenk JM. Highly multiplexed antibody suspension bead arrays for plasma protein profiling. *Methods Mol Biol* 2013;**1023**:137–45. doi:10.1007/978-1-4614-7209-4_8

64 Harris PA, Taylor R, Thielke R, *et al.* Research electronic data capture

For peer review only - http://bmjopen.bmj.com/site/about/guidelines.xhtml

(REDCap)--a metadata-driven methodology and workflow process for providing translational research informatics support. *J Biomed Inform* 2009;**42**:377–81. doi:10.1016/j.jbi.2008.08.010

65 Harris PA, Taylor R, Minor BL, *et al.* The REDCap consortium: Building an international community of software platform partners. *J Biomed Inform* 2019;**95**:103208. doi:10.1016/j.jbi.2019.103208

66 Jaffe GJ, Dick AD, Brézin AP, *et al.* Adalimumab in Patients with Active Noninfectious Uveitis. *N Engl J Med* 2016;**375**:932–43. doi:10.1056/NEJMoa1509852

67 NCT01829295: Methotrexate and Mycophenolate Mofetil for UVEITIS (FAST). https://clinicaltrials.gov/ct2/show/study/NCT01829295?cond=Uveitis&draw=2&rank=35

68 Samson CM, Waheed N, Baltatzis S, *et al.* Methotrexate therapy for chronic noninfectious uveitis: Analysis of a case series of 160 patients. *Ophthalmology* 2001;**108**:1134–9.

69 Galor A, Jabs DA, Leder HA, *et al.* Comparison of antimetabolite drugs as corticosteroid-sparing therapy for noninfectious ocular inflammation. *Ophthalmology* 2008;**115**:1826–32. doi:10.1016/j.ophtha.2008.04.026

70 Martínez C, Ortiz AM, Juarranz Y, *et al.* Serum levels of vasoactive intestinal peptide as a prognostic marker in early arthritis. *PLoS One* 2014;**9**:e85248. doi:10.1371/journal.pone.0085248

71 Abasolo L, Ivorra-Cortes J, Leon L, *et al.* Contribution of the bone and cartilage/soft tissue components of the joint damage to the level of disability in rheumatoid arthritis patients: a longitudinal study. *Clin Rheumatol* Published Online First: 16 October 2018. doi:10.1007/s10067-018-4335-4

For peer review only

72 Madrid-García A, Font-Urgelles J, Vega-Barbas M, *et al.* Outpatient Readmission in Rheumatology: A Machine Learning Predictive Model of Patient's Return to the Clinic. *J Clin Med* 2019;**8**. doi:10.3390/jcm8081156

For peer review only

For peer review only - http://bmjopen.bmj.com/site/about/guidelines.xhtml

FIGURE LEGENDS

Figure 1: Overview of the study timeline and interventions.

ACKNOWLEDGEMENTS

Sponsor

Fundación para la Investigacion Biomédica del Hospital Clínico San Carlos. The study sponsor had no role in the study design; collection, management, analysis and interpretation of data; writing of the report and the decision to submit the report for publication.

Committees

Executive Committee: Administrative and executive arm of the clinical trial, providing overall oversight for the study and making decisions on day-to-day operational issues [Study Coordinator (Luis Rodriguez-Rodriguez), a representative from the Spanish Clinical Trial Network (Amanda López Picado), and 5 Site Directors (these seats will be rotatory, with changes every 6 months)]; Data Coordinating and Analysis Committee: Supervising data collection,management and quality control, designing the statistical analysis plan, performing unmasked data analysis, and preparing interim and final reports for the Data Security Monitoring Board and the Executy Committee [Study Coordinator (Luis Rodriguez-Rodriguez), a representative from the Spanish Clinical

For peer review only - http://bmjopen.bmj.com/site/about/guidelines.xhtml

Trial Network (Amanda López Picado), and Ester Carreño]; Biobank and Biomarker Identification Committee (Maintaining an up-to-date manual of operations for blood extraction, processing and storage, and monitoring procedures adherence, supervising biological sample collection, sample shipment coordination, coordinating the phamacogenetic and proteomic analysis [Study Coordinator (Luis Rodriguez-Rodriguez), a representative from the Instituto de Salud Carlos III Biobank Platform (Elena Molino), a representative the Instituto de Investigación Biomédica de A Coruña, a representative from, the Data Coordinating and Analysis Committee]; Data Security Monitoring Committee (PierGiogio Neri, Andrew Dick, Loreto Carmona).

Patient consent for publication

Not required.

CoTHEIA Study Group

Mara Albert Fort (Hospital Universitario Doctor Peset), Mayte Ariño Gutierrez (Hospital Clínico San Carlos), Pedro Arriola Villalobos (Hospital Clínico San Carlos), Joseba Artaraz Beobide (Hospital Universitario Cruces), Antonio Atanes Sandoval (Complejo Hospitalario Universitario A Coruña), Jose Manuel Benitez Del Castillo Sanchez (Hospital Clínico San Carlos), Francisco Blázquez (Instituto Universitario de Oftalmología Aplicada, Universidad de Valladolid), Pablo Borges Deniz (Hospital Universitario Fundación Jiménez Díaz), Lara Borrego Sanz (Hospital Clínico San Carlos), Gabriela Bustamante Sanchez-Arnedo (Instituto Universitario de Oftalmología Aplicada, Universidad de Valladolid), Bruno Casco Silva (Complejo Hospitalario Universitario de Toledo), Ricardo Cuiña Sardiña (Hospital Clínico San Carlos), Almudena De Pablo Cabrera (Hospital Universitario 12 de Octubre), Maria Del Mar

For peer review only - http://bmjopen.bmj.com/site/about/guidelines.xhtml

Esteban Ortega (Hospital Universitario Infanta Sofía), Patricia Fernandez Puente (Instituto de Investigación Biomédica de A Coruña), Dalifer Dayanira Freites Nuñez (Hospital Clínico San Carlos), Javier Garcia Bella (Hospital Clínico San Carlos), Sara Garcia Carazo (Hospital Universitario La Paz), Luis Garcia Onrubia (Instituto Universitario de Oftalmología Aplicada, Universidad de Valladolid), Jose Maria Garcia Ruiz De Morales (Complejo Asistencial Universitario de León), Jose Antonio Gegundez Fernandez (Hospital Clínico San Carlos), Vanesa Hernandez Hernandez (Complejo Hospitalario Universitario de Canarias), Inés Hernanz Rodriguez (Hospital Universitario Fundación Jiménez Díaz), Jose Maria Herreras Cantalapiedra (Instituto Universitario de Oftalmología Aplicada, Universidad de Valladolid), Ventura Hidalgo Barrero (Hospital Universitario La Paz), Maria De La Vega Jovani Casano (Hospital General Universitario de Alicante), Maria Isabel Lopez Rodriguez (Complejo Hospitalario Universitario A Coruña), Sara López Sierra (Hospital Universitario Doctor Peset), Virginia Lozano Lopez (Complejo Hospitalario Universitario de Canarias), Alfredo Madrid Garcia (Hospital Clínico San Carlos), Rosalia Mendez Fernandez (Hospital Clínico San Carlos), Pilar Nozal Aranda (Hospital Universitario La Paz), Eugenio Perez Blazquez (Hospital Universitario 12 de Octubre), Chamaida Plasencia Rodriguez (Hospital Universitario La Paz), Sheila Recuero Diaz (Hospital Universitario Fundación Jiménez Díaz), Ignacio Robles Barrena (Hospital Universitario Rey Juan Carlos), Mª Esther Rodriguez Almaraz (Hospital Universitario 12 de Octubre), Fayna Maria Rodriguez Gonzalez (Hospital Universitario de Gran Canaria Doctor Negrín), Guadalupe Rodriguez Martinez (Complejo Hospitalario Universitario A Coruña), Fredeswinda Isabel Romero Bueno (Hospital Universitario Fundación Jiménez Díaz), Ioana Ruiz Arruza (Hospital Universitario Cruces), Beatriz Sanchez Marugan (Hospital Universitario Infanta Leonor), Olga Sanchez Pernaute (Hospital Universitario

For peer review only - http://bmjopen.bmj.com/site/about/guidelines.xhtml

Fundación Jiménez Díaz), Armelle Schlinker Giraud (Hospital Universitario La Paz), Alvaro Seijas Lopez (Complejo Hospitalario Universitario A Coruña), Patricia Simon Alonso (Complejo Hospitalario Universitario A Coruña), Marta Tejera Santana (Hospital Universitario de Gran Canaria Doctor Negrín), Elia Valls Pascual (Hospital Universitario Doctor Peset).

For peer review only

For peer review only - http://bmjopen.bmj.com/site/about/guidelines.xhtml

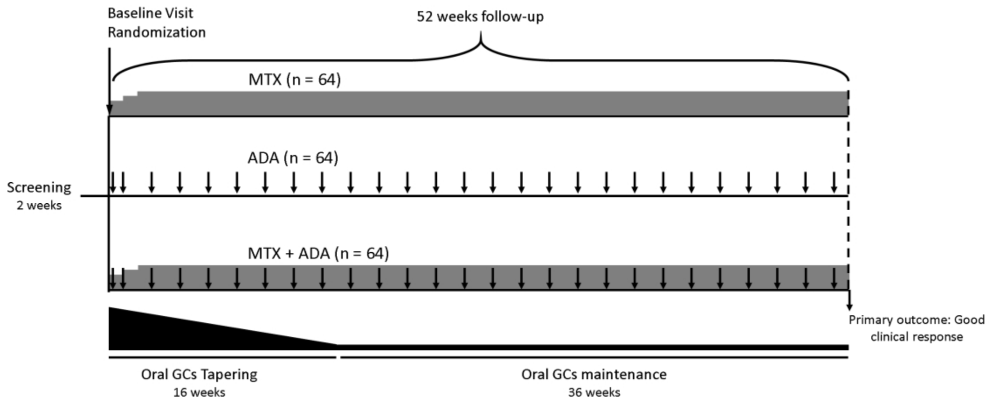

Overview of the study timeline and interventions

HOJA DE INFORMACIÓN AL PACIENTE

Solicitamos su participación en un proyecto de investigación promovido por investigadores de su centro y dirigido por el doctor Luis Rodriguez del Hospital Clínico San Carlos (Madrid). El estudio se titula **"Eficacia, seguridad y coste-efectividad del metotrexato, adalimumab, o su combinación en uveítis no anterior no infecciosa: un estudio multicéntrico, aleatorizado, paralelo de 3 brazos, con control activo, de fase 3, abierto, con evaluador cegado: Co-THEIA (CombinationTHerapy with mEthotrexate and adalImumAb for uveitis)"**

Siguiendo los estándares internacionalmente reconocidos y la normativa legal vigente en España sobre investigación, este ensayo clínico cumple todas las exigencias legales y ha sido aprobado por el Comité Ético de Investigación con medicamentos del Hospital Clínico San Carlos así como por la Agencia Española de Medicamentos y Productos Sanitarios.

Sin embargo, antes de aceptar participar o no, es importante que lea detenidamente la siguiente información y que realice todas las preguntas y aclare todas las cuestiones que crea conveniente con un familiar/amigo o con su médico.

¿Tengo que participar?

La participación en el estudio es completamente voluntaria. Rechazarla no acarreará ningún deterioro en la calidad de la asistencia ni en su atención médica. Además, podrá retirarse en cualquier momento del estudio sin tener que dar explicaciones y sin que ello repercuta en los

For peer review only - http://bmjopen.bmj.com/site/about/guidelines.xhtml

cuidados médicos que reciba. Su médico también podrá decidir, por alguna consideración médica o por cualquier otra razón, retirarle del estudio si así lo cree conveniente.

Así mismo, el hecho de que usted participe en este estudio no altera de ninguna manera ni sus tratamientos habituales ni el seguimiento de las enfermedades que tenga (en caso de que las tuviera).

¿En qué consiste el estudio y cómo se va a llevar a cabo?

Su médico le ha diagnosticado una uveítis no infecciosa, una inflamación de una parte del ojo que no está causada por un microorganismo (bacteria, hongo o virus).

Para tratar esta inflamación es posible que su oftalmólogo y/o reumatólogo/inmunólogo/especialista en Medicina Interna ya le haya pautado distintos tratamientos, principalmente corticoides. Sin embargo, estos fármacos a veces no son suficientes para controlar enfermedad. Por otro lado, es posible que haya sido diagnosticado/a de algún tipo de uveitis que se asocie a una inflamación más persistente. En ambos casos, en la actualidad disponemos de distintos medicamentos para el tratamiento pero solo tiene indicación aprobada el adalimumab. En este estudio vamos a comparar este medicamento y el metotrexato, solos o combinados.

For peer review only - http://bmjopen.bmj.com/site/about/guidelines.xhtml

El metotrexato es un fármaco que inhibe el sistema inmunitario y esto permite reducir la inflación del ojo. Se lleva utilizando desde hace más de 50 años en el tratamiento de otras enfermedades inflamatorias crónicas y más reccientemente (más de 30 años) en el tratamiento de las uveitis. Se puede tomar en pastillas o inyectado en la piel (vía subcutánea), su médico según su caso le indicara como y cuando tomarlo.

El adalimumab es otro medicamente que también inhibe el sistema inmunitario y reduce la inflamación, pero utiliza otro mecanismo de acción. Se lleva utilizando en el tratamiento de las uveitis desde hace más de 10 años, administrándose por vía subcutánea.

Si usted decide participar en el estudio podrá ser asignado a uno de los tres grupos en estudio: metotrexato, adalimumab y ambos (metotrexato+adalimumab). La asignación será al azar, es decir ni usted ni su médico decidirá que tratamiento recibe. Indistintamente el grupo al que sea asignado, las visitas y las pruebas que se le realicen serán las que habitualmente se realizan a pacientes como usted. Además, en el caso de que haya sido diagnosticado de algunos subtipos de uveítis (como es la uveítis intermedia), se le realizará una resonancia magnética cerebral con gadolinio antes de iniciar el estudio. Esto es debido a que estos subtipos de uveitis podrían estar asociados a la presencia de enfermedades desmielinizantes del sistema nervioso central, en las que el uso de adalimumab se encuentra contraindicado.

Usted tendrá acudir cada 4 semanas al centro. En estas visitas su médico se encargará revisar la situación del ojo (examen del ojo, agudeza visual…), los eventos adversos y la adherencia al

For peer review only - http://bmjopen.bmj.com/site/about/guidelines.xhtml

tratamiento. Además, deberá completar algunos cuestionarios para evaluar su calidad de vida, los costes y los fármacos que toma (aproximadamente 15 minutos). Para completar estos cuestionarios dispondrá de ayuda del personal del centro.

Adicionalmente le solicitaremos permiso para la extracción de unas muestras de sangre al inicio, tras 16 semanas, cuando finalice y en el caso de que tuviera algún brote de la uveítis durante el estudio. Estas muestras serán utilizadas para hacer análisis genéticos y de proteómica para identificar marcadores que permitan conocer más su enfermedad y /o que puedan estar asociados a una mejor respuesta al tratamiento. También, en el caso de que fuera a recibir adalimumab, para medir los niveles en sangre de ese fármaco. Por último, con el objetivo de llevar a cabo futuros estudios que nos ayuden a conocer mejor la enfermedad que usted padece, parte de estas muestras serán depositadas y almacenadas en el Biobanco vinculado a su centro en régimen de Biobanco (por ello, en el caso de acceder a que podamos extraer estas muestras, durante el proceso asistencial, se le facilitará para su revisión y firma una copia del consentimiento informado del Biobanco vinculado a su centro). Un Biobanco es un establecimiento de almacenamiento de muestras de origen humano bajo criterios de calidad, orden y destino para su utilización en investigaciones nacionales o internacionales dentro del campo de la biomedicina. Su funcionamiento se centra en gestionar, bajo criterios de seguridad, calidad y eficiencia; la recepción, procesamiento, almacenamiento y posterior cesión de muestras a los investigadores solicitantes, para que utilicen las mismas en sus proyectos de investigación; siempre y cuando, éstos cumplan todos los requisitos éticos y legales vigentes, tal y como establece el Real Decreto 1716/2011 de 18 de noviembre, la Ley de Investigación Biomédica 14/2007, (LIBM) y la normativa que la complementa. Una vez depositadas las muestras en el Biobanco, estas pueden ser cedidas a los investigadores que hayan pasado la

For peer review only - http://bmjopen.bmj.com/site/about/guidelines.xhtml

aprobación de un Comité de Ética de Investigación Clínica y del Comité de Ética y del Comité Científico del Biobanco, tal y como se establece en la normativa aplicable (Real Decreto 1716/2011 de 18 de noviembre y la LIBM.

La donación de estas muestras no impedirá que usted o su familia puedan usarlas cuando sea necesario por motivos de salud, siempre que estén disponibles.

¿Puedo restringir el uso que se le de las muestras que me sean extraídas?

Usted puede indicarnos si quiere establecer algún tipo de restricción sobre sus muestras y datos, en relación con su posible uso en determinados proyectos de investigación o en cuanto a determinadas cesiones. Para ello dispone de un apartado específico en la hoja de firma del consentimiento informado.

¿Cómo sabré en que se usan mis muestras?

El Biobanco y/o la persona responsable de la investigación tendrán a disposición de los participantes la información sobre los proyectos de investigación en los que se utilicen las muestras y datos.

En determinadas circunstancias el Comité de Ética competente podrá decidir si es necesario contactar con el participante para facilitarle información de manera individualizada.

De producirse un eventual cierre del Biobanco o revocación de la autorización para su constitución y funcionamiento, la información sobre el destino de las muestras estará a su disposición en el Registro Nacional de Biobancos para Investigación Biomédica del Instituto de

For peer review only

Salud Carlos III (ISC III), con página web www.isciii.es, con el fin de que pueda manifestar su conformidad o disconformidad con el destino previsto para las muestras.

¿Y si obtienen alguna información clínicamente relevante e inesperada como resultado del análisis de mis muestras?

Existe la posibilidad de que las muestras que han sido cedidas al Biobanco sean utilizadas en **estudios de biología celular, molecular y/o genéticos**. En ocasiones, en este tipo de estudios se puede descubrir información no buscada que puede ser relevante para su salud o la de su familia. Si ese fuera el caso, los resultados obtenidos serán validados y analizados por profesionales y por un Comité de Ética para determinar si son fiables en un porcentaje óptimo que aconseje su comunicación a las personas afectadas.

Usted debe saber que tiene derecho a conocer, o no, la información obtenida con el análisis de sus muestras. En el caso de que usted decida no ser informado, la ley establece que cuando la información obtenida sea necesaria para evitar un grave perjuicio para la salud de sus familiares, un Comité de expertos estudiará el caso y deberá decidir entre la conveniencia o no de informar a los afectados o a sus representantes legales.

¿Qué beneficios puedo esperar por el hecho de participar en el estudio?

Puede ocurrir que usted no obtenga ningún beneficio personal de la participación en este proyecto de investigación. En cualquier caso, su participación en este estudio contribuirá al avance del conocimiento sobre el tratamiento de la uveítis no infecciosa.

For peer review only - http://bmjopen.bmj.com/site/about/guidelines.xhtml

¿Qué incomodidades o riesgos puede suponer mi participación en el estudio?

Entre los efectos secundarios más habituales del metotrexato están el dolor de cabeza, los moratones y sangrados, el aumento de las infecciones, las náuseas y vómitos, la diarrea, los dolores musculares, el cansancio, la pérdida de cabello y la afectación hepática.

Por su parte, el adalimumab presenta los siguientes efectos secundarios: molestias o dolor en el punto de inyección, erupción cutánea, dolor de cabeza e infecciones (siendo las más habituales las de las vías respiratorias altas (catarros entre otros) y la sinusitis).

En el caso de administrar de manera conjunta ambos fármacos los efectos adversos podrían ser más intensos y aparecer algunos desconocidos. En este momento, la administración de estos dos medicamentos de manera conjunta no está autorizada.

En el caso de que se le tuviese que realizar una resonancia nuclear magnética con gadolinio, el uso de este contraste puede verse asociado a eventos adverso agudos en 1 de cada 1000 individuos, siendo los más frecuentes de la aparición de rash, urticaria y nauseas. Además, ha sido descrita la posibilidad de depósito de esta substancia a nivel cerebral y en otros tejidos, tras administraciones repetidas.

¿Cuáles son mis derechos y cómo van a ser tratados mis datos?

Toda la información que se registrará de usted será estrictamente confidencial. De acuerdo con el Reglamento General de Protección de Datos (RGPD) (Reglamento (EU) 2016/679), además de los derechos de acceso, rectificación, oposición y cancelación de datos (Ley Orgánica 3/2018, de

For peer review only - http://bmjopen.bmj.com/site/about/guidelines.xhtml

5 de diciembre, de Protección de Datos Personales y garantía de los derechos digitales), también tiene derecho a limitar el tratamiento de datos y solicitar una copia o que se trasladen a un tercero (portabilidad) los datos que usted ha facilitado para el estudio. Para ejercitar sus derechos, diríjase al investigador principal del estudio. No obstante, le recordamos que los datos no se pueden eliminar, aunque deje de participar en el ensayo para garantizar la validez de la investigación y cumplir con los deberes legales y los requisitos de autorización de medicamentos. Así mismo tiene derecho a dirigirse a la Agencia de Protección de Datos si no quedara satisfecho.

En todo momento se mantendrá el anonimato de los pacientes y, para ello, en ninguno de los documentos del estudio aparecerá su nombre, sino que le será asignado un número que será el que se utilice en todos ellos. En todos los informes escritos y publicaciones, sólo aparecerá su número de referencia. Sólo el médico responsable del estudio guardará, en condiciones de seguridad, la lista que relaciona los nombres de los pacientes con los números de referencia asignados a cada uno.

Sólo tendrán acceso a los datos del estudio el equipo investigador y el monitor del estudio, miembros del comité, autoridades sanitarias competentes y/o de la agencia reguladora (AEMPS) para asegurar que el estudio se está llevando a cabo con las leyes vigentes y la reglamentación sanitaria. Firmando este documento, usted está autorizando este acceso. Además, los resultados del estudio siempre serán presentados de manera global y nunca de forma individualizada.

For peer review only

For peer review only - http://bmjopen.bmj.com/site/about/guidelines.xhtml

El Investigador y el Promotor están obligados a conservar los datos recogidos para el estudio al menos hasta 25 años tras su finalización. Posteriormente, su información personal solo se conservará por el centro para el cuidado de su salud y por el promotor para otros fines de investigación científica si usted hubiera otorgado su consentimiento para ello, y si así lo permite la ley y requisitos éticos aplicables.

Si realizáramos transferencia de sus datos codificados fuera de la UE a las entidades de nuestro grupo, a prestadores de servicios o a investigadores científicos que colaboren con nosotros, los datos del participante quedarán protegidos con salvaguardas tales como contratos u otros mecanismos por las autoridades de protección de datos. Si el participante quiere saber más al respecto, puede contactar al Delegado de Protección de Datos del promotor o Investigador Principal.

¿Mi participación supondrá algún coste o compensación económica?

Su participación en este estudio no supondrá para usted ningún coste económico, así como tampoco será recompensado económicamente por ello. El estudio dispone de una póliza de seguros que se ajusta a la legislación vigente (Real decreto 1090/2015) y que le proporcionará la compensación e indemnización en caso de menoscabo de su salud o de lesiones que pudieran producirse en relación con su participación en el estudio, siempre que no sean consecuencia de la propia enfermedad que se estudia o de la evolución propia de su enfermedad como consecuencia de la ineficacia del tratamiento. Este estudio se considera de Bajo Nivel de

For peer review only - http://bmjopen.bmj.com/site/about/guidelines.xhtml

Intervención (estudios de bajo riesgo, muy parecidos a la práctica clínica habitual), por lo que la póliza con la que está usted cubierto es la póliza de Sistema de Salud.

Si usted tiene alguna duda o quiere más información, no dude en consultar con el médico responsable que le está solicitando este consentimiento.

¿Cómo obtener información adicional?

Si desea información adicional sobre el estudio puede contactar con:

____

GRACIAS POR LEER ESTA INFORMACIÓN

For peer review only - http://bmjopen.bmj.com/site/about/guidelines.xhtml

CONSENTIMIENTO INFORMADO PARA LOS PACIENTES

Título del estudio: **"Eficacia, seguridad y coste-efectividad del metotrexato, adalimumab, o su combinación en uveítis no anterior no infecciosa: un estudio multicéntrico, aleatorizado, paralelo de 3 brazos, con control activo, de fase 3, abierto, con evaluador cegado: Co-THEIA (CombinationTHerapy with mEthotrexate and adalImumAb for uveitis)"**

Yo……………………………………………………………………………………………..

con DNI/NIF: ……………………………

(Nombre, apellidos y número de identificación del participante, puño y letra del paciente)

DECLARO:

- Que he hablado con ……………………………………………….

(Nombre del médico, puño y letra del paciente)

- Que he leído la hoja de información que se me ha entregado sobre el estudio.
- He comprendido la información recibida y he podido formular todas las preguntas que he creído oportunas.
- Comprendo que mi participación es voluntaria y que en cualquier momento puedo revocar mi consentimiento sin tener que dar explicaciones y sin que afecte en ningún aspecto a mi relación con el personal médico ni a la atención recibida por su parte.
- He recibido una copia firmada y fechada de este Consentimiento Informado.

Presto libremente mi conformidad a participar en este estudio y doy mi consentimiento para:

La obtención de las muestras indicadas en la Hoja de Información que se me han facilitado y su cesión al Biobanco vinculado a su centro, en las condiciones indicadas, para su utilización en cualquier investigación biomédica	Si	No
Ser informado de los resultados de las investigaciones que sean de interés para mi salud<br>Teléfono de Contacto ………………………………………	Si	No
Deseo indicar restricciones al uso de los datos o de las muestras que me sean extraídas ………………………………………………………………………….<br>…………………………………………………………………………<br>………………………………………………………………………….		

For peer review only - http://bmjopen.bmj.com/site/about/guidelines.xhtml

EN CONSECUENCIA, DOY MI CONSENTIMIENTO PARA FORMAR PARTE DE ESTE PROYECTO DE INVESTIGACION.

Firma del paciente:

Nombre y apellidos:

……………………………………………

Fecha: ……………………………

Firma del médico responsable:

Nombre y apellidos:

…………………………………………….

Fecha: ……………………………

For peer review only - http://bmjopen.bmj.com/site/about/guidelines.xhtml

Reporting checklist for protocol of a clinical trial.

Based on the SPIRIT guidelines.

Instructions to authors

Complete this checklist by entering the page numbers from your manuscript where readers will find each of the items listed below.

Your article may not currently address all the items on the checklist. Please modify your text to include the missing information. If you are certain that an item does not apply, please write "n/a" and provide a short explanation.

Upload your completed checklist as an extra file when you submit to a journal.

In your methods section, say that you used the SPIRITreporting guidelines, and cite them as:

Chan A-W, Tetzlaff JM, Gøtzsche PC, Altman DG, Mann H, Berlin J, Dickersin K, Hróbjartsson A, Schulz KF, Parulekar WR, Krleža-Jerić K, Laupacis A, Moher D. SPIRIT 2013 Explanation and Elaboration: Guidance for protocols of clinical trials. BMJ. 2013;346:e7586

		Reporting Item	Page Number
**Administrative information**			
Title	#1	Descriptive title identifying the study design, population, interventions, and, if applicable, trial acronym	1
Trial registration	#2a	Trial identifier and registry name. If not yet registered, name of intended registry	21
Trial registration: data set	#2b	All items from the World Health Organization Trial Registration Data Set	22
Protocol version	#3	Date and version identifier	21
Funding	#4	Sources and types of financial, material, and other support	32
Roles and responsibilities: contributorship	#5a	Names, affiliations, and roles of protocol contributors	32

For peer review only

Roles and responsibilities: sponsor contact information	#5b	Name and contact information for the trial sponsor	33
Roles and responsibilities: sponsor and funder	#5c	Role of study sponsor and funders, if any, in study design; collection, management, analysis, and interpretation of data; writing of the report; and the decision to submit the report for publication, including whether they will have ultimate authority over any of these activities	33
Roles and responsibilities: committees	#5d	Composition, roles, and responsibilities of the coordinating centre, steering committee, endpoint adjudication committee, data management team, and other individuals or groups overseeing the trial, if applicable (see Item 21a for data monitoring committee)	33
**Introduction**			
Background and rationale	#6a	Description of research question and justification for undertaking the trial, including summary of relevant studies (published and unpublished) examining benefits and harms for each intervention	7
Background and rationale: choice of comparators	#6b	Explanation for choice of comparators	6
Objectives	#7	Specific objectives or hypotheses	9
Trial design	#8	Description of trial design including type of trial (eg, parallel group, crossover, factorial, single group), allocation ratio, and framework (eg, superiority, equivalence, non-inferiority, exploratory)	9
**Methods: Participants, interventions, and outcomes**			
Study setting	#9	Description of study settings (eg, community clinic, academic hospital) and list of countries where data will be collected. Reference to where list of study sites can be obtained	11
Eligibility criteria	#10	Inclusion and exclusion criteria for participants. If applicable, eligibility criteria for study centres and individuals who will	11

For peer review only

		perform the interventions (eg, surgeons, psychotherapists)	
Interventions: description	#11a	Interventions for each group with sufficient detail to allow replication, including how and when they will be administered	12
Interventions: modifications	#11b	Criteria for discontinuing or modifying allocated interventions for a given trial participant (eg, drug dose change in response to harms, participant request, or improving / worsening disease)	15
Interventions: adherance	#11c	Strategies to improve adherence to intervention protocols, and any procedures for monitoring adherence (eg, drug tablet return; laboratory tests)	17
Interventions: concomitant care	#11d	Relevant concomitant care and interventions that are permitted or prohibited during the trial	12
Outcomes	#12	Primary, secondary, and other outcomes, including the specific measurement variable (eg, systolic blood pressure), analysis metric (eg, change from baseline, final value, time to event), method of aggregation (eg, median, proportion), and time point for each outcome. Explanation of the clinical relevance of chosen efficacy and harm outcomes is strongly recommended	9
Participant timeline	#13	Time schedule of enrolment, interventions (including any run-ins and washouts), assessments, and visits for participants. A schematic diagram is highly recommended (see Figure)	15
Sample size	#14	Estimated number of participants needed to achieve study objectives and how it was determined, including clinical and statistical assumptions supporting any sample size calculations	17
Recruitment	#15	Strategies for achieving adequate participant enrolment to reach target sample size	11
**Methods: Assignment of interventions (for controlled trials)**			
Allocation: sequence generation	#16a	Method of generating the allocation sequence (eg, computer-generated random numbers), and list of any factors for stratification. To reduce predictability of a random sequence, details of any planned restriction (eg, blocking) should be provided in a separate document that is unavailable to those who enrol participants or assign interventions	12

For peer review only

Allocation concealment mechanism	#16b	Mechanism of implementing the allocation sequence (eg, central telephone; sequentially numbered, opaque, sealed envelopes), describing any steps to conceal the sequence until interventions are assigned	12
Allocation: implementation	#16c	Who will generate the allocation sequence, who will enrol participants, and who will assign participants to interventions	12
Blinding (masking)	#17a	Who will be blinded after assignment to interventions (eg, trial participants, care providers, outcome assessors, data analysts), and how	13
Blinding (masking): emergency unblinding	#17b	If blinded, circumstances under which unblinding is permissible, and procedure for revealing a participant's allocated intervention during the trial	13
**Methods: Data collection, management, and analysis**			
Data collection plan	#18a	Plans for assessment and collection of outcome, baseline, and other trial data, including any related processes to promote data quality (eg, duplicate measurements, training of assessors) and a description of study instruments (eg, questionnaires, laboratory tests) along with their reliability and validity, if known. Reference to where data collection forms can be found, if not in the protocol	10
Data collection plan: retention	#18b	Plans to promote participant retention and complete follow-up, including list of any outcome data to be collected for participants who discontinue or deviate from intervention protocols	11
Data management	#19	Plans for data entry, coding, security, and storage, including any related processes to promote data quality (eg, double data entry; range checks for data values). Reference to where details of data management procedures can be found, if not in the protocol	15
Statistics: outcomes	#20a	Statistical methods for analysing primary and secondary outcomes. Reference to where other details of the statistical analysis plan can be found, if not in the protocol	18
Statistics: additional analyses	#20b	Methods for any additional analyses (eg, subgroup and adjusted analyses)	18

For peer review only

Statistics: analysis population and missing data	#20c	Definition of analysis population relating to protocol non-adherence (eg, as randomised analysis), and any statistical methods to handle missing data (eg, multiple imputation)	17
**Methods: Monitoring**			
Data monitoring: formal committee	#21a	Composition of data monitoring committee (DMC); summary of its role and reporting structure; statement of whether it is independent from the sponsor and competing interests; and reference to where further details about its charter can be found, if not in the protocol. Alternatively, an explanation of why a DMC is not needed	16
Data monitoring: interim analysis	#21b	Description of any interim analyses and stopping guidelines, including who will have access to these interim results and make the final decision to terminate the trial	18
Harms	#22	Plans for collecting, assessing, reporting, and managing solicited and spontaneously reported adverse events and other unintended effects of trial interventions or trial conduct	15
Auditing	#23	Frequency and procedures for auditing trial conduct, if any, and whether the process will be independent from investigators and the sponsor	15
**Ethics and dissemination**			
Research ethics approval	#24	Plans for seeking research ethics committee / institutional review board (REC / IRB) approval	21
Protocol amendments	#25	Plans for communicating important protocol modifications (eg, changes to eligibility criteria, outcomes, analyses) to relevant parties (eg, investigators, REC / IRBs, trial participants, trial registries, journals, regulators)	21
Consent or assent	#26a	Who will obtain informed consent or assent from potential trial participants or authorised surrogates, and how (see Item 32)	21
Consent or assent: ancillary studies	#26b	Additional consent provisions for collection and use of participant data and biological specimens in ancillary studies, if applicable	22
Confidentiality	#27	How personal information about potential and enrolled participants will be collected, shared, and maintained in order to protect confidentiality before, during, and after the trial	22

For peer review only

Declaration of interests	#28	Financial and other competing interests for principal investigators for the overall trial and each study site	32
Data access	#29	Statement of who will have access to the final trial dataset, and disclosure of contractual agreements that limit such access for investigators	33
Ancillary and post trial care	#30	Provisions, if any, for ancillary and post-trial care, and for compensation to those who suffer harm from trial participation	15
Dissemination policy: trial results	#31a	Plans for investigators and sponsor to communicate trial results to participants, healthcare professionals, the public, and other relevant groups (eg, via publication, reporting in results databases, or other data sharing arrangements), including any publication restrictions	22
Dissemination policy: authorship	#31b	Authorship eligibility guidelines and any intended use of professional writers	32
Dissemination policy: reproducible research	#31c	Plans, if any, for granting public access to the full protocol, participant-level dataset, and statistical code	22
**Appendices**			
Informed consent materials	#32	Model consent form and other related documentation given to participants and authorised surrogates	14
Biological specimens	#33	Plans for collection, laboratory evaluation, and storage of biological specimens for genetic or molecular analysis in the current trial and for future use in ancillary studies, if applicable	10

The SPIRIT Explanation and Elaboration paper is distributed under the terms of the Creative Commons Attribution License CC-BY-NC. This checklist was completed on 16. March 2021 using https://www.goodreports.org/, a tool made by the EQUATOR Network in collaboration with Penelope.ai

For peer review only
